# *Pachybrachis* (Coleoptera, Chrysomelidae, Cryptocephalinae) of Eastern Canada

**DOI:** 10.3897/zookeys.332.4753

**Published:** 2013-09-19

**Authors:** Robert J. Barney, Laurent LeSage, Karine Savard

**Affiliations:** 1 Gus R. Douglass Land-Grant Institute, West Virginia State University, Institute, West Virginia, United States 25112-1000; 2 Agriculture and Agri-Food Canada, Eastern Cereal and Oilseed Research Centre, Neatby Building, Ottawa, Ontario, Canada K1A 0C6

**Keywords:** Coleoptera, Chrysomelidae, *Pachybrachis*, eastern Canada, taxonomy

## Abstract

Seventeen *Pachybrachis* species occurring in eastern Canada [Ontario (ON), Québec (QC), New Brunswick (NB), Nova Scotia (NS), and Prince Edward Island (PE)] are treated by the authors. Two new national records were discovered, both from southernmost Ontario: *P. cephalicus* Fall and *P. luctuosus* Suffrian. Four species were new provincial records: *P. cephalicus* (ON), *P. luctuosus* (ON, QC), *P. obsoletus* Suffrian (NB), *P. peccans* (PE). A fully illustrated key to the *Pachybrachis* of eastern Canada is provided and supported with extensive photographs, distribution maps, and plant associations.

Three species were distributed from southern Ontario into at least one province in the Maritimes (*P. nigricornis* (Say), *P. obsoletus* Suffrianand *P. peccans* Suffrian). Six species were distributed along the shores of the Great Lakes (Erie, Michigan, and Ontario) and rivers (Ottawa, Saguenay and St. Lawrence), but unknown from central and northern ON and QC (*P. bivittatus* (Say), *P. hepaticus hepaticus* (F. E. Melsheimer), *P. othonus othonus* (Say), *P. pectoralis* (F. E. Melsheimer), *P. spumarius* Suffrianand *P. trinotatus* (F. E. Melsheimer)). Seven species were rare, five being found exclusively from southern ON (*P. calcaratus* Fall, *P. cephalicus*, *P. luridus* (Fabricius), *P. subfasciatus* (J. E. LeConte)and *P. tridens* (F. E. Melsheimer)), with two having, in addition, a disjunct population in QC (*P. atomarius* (F. E. Melsheimer)and *P. luctuosus*). One species was found to be the northern most extension of an eastern United States (US) distribution into the eastern townships of QC (*P. m-nigrum* (F. E. Melsheimer)). There were no *Pachybrachis* that could be considered arctic, subarctic, or boreal species; no specimens were found from Labrador and Newfoundland, and all species had southern affinities. *Pachybrachis atomarius*, *P. calcaratus*, *P. luridus*, *P. subfaciatus*, and *P. tridens*, not seen over the last 30–70 years, may be extirpated from eastern Canada.

## Abstract (fr)

Les auteurs traitent des 17 espèces de *Pachybrachis* présentes dans l’est du Canada [Ontario (ON), Québec (QC), Nouveau-Brunswick (NB), Nouvelle-Écosse (NS), Île-du-Prince-Édouard (PE)]. Deux espèces en provenance de l’extrême sud de l’Ontario sont nouvelles pour le pays: *P. cephalicus* Fall et *P. luctuosus* Suffrian. Quatre espèces représentent de nouvelles mentions provinciales: *P. cephalicus* (ON), *P. luctuosus* (ON, QC), *P. obsoletus* Suffrian (NB) et *P. peccans* (PE). La clé d’identification des espèces de l’est du Canada s’appuie sur de nombreuses illustrations en plus d’inclure des cartes de répartition et les associations avec les plantes.

Trois espèces se répartissent du sud de l’ON jusqu’à au moins l’une ou l’autre des provinces Maritimes (*P. nigricornis* (Say), *P. obsoletus* Suffrianet *P. peccans* Suffrian). Six espèces sont limitées aux rives des Grands-Lacs (Érié, Michigan et Ontario), du fleuve Saint-Laurent et des grandes rivières (des Outaouais, Saguenay) sans atteindre le centre et le nord de l’ON et du QC (*P. bivittatus* (Say), *P. hepaticus* (F. E. Melsheimer), *P. othonus* (Say), *P. pectoralis* (F. E. Melsheimer), *P. spumarius* Suffrianet *P. trinotatus* (F. E. Melsheimer)). Cinq, parmi les sept espèces rares, proviennent exclusivement du sud de l’ON (*P. calcaratus*, *P. cephalicus*, *P. luridus* (Fabricius), *P. subfasciatus* (J. E. LeConte) et *P. tridens* (F. E. Melsheimer)), alors que les deux autres possèdent, en outre, une population disjointe au QC (*P. atomarius* et *P. luctuosus*). Une espèce, présente seulement en Estrie (QC), se trouve à la limite septentrionale de sa répartition dans l’est des États-Unis (*P. m-nigrum* (F. E. Melsheimer)). Aucune espèce, parmi les *Pachybrachis* étudiés, n’est arctique, subarctique ou boréale, mais toutes présentent des affinités méridionales. Aucun spécimen en provenance de Terre-Neuve et du Labrador n’a été observé. Les espèces *Pachybrachis atomarius*, *P. calcaratus*, *P. luridus*, *P. subfaciatus* et *P. tridens* n’ont pas été vues depuis les 30–70 dernières années de sorte qu’elles sont probablement disparues de la faune de l’Est du Canada.

## Introduction

In the *Catalog of Leaf Beetles of America North of Mexico* Riley et al. (2003) listed *Pachybrachis* (Chrysomelidae: Cryptocephalinae) as one of the genera most in need of taxonomic revision, because many species cannot be identified with confidence. The senior author of the current investigation has been struggling with *Pachybrachis* for over 30 years and recently began a systematic review of all specimens housed in public and private collections, with a goal of a revision of the species found within the eastern United States. This effort then expanded into eastern North America after the junior authors provided access to the major collections in Canada as part of their Canadian Beetles Project. This collaboration has resulted in this paper as a prelude to the more diverse and daunting challenge of the entire eastern North American *Pachybrachis* fauna.

## Historical review

When Johann Christian Fabricius (1798) described *Cryptocephalus luridus* from “America borealis”, the genus *Cryptocephalus* represented the entire Cryptocephalini tribe of today. The generic concept was even larger for Melsheimer (1806) in his *Catalogue of insects of Pennsylvania*, since several of the 28 species listed under *Cryptocephalus* are currently placed in non-cryptocephaline genera (*Glyptoscelis*, *Paria*, etc.) and only six belong to *Pachybrachis*. Chevrolat (1836) split this broad genus into several new genera, including *Pachybrachis*, to which *Pachybrachis luridus* was the first Nearctic species to be transferred by Suffrian (1852). Because this species was variable and common, it was subsequently described under several names which are all currently considered synonyms: *Cryptocephalus femoratus* Say, 1824; *Cryptocephalus aesculi* Melsheimer, 1847; *Pachybrachis moerens* Stål, 1857; and the varieties *Pachybrachis luridus nigrinus* Blatchley, 1910 and *Pachybrachis luridus festivus* Fall, 1915.

Thomas Say (1824) was one of the first North American entomologists to describe Nearctic insects. Under the old concept of *Cryptocephalus* Geoffroy, 1762, he described *Cryptocephalus bivittatus*, *Cryptocephalus nigricornis*, and *Cryptocephalus othonus*, three species currently placed in *Pachybrachis* Chevrolat, 1836 (Riley et al. 2003).

The same year, John Eatton LeConte (1824) described several new beetle species from Georgia (US) including *Cryptocephalus subfasciatus*, which was transferred to *Pachybrachis* by Haldeman (1849). F. E. Melsheimer (1847) described six new species of *Pachybrachis* (as *Cryptocephalus*) from Pennsylvania (US) that have subsequently been found in eastern Canada: *Pachybrachis atomarius*, *Pachybrachis hepaticus*, *Pachybrachis m-nigrum*, *Pachybrachis pectoralis*, *Pachybrachis tridens* and *Pachybrachis trinotatus*. Haldeman (1849) provided the first key to 16 species of *Pachybrachis*, including seven found in eastern Canada: *Pachybrachis bivittatus*, *Pachybrachis carbonarius* (= *Pachybrachis nigricornis*), *Pachybrachis luridus*, *Pachybrachis m-nigrum*, *Pachybrachis othonus*, *Pachybrachis subfasciatus*, and *Pachybrachis trinotatus*.

The beetles collected by the Swiss scientist Louis Agassiz and the Italian explorer John Cabot during the exploration of several regions of Lake Superior (ON) were studied and identified by LeConte (1850) who cited *Pachybrachis abdominalis* (probably *Pachybrachis peccans*) and *Pachybrachis m-nigrum* with no additional information.

The German entomologist Christian Wilhelm Ludwig Eduard Suffrian specialized in Coleoptera, especially Chrysomelidae. He described many North American species, including four in *Pachybrachis* that are found in eastern Canada: *Pachybrachis luctuosus*, *Pachybrachis obsoletus*, *Pachybrachis peccans*, and *Pachybrachis spumarius* (Suffrian 1852, 1858).

The *Catalogue of the described Coleoptera of the United States* (Melsheimer 1853, revised by S. S. Haldeman and J. L. LeConte) listed 20 species of *Pachybrachis*, eight of which are found in eastern Canada (*Pachybrachis atomarius*, *Pachybrachis bivittatus*, *Pachybrachis carbonarius* (= *Pachybrachis nigricornis carbonarius*), *Pachybrachis luridus*, *Pachybrachis m-nigrum*, *Pachybrachis othonus*, *Pachybrachis subfasciatus*, and *Pachybrachis trinotatus*), and four additional *Pachybrachis* species listed as *Cryptocephalus* (*Pachybrachis hepaticus*, *Pachybrachis nigricornis*, *Pachybrachis obsoletus*, and *Pachybrachis tridens*).

During the next 50 years, several Canadian collectors explored various areas of Ontario and Québec, but no records show that they were successful in collecting *Pachybrachis*: William Couper in Toronto (ON) and in various localities in the province of Québec (Couper 1855a, 1855b); Robert Bell (1858) in Gaspesia [list revisited by D’Urban (1859a)]; D’Urban (1859b, 1859c, 1860a, 1860b) in Argenteuil County (QC), in the vicinity of Montréal (QC), at Hudson Bay (QC) and in Ottawa (ON); Beadle (1861) in the county of Lincoln (ON); Couper (1864, 1865) in the vicinity of Toronto (ON) and in Québec City (QC); Saunders (1868) in the Saguenay region (QC); Ritchie (1869) on the Island of Montréal (QC); Reed (1869) in the neighbourhood of London (ON), listing only the families of collected beetles; Jones (1870) in Halifax (NS); Packard (1870) at Caribou Island (LB); Provancher (1870) at Portneuf (QC); Packard (1872), listing the beetles known to occur in Labrador and later adding spiders, myriapods and other groups of insects (Packard 1888); Couper (1874) in Anticosti Island (QC); Couper (1881, 1882, 1883) in the province of Québec in general; Bell (1881) at Belleville (ON); Harrington (1882) at Ottawa (ON) and at Chelsea, Hull and Montebello (QC); LeConte (1883), studying the specimens collected by J.T. Bell and others in various areas north of Lake Superior (ON); Fletcher (1888), identifying the specimens collected by J.S. Cotter and J.M. Macoun on the south coast and islands of James Bay (QC); Harrington (1890a, b, c), relisting several of the previous reports; Hausen (1893) at Saint-Jérome (QC); Hanham (1894) in Québec City and vicinity (QC); Harrington (1894a, b) at Ottawa (ON) and Copper Cliff (ON); Evans (1899) at Halifax (NS); Ouellet (1902) at Montréal, Rigaud and Joliette (QC); Evans (1905) at light at Trenton (ON); and Sherman (1910) in Labrador.

Pettit (1868–1872) listed 1290 species in a series of articles on beetles collected at Grimsby (ON), including three *Pachybrachis* species (*Pachybrachis mollis*, *Pachybrachis subfasciatus*, and *Pachybrachis tridens*). His *Pachybrachis mollis* is now considered a synonym of *Pachybrachis tridens* (Riley et al. 2003).

The priest Léon Provancher (1877a) was the first Canadian to describe and key out two *Pachybrachis* species (*Pachybrachis luridus*, *Pachybrachis othonus*) in his *Petite faune entomologique du Canada*, to which was added *Pachybrachis atomarius* (Provancher 1877b). It should be noted here that *Pachybrachis luridus* in the key was erroneously transcribed as *Pachybrachis lividus* in the description. This is an evident misspellingsince Fabricius never described any insect under such name (Zimsen 1964). Two years later, Provancher (1879) added *Pachybrachis litigiosus* from Saint-Hyacinthe.

William Couper (1883) listed three species collected in the province of Québec but did not specify the localities from where they were obtained: *Pachybrachis atomarius*, *Pachybrachis luridus*, and *Pachybrachis othonus*. Brodie and White (1883a, b) prepared a checklist of the insects of the Dominion of Canada (= Ontario and Québec) and listed nine names under *Pachybrachis*, but two were misidentified (*Pachybrachis litigiosus*, and *Pachybrachis morosus*).

In his list of the Ottawa Coleoptera, Harrington (1884) reported four species found in this locality: *Pachybrachis femoratus*, *Pachybrachis litigiosus*, *Pachybrachis tridens* and *Pachybrachis viduatus*. However, his report of *Pachybrachis femoratus* was most likely based on misidentified specimens of *Pachybrachis luridus*, his report of *Pachybrachis litigiosus* was probably based on misidentified specimens of the striped version of *Pachybrachis nigricornis difficilis*, and *Pachybrachis bivittatus* was often misidentified as *Pachybrachis viduatus*, which is a southern US species not found in Canada.

Henshaw (1885, 1887, 1889) updated the aging checklist of Melsheimer (revised by Haldeman and LeConte 1853) including its supplement (Austin 1880). At the beginning of the 20^th^ century, Clavareau (1913) published the first world catalogue of the subfamily Cryptocephalinae, including species in the genus *Pachybrachis*. As regards the eastern Canadian species, *Pachybrachis bivittatus* was given as a synonym of *Pachybrachis viduatus* (Fabricius 1801) and *Pachybrachis m-nigrum* as a synonym of *Pachybrachis picturatus* (Germar 1824); since, *Pachybrachis viduatus* and *Pachybrachis picturatus* are fairly rare species only found in the southern US, these two species were evidently misidentified. The same year, Weise (1913) proposed *Pachybrachis praeclarus* as a replacement name for *Pachybrachis elegans* Blatchley, 1910, since this name was already preoccupied by a *Pachybrachis* species described by Graëlls (1851) from Spain.

Evans (1895a, b) listed several beetle species collected in the district of Sudbury (ON), including *Pachybrachis femoratus* (= *Pachybrachis luridus*?) and *Pachybrachis infaustus* (= *Pachybrachis atomarius*); however, voucher specimens were not found for this study. In a series of papers on the Chrysomelidae of Ontario and Québec, Wickham (1896) provided a key to nine species of *Pachybrachis*, plus a description and illustration of beetles reported to be *Pachybrachis viduatus*, but that in actuality were clearly *Pachybrachis bivittatus*.

In his report on the insects of the Toronto region (ON), Walker (1913) listed *Pachybrachis atomarius*, *Pachybrachis femoratus* (= *Pachybrachis luridus*?), *Pachybrachis hepaticus*, *Pachybrachis othonus* and *Pachybrachis trinotatus*. Gibson (1915, 1919) reported several collection records of *Pachybrachis* in his reports to the Entomological Society of Ontario, but they were all from Manitoba.

During the same period, H. C. Fall (1915) thoroughly revised the Nearctic *Pachybrachis (Pachybrachys)*, and his revision is still, today, the only available complete taxonomic work on this genus. Out of the 159 recognized species, only six were given with a distribution extending into eastern Canada: *Pachybrachis luridus* (ON), *Pachybrachis othonus othonus* (MB, ON), *Pachybrachis peccans* (MB, NB, ON), *Pachybrachis relictus* (ON), *Pachybrachis subfasciatus* (ON), and *Pachybrachis trinotatus* (ON), with *Pachybrachis calcaratus* located in nearby Detroit (USA, Michigan). The well-known *Catalogue of the Coleoptera of America, north of Mexico* of Leng (1920) relisted the species treated by Fall (1915), but the distributions were reduced to a few state records or short statements, with the result that only *Pachybrachis peccans* was clearly reported from New Brunswick. No relevant information on *Pachybrachis* was added in the supplements, except the record of *Pachybrachis donneri* from Oregon (Leng and Mutchler 1927, 1933; Blackwelder 1939; Blackwelder and Blackwelder 1948).

Gustave Chagnon (1917) treated the Coleoptera of Québec in the third part of a preliminary list of the insects of this province. Eight species of *Pachybrachis* were collected in Montréal or in neighboring localities: *Pachybrachis atomarius*, *Pachybrachis carbonarius* (= *Pachybrachis nigricornis carbonarius*), *Pachybrachis femoratus* (= *Pachybrachis luridus*), *Pachybrachis infaustus* (= *Pachybrachis atomarius*), *Pachybrachis othonus*, *Pachybrachis tridens*, *Pachybrachis trinotatus*, and *Pachybrachis viduatus* (= *Pachybrachis bivittatus*). In 1918, he published a list of corrections brought to his attention by Fall (Chagnon 1918), noting *Pachybrachis viduatus* as a wrong determination in Chagnon (1917) and Wickham (1896).

In the thirties, Chagnon published a series of contributions on the most important beetles of the province of Québec in the journal *Le Naturaliste canadien* (Chagnon 1933–1939). The Cryptocephalini were treated in 1937 (Chagnon 1937). Four species of *Pachybrachis* were keyed out (*Pachybrachis bivittatus*, *Pachybrachis carbonarius* (= *Pachybrachis nigricornis*), *Pachybrachis othonus* and *Pachybrachis trinotatus*), and an additional four only mentioned (*Pachybrachis atomarius*, *Pachybrachis femoratus* (= *Pachybrachis luridus*), *Pachybrachis peccans*, *Pachybrachis relictus* Fall). The year after, Chagnon (1940) grouped together his previous contributions and published them as a separate book. The brother Adrien Robert of the Université de Montréal updated the nomenclature used in the book of Chagnon (1940), but did not otherwise modify its contents (Chagnon and Robert 1962), except that a table was added at the end of the book, giving both the nomenclature used by Chagnon (1940) and the more recent nomenclature. As regards *Pachybrachis*, no changes were noted between the two editions.

The list of Coleoptera collected by Notman (1919) at Cochrane in northern Ontario did not include *Pachybrachis*.

Father Léopold (1934) of the entomology laboratory at the agriculture institute of Oka (QC) published a list of beetles preserved in the collection of the institution. Seven species of *Pachybrachis* were listed, five of which are still valid: *Pachybrachis atomarius*, *Pachybrachis difficilis* (= *Pachybrachis nigricornis*), *Pachybrachis hepaticus*, *Pachybrachis peccans*, and *Pachybrachis tridens*. His *Pachybrachis pubescens* (= *Pachybrachis morosus*) and *Pachybrachis vestigialis* were very likely misidentified.

Hicks (1944, 1945, 1947a, b) collected mainly in southern Ontario and provided several new province records or information on the host plants or biology of species. He reported that *Pachybrachis calcaratus*, *Pachybrachis peccans*, *Pachybrachis othonus* and *Pachybrachis relictus* were taken by sweeping the vegetation, and *Pachybrachis obsoletus* was observed on willows (*Salix* sp.).

Latendresse (1963) increased to 36 the number of known chrysomelid species from the Saguenay region on the north shore of the St. Lawrence, in Québec. A century before, Saunders (1868) had found only two species of leaf beetles, but neither collector found *Pachybrachis* species in their survey.

Balsbaugh (1973) studied the geographical variation in *Pachybrachis othonus*, recognized three subspecies, and described *Pachybrachis othonus sioux* Balsbaughas a new subspecies. The geographical variation of *Pachybrachis nigricornis* was treated three years later (Balsbaugh and Tucker 1976), but the distribution of subspecies needs to be clarified in eastern Canada since three have been reported for this region (Riley et al. 2003).

The *Checklist of Chrysomelidae of Canada, United States, Mexico, Central America and the West Indies* of Wilcox (1975) was a working draft which was pretty complete taxonomically, but lacked detailed information on the distribution of species. LeSage (1991) provided the known distribution in Alaska and in Canada by province for all chrysomelid species, including *Pachybrachis*. Laplante et al. (1991) extracted the information for Québec only, and published it as a separate checklist for this province.

Lawson (1976) described and illustrated the structural details of the egg and larval case of *Pachybrachis bivittatus*. The mature larva of the same species was sketched by Lawson (1991), and LeSage (1985) described and illustrated all its larval instars and egg. This author also treated in detail these life stages for *Pachybrachis peccans*. Eggs were distinguished by their external ornamentation, the larval instars by their size and by their head and leg chaetotaxy.

Edward G. Riley, Shawn M. Clark, R. Wills Flowers and Arthur J. Gilbert are the authors of the most recent synthesis on American Chrysomelidae (Riley et al. 2002). The reader is referred to this major work for diagnoses and keys to subfamilies, tribes and genera. The North American *Pachybrachis* species have not been assigned to subgenera as the Palaearctic species were in the recent *Catalogue of Palaearctic Coleoptera* (Schöller et al. 2010).

The *Catalogue of the Leaf Beetles of America North of Mexico* by Riley et al. (2003) was the first extensive and complete catalogue ever published on this family of beetles for the North American continent north of Mexico. Consequently, we have followed the nomenclature and classification adopted by these authors. The compiling by Clark et al. (2004) of the known host plants of the Nearctic *Pachybrachis* is the best and most extensive source of information available on the subject. Both works are essential tools to anybody interested in *Pachybrachis* species and Nearctic leaf beetles in general.

An examination of leaf beetle specimens in the largest beetle collections in Kentucky, inventory work in state nature preserves and other protected areas, and a review of the literature revealed 20 species of *Pachybrachis* present in Kentucky, 14 of which were new state records (Barney et al. 2011). Twenty species of *Pachybrachis* were also reported for Illinois (Barney 1984).

The latest contribution on the eastern Canadian *Pachybrachis* is by Webster et al. (2012), based on extensive collecting of beetles in New Brunswick by the senior author. *Pachybrachis bivittatus* and *Pachybrachis m-nigrum* were added to the previously known *Pachybrachis peccans* and *Pachybrachis pectoralis* for this province.

## Material and methods

### Provinces

For the purposes of this study, eastern Canada is defined as provinces east of Manitoba: Ontario (ON), Québec (QC), New Brunswick (NB), Nova Scotia (NS), Prince Edward Island (PE), Newfoundland (NF) and Labrador (LB). When not given on labels, counties were found using the gazetteer of CPCGN (1974, 1988) for Ontario, CTQ (1987) for Québec, CPCGN (1994a) for New Brunswick, CPCGN (1993) for Nova Scotia, and CPCGN (1994b) for Prince Edward Island. No specimens were available from Labrador and Newfoundland. In older specimens, for example those collected by Brimley in Prince Edward and Hasting Counties, only the county names were given in the collection data.

### Species data

For each species of *Pachybrachis* found in eastern Canada, the following information is provided: name, synonymies, habitus photo, brief description of species recognition characters, distribution and maps, label data, recorded or potential host plants, and comments.

### Label data

For each specimen examined the following information is provided: province, county/district, date, label information that may include potential host plants, habitat or collection method, collector, number of males and females, and museum. If a specimen had a H. C. Fall identification label or was found in Fall’s personal collection (Fall-MCZ), this information was cited before the museum name. Within a species treatment, data are ordered alphabetically by province, county/district, locale, and then date.

### Sex determination

Determination of sex is relatively easy as follows: males (Figure 9a) are generally smaller and less robust than females, with the abdomen flat and more or less concave; in females (Figure 9b) the abdomen is convex beneath, the last segment with a deeply rounded fovea (depression). Singleton females of many species cannot be confidently identified without associated males for dissection.

### Size measurement

Ten males (when available) of each species were measured using a Leica Z16 APO microscope equipped with a DFC295 digital color camera and Leica Application Suite software.

There are many species of *Pachybrachis* in the eastern US and Canada that could only be confidently separated and identified via examination of the male reproductive organ (aedeagus). This was accomplished by removing the labels from a pinned (and usually pointed) specimen and placing the pointed, pinned specimen in gently boiling water for one minute. The now relaxed pinned (or separated) beetle was placed in a Petri dish with a small amount of 70% Ethanol. The beetle was held upside down with featherweight forceps and the abdomen pried off with an insect pin. The abdomen was then held by the pygidium with a pin and the aedeagus removed with fine forceps. After drying, the beetle was reattached to a new point using clear nail polish, as were the abdomen and aedeagus.

### Plant nomenclature

The scientific and popular names of plants were taken in Fernald (1950), Scoggan (1978) and Marie-Victorin (1995).

### Physiographic features

**Map 1.**
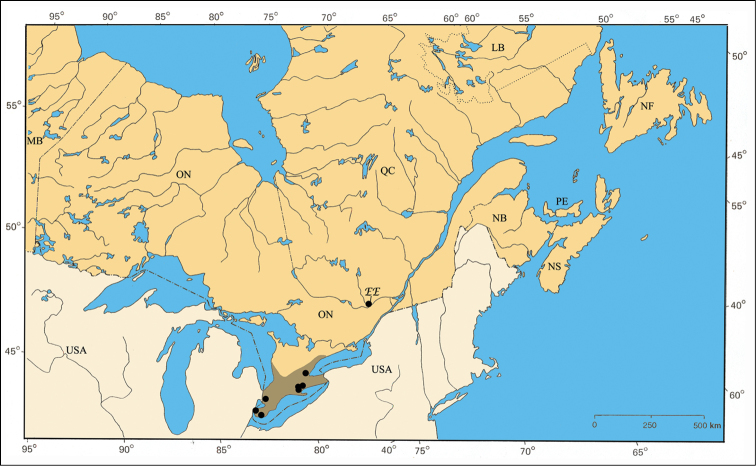
The known distribution of *Pachybrachis atomarius* in eastern Canada. Carolinian Zone in dark beige; EE – Eardley Escarpment.

The Carolinian Life Zone is by far the richest zone for the *Pachybrachis* fauna of eastern Canada, several species being exclusively associated to it (e.g. *Pachybrachis calcaratus* [Map 3], *Pachybrachis cephalicus* [Map 4], etc.). Some species are primarily present in this zone but also have, small additional northern disjunct populations (e.g. *Pachybrachis atomarius* [Map 1]). The Carolinian Zone extends into southwestern Ontario between Lakes Huron and Erie. In addition to the usual common trees of the larger Great Lakes-St. Lawrence forest region (e.g. sugar maple [*Acer saccharinum* Marsh.], beech [*Fagus grandiflora* Ehrh.], etc.), the northern limits of several deciduous trees are found in this zone: tulip-tree [*Liriodendron tulipifera* L.], pawpaw [*Asimina triloba* (L.) Dunal], black walnut [*Juglans nigra* L.], etc. (Fox and Soper 1952, 1953, 1954; Shelford 1963; Hosie 1979; Johnson 2012).

**Map 2.**
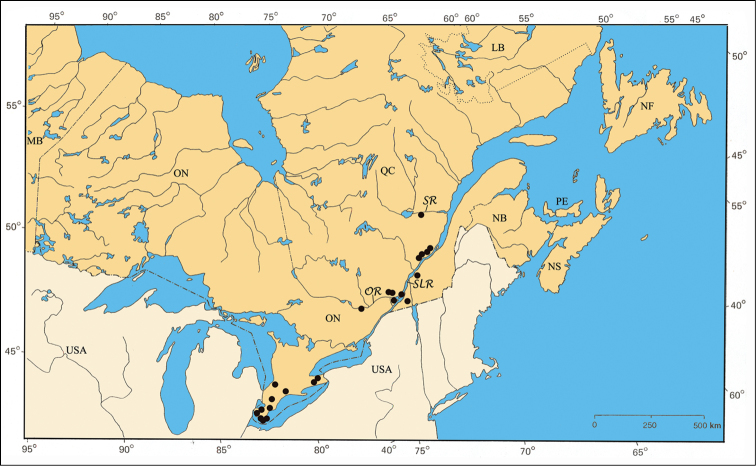
The known distribution of *Pachybrachis bivittatus* in eastern Canada. OR – Ottawa River; SLR – St. Lawrence River; SR – Saguenay River.

The major rivers seem to play an important role in the distribution of some species. For example, *Pachybrachis bivittatus* [Map 2] is closely spread along the Great-Lakes-St. Lawrence River system and in the Ottawa River valley. The Saguenay River seems a northern limit impassable for all species.

There are two noticeable disjunct refugia. The most important is the well-known Eardley Escarpment located on the south border of Gatineau Park. It corresponds to about 40 km of steep cliffs oriented southwards which are significantly warmer than the Ottawa Valley below and the Laurentian Highlands above. Eastern red cedar (*Juniperus virginiana* L.) is well represented on the cliffs, and the relatively recent discovery of its associated olive hairstreak, *Callophrys grynea* (Hubner), has been an enthusiastically received discovery for butterfly collectors (Hall 1991, NCC 2011a). There are over thirty additional vascular plant species growing exclusively there at their northernmost limits, considerably disjunct from their main distribution south of the Great Lakes (Brunton and Lafontaine 1971).

The second refugium corresponds to a small zone between the islands Île-aux-Allumettes and Île-du-Grand-Calumet, both in Pontiac County (QC), within the Ottawa River. A special flora has been identified there by botanists, but no results were published. On the other hand, Desroches and Laparé (2004) reported the first captures of the ribbonsnake (*Thamnophis sauritus septentrionalis* Rossman) in this refugium. The distribution of *Pachybrachis luctuosus* corresponds to this pattern [Map 6].

Finally, the influence of Lake Champlain cannot be ignored. Although almost completely lying in the states of Vermont and New York in the United States, it extends about 10 km across the Canadian border. Its microclimate is important enough to allow some plants and animals to cross the border and reach their northernmost limits in the southeastern townships of Québec (DECNY 2012). The very recent discovery of two sycamores (*Platanus occidentalis* L.) in this region - a first record for Québec - is a good exemple of such distribution (Bibeau-Lemieux 2010, 2011).

### Codens of collections examined and referred to in this study are as follow:

The major insect collections (and curators) in eastern Canada and the United States, which contained *Pachybrachis* specimens from eastern Canada, are listed below:

AJGC Art J. Gilbert Collection (private), Fresno, CA

AMNH American Museum of Natural History, New York, NY (Lee Herman)

CDFA California Department of Food and Agriculture, Sacramento, CA (Chuck Bellamy)

CEUM Collection entomologique de l’Université de Montréal, QC (Louise Cloutier)

CFIM Collection en Fiducie de l’Insectarium de Montréal, QC (Stéphane LeTirant)

CNC Agriculture and Agri-Food Canada, Ottawa, ON (Laurent LeSage)

FALL H. C. Fall Collection, Harvard Museum of Comparative Zoology, Cambridge, MA (Phillip Perkins)

FSCA Florida State Collection of Arthropods, Gainesville, FL (Paul Skelley)

LEM Lyman Entomological Museum, McGill, QC (Stéphanie Boucher)

LFC Laurentian Forestry Center (Insectarium René-Martineau) Québec, QC (Jan Klimaszewski)

LSAM Louisiana State Arthropod Museum, Baton Rouge, LA (Victoria Bayless)

MCZ Harvard Museum of Comparative Zoology, Cambridge, MA (Phillip Perkins)

MSUC Michigan State University Collection, East Lansing, MI (Gary Parsons)

RWIC Reginald Webster Insect Collection (private), Charters Settlement, NB

OSUC Ohio State University Collection, Columbus, OH (Creighton Freeman)

UNHM University of New Hampshire, Durham, NH (Donald Chandler)

USNM National Museum of Natural History, Washington D.C. (Alex Konstantinov)

## Results

### Pachybrachis

Chevrolat, 1836

http://species-id.net/wiki/Pachybrachis

Pachybrachis Chevrolat *in* Dejean, 1836: 420. Type species: *Cryptocephalus hieroglyphicus* Laicharting, 1781, by subsequent designation of Jacoby 1908: 265.
Pachybrachys : Mannerheim 1843: 311. [incorrect subsequent spelling].

#### Remarks.

There has been some debate as to the correct spelling of the genus *Pachybrachis*. Fall’s (1915) monumental work used *Pachybrachys* Chevrolat and cited its general American usage by J. L. LeConte. However, this emendation was unjustified under the rules of the International Code of Zoological Nomenclature (ICZN 1999, Article 32).

*Pachybrachis* is a member of the subfamily Cryptocephalinae Gyllenhall, 1813, commonly known as the case bearers due to the fact that all known larval stages live in a case constructed of their fecal matter and often plant debris (LeSage 1985). Their cylindrical, compact body characterizes the adults, which usually have the head retracted into the pronotum to the level of the eyes.

In the recent revision of family-group names in Coleoptera (Bouchard et al. 2011), the former tribe Pachybrachini Chapuis, 1874 was relegated to subtribe under the tribe Cryptocephalini Gyllenhal, 1813. Pachybrachina Chapuis, 1874 contains only two genera north of Mexico, *Griburius* and *Pachybrachis*, and is characterized by long filiform antennae, with a marginal bead at the base of pronotum which is not crenulate. Riley et al. (2002) separated the two genera by prosternal charateristics (prosternum broad, as wide as long in *Griburius*, narrower, longer than wide in *Pachybrachis*). Additional generic keys can be found in Blatchley (1910), Chagnon and Robert (1962), Downie and Arnett (1996), and Ciegler (2007).

**Useful morphological characters.** Fall (1915) provided a very detailed “Review of Structural Characters Useful in Taxonomy”, which we will not repeat here. However, there are a few key characters that will be useful to separate the seventeen Canadian species. These features will be described, detailed and illustrated, most of them being used in the identification key.

*Size*. The seventeen species can generally be divided into four size classes by average length: very small, <1.75 mm; small, >1.75 mm to 2.35 mm; medium, >2.35 mm to 2.85 mm; and large, >2.85 mm to 3.30 mm. *Pachybrachis hepaticus* is the only species in the very small category, with a mean length of 1.68 mm. *Pachybrachis m-nigrum* (2.59 mm), *Pachybrachis othonus othonus* (2.63 mm), and *Pachybrachis luridus* (2.65 mm) are in the medium category. *Pachybrachis trinotatus* (3.09 mm) and *Pachybrachis bivittatus* (3.12 mm) are the only species with males averaging over 3 mm in length. Small is the largest category, with the remaining eleven species. Mean length and width of males are reported for each species. Females are generally larger, thus accounting for the larger overall sizes reported by Fall (1915).

*Antennae*. In most species (e.g. *Pachybrachis atomarius*, Habitus 1; *Pachybrachis bivittatus*, Habitus 2), the length of antennae equals about 2/3 to 3/4 the length of the body. There are two noticeable exceptions. In *Pachybrachis hepaticus* (Habitus 5) the antennae do not exceed half of the body length, whereas in *Pachybrachis trinotatus* (Habitus 17) the antennae equal or exceed the body length.

**Figure 1.**
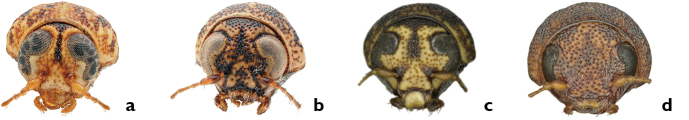
Eyes: **a** close eyes, *Pachybrachis pectoralis* **b** normal eyes, *Pachybrachis peccans* **c** normal eyes and yellow face, male *Pachybrachis atomarius* **d** remote eyes, *Pachybrachis hepaticus*.

*Eyes*. The eyes of *Pachybrachis pectoralis* are close to each other and separated by less than their width (Figure 1a). In most species the distance between the eyes roughly corresponds to their width (e.g. *Pachybrachis peccans*, Figure 1b). A normal distance between eyes, coupled with the head coloration, can be diagnostic, as in *Pachybrachis atomarius* that has a largely yellow face (Figure 1c). In *Pachybrachis hepaticus*, the eyes are very small and markedly remote, separated by much more than their diameter (Figure 1d).

**Figure 2.**
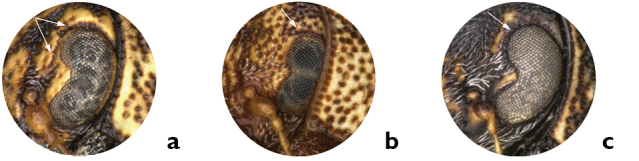
Ocular lines: **a** present, *Pachybrachis peccans* **b** small, *Pachybrachis hepaticus* **c** absent, *Pachybrachis spumarius*.

*Ocular lines*. Many *Pachybrachis* species have an impressed line, called the ocular line, around the margin of the eyes, and in some species the line diverges from each eye as lines of darker colored punctures between the eyes (e.g. *Pachybrachis peccans*, Figure 2a). This character is very consistent within each species, and it is easy to see provided the specimens are properly oriented and lighted. In *Pachybrachis hepaticus* the ocular lines are very short but distinct above the eyes (Figure 2b). In other species, however, such ocular lines are absent (e.g. *Pachybrachis spumarius*, Figure 2c).

**Figure 3.**
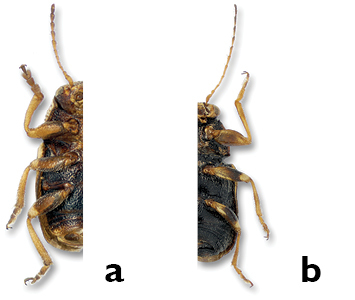
Front femora: **a** not enlarged, *Pachybrachis hepaticus* **b** thickened, *Pachybrachis peccans*.

*Femora*. Except for *Pachybrachis hepaticus* (Figure 3a), the femora on the forelegs of all species (Figure 3b) are incrassate or thickened in relation to the other femora. This character is difficult to see because in most cases legs are folded and pressed tightly against the body. Consequently, it might be necessary to relax the legs and spread them out to compare the front femora with those of the middle and hind legs. When such preparation is achieved, the larger size of the femora becomes evident (e.g. *Pachybrachis calcaratus*, Habitus 3).

**Figure 4.**
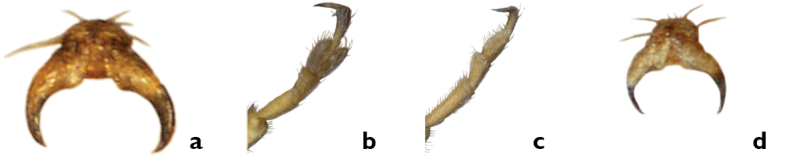
Claws: **a, b** front claws enlarged **c, d** normal.

*Claws*. In *Pachybrachis*, the tarsal claws are all simple (Figures 4a–d), but claws on the forelegs (Figures 4a, b) of several species are distinctly enlarged relative to the claws on the other legs (Figures 4c, d), as in *Pachybrachis peccans* (Habitus 12) or *Pachybrachis pectoralis* (Habitus 13). Due to the position of the legs in dead specimens, this character is often easier to see in lateral view (Figures 4c, d) than in front view (Figures 4a, b).

**Figure 5.**
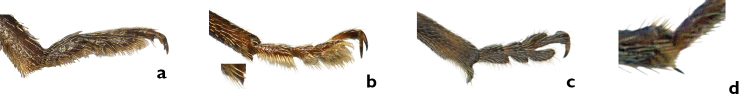
Tibial spurs: **a** absent from front leg, *Pachybrachis atomarius* **b** minute on front leg, *Pachybrachis spumarius* **c** large on front leg, *Pachybrachis calcaratus* **d** small and pointed on middle legs in most species.

*Tibial spurs*. In *Pachybrachis atomarius* (Habitus 1), *Pachybrachis m-nigrum* (Habitus 8), and *Pachybrachis trinotatus* (Habitus 17), there is no apical spur on front tibia (Figure 5a), but a tuft of large apical setae grouped together may superficially look like a spur. In *Pachybrachis spumarius* (Figure 5b, Habitus 14) the front tibial spur is very small, hidden and difficult to see, but the very large and exposed front tibial spur is unique and distinctive of *Pachybrachis calcaratus* (Figure 5c, Habitus 3). In all species, except *Pachybrachis hepaticus*, the middle tibiae are armed with small slender apical spur (Figure 5d). In all species studied here, the hind tibiae are unarmed.

**Figure 6.**
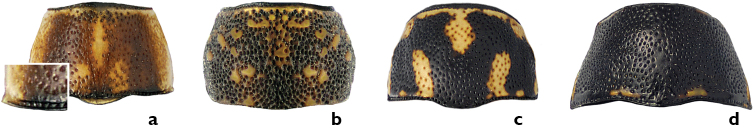
Pronotum: **a** reddish, with close-up of marginal bead, *Pachybrachis bivittatus* **b** mottled, *Pachybrachis spumarius* **c** with black M-mark, *Pachybrachis m-nigrum* **d** almost black, *Pachybrachis nigricornis carbonarius*.

*Pronotum*. In *Pachybrachis*, the pronotum is margined at base, the margin usually ornamented with a row of large punctures (Figure 6a, close up). This character is very useful to separate *Pachybrachis* Chevrolat from *Cryptocephalus* Geoffroy or *Bassareus* Haldeman. The last two genera superficially look like *Pachybrachis* but are not margined at the base of the pronotum.

The density and pattern of pronotal punctures can be a useful character. Punctures usually dissipate near the side margins, and are generally a darker color than the background.

The pronotal coloration varies from a common mottled pattern (e.g. *Pachybrachis spumarius*, Figure 6b), to a black M-mark on a light background (e.g. *Pachybrachis m-nigrum*, Figure 6c), to an almost entirely black pronotum with only yellow basal and lateral markings (e.g. *Pachybrachis nigricornis carbonarius*, Figure 6d).

**Figure 7.**
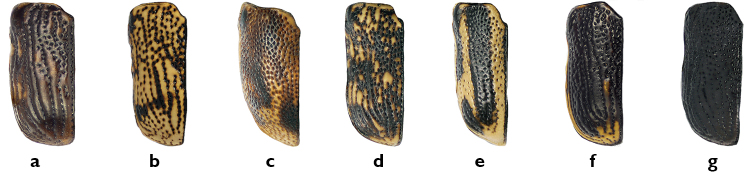
Elytral punctures and coloration: **a** in rows in deep striae, *Pachybrachis luctuosus* **b** confused in basal half, in rows in apical half, *Pachybrachis calcaratus* **c** all confused, *Pachybrachis hepaticus* **d** confused and mottled, *Pachybrachis spumarius* **e** vittate with marginal vitta interrupted, *Pachybrachis bivattatus* **f** black, margined with yellow, *Pachybrachis nigriconis carbonarius* **g** entirely black, *Pachybrachis luridus*.

*Elytra*. As on the pronotum, the density and pattern of punctures on the elytra are easily seen and useful characters. The elytral punctures generally form fairly regular deep striae, consisting of one sutural, one marginal and eight discal striae on each elytron, although the first may be somewhat irregular in the basal third (e.g. *Pachybrachis luctuosus*, Figure 7a). Punctures may be confused in the basal half but with a tendency towards regular rows in the apical half, as in *Pachybrachis calcaratus* (Figure 7b). Finally, punctures may be completely confused and not aligned at all in rows (e.g. *Pachybrachis hepaticus*, Figure 7c).

The elytral color pattern is, of course, a very useful character for the identification of many species. The mottled pattern is common (e.g. *Pachybrachis spumarius*, Figure 7d). Some species are vittate (= with longitudinal black stripes), sometimes with a lateral vitta interrupted as in *Pachybrachis bivittatus* (Figure 7e). In some species, the elytra are largely black with only a few yellow markings or with narrow apical and lateral margins (e.g. *Pachybrachis nigricornis carbonarius*, Figure 7f), or the elytra can be entirely black (e.g. *Pachybrachis luridus*, Figure 7g).

**Figure 8.**
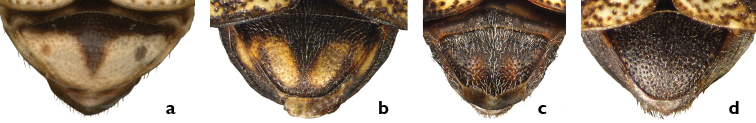
Pygidium: **a** largely yellow, *Pachybrachis bivittatus* **b** with well-defined yellow spots, *Pachybrachis cephalicus* **c** with faint reddish spots, *Pachybrachis spumarius* **d** black, *Pachybrachis atomarius*.

*Pygidium*. The coloration of the pygidium can be largely yellow (e.g. *Pachybrachis bivittatus*, Figure 8a), dark with distinct yellow spots of various sizes (e.g. *Pachybrachis cephalicus*, Figure 8b), or dark with faint small reddish spots (e.g. *Pachybrachis spumarius*, Figure 8c). A completely black pygidium is distinctive of *Pachybrachis atomarius* (Figure 8d).

**Figure 9.**
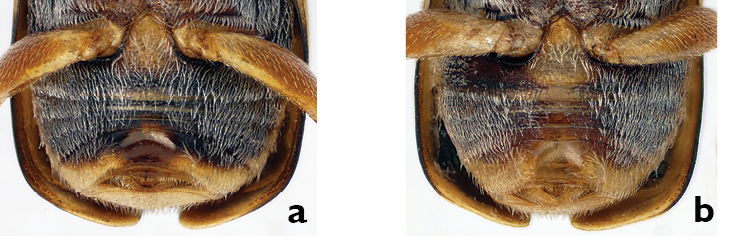
Sexes: **a** male abdomen, ventral view, *Pachybrachis bivittatus* **b** female abdomen, ventral view, *Pachybrachis bivittatus*.

*Sexes*. Males are usually smaller and less robust than females, with their abdomen flat (Figure 9a). In females, the abdomen is convex beneath, the last visible segment having a deep, round, concave depression or fovea (Figure 9b).

*Genitalia*. In most cases, individuals of each sex can be identified to species using coloration and external morphological features alone. However, an examination of the aedeagus is essential for the determination of superficially similar and variable species, such as *Pachybrachis cephalicus*, *Pachybrachis luctuosus* and *Pachybrachis spumarius*.

**Figure 10.**
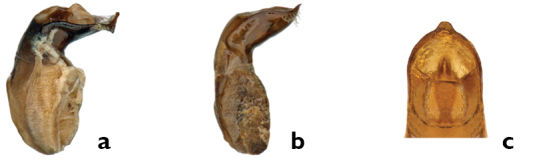
Aedeagus: **a** lateral view, *Pachybrachis luctuosus* **b** lateral view, *Pachybrachis spumarius* **c** apex, dorsal view, *Pachybrachis spumarius*.

In *Pachybrachis*, the basal portion of the aedeagus may appear bulbous (e.g. *Pachybrachis luctuosus*, Figure 10a) or more tubular (Figure 10b), but we don’t know yet if this character is reliable and consistent. The apical half is usually considerably bent, sometimes at a right angle, the degree of the curvature being an important diagnostic feature. In lateral view, the tip of the aedeagus may appear straight, sinuous and curved upwards, or sinuous and curved downwards (e.g. *Pachybrachis spumarius*, Figure 10b). In dorsal view, the tip offers various shapes: small, large, pointed, triangular, lanceolate, nipple-shaped (e.g. *Pachybrachis spumarius*, Figure 10c), etc.

Although the genitalic features are very constant and most reliable, they have been rarely described and illustrated in *Pachybrachis*. In the following key to the males of the 17 species treated here, the aedeagus is reported for only three species when external morphological characters may not be sufficient. The female genitalia are still unknown for all of them.

##### Illustrated key to males

**Table:** 

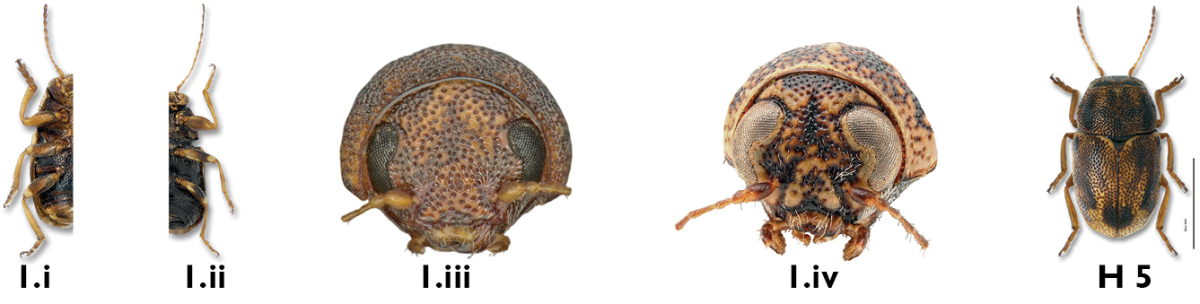		
1a	Front femora not thicker than others (Fig. 1.i); eyes small and remote (Fig. 1.iii); punctures of pronotum and elytra dense and confused (Habitus 5)	*Pachybrachis hepaticus hepaticus* (F. E. Melsheimer)
1b	Front femora thicker than others (Fig. 1.ii); eyes narrowly separated or normal (Fig. 1.iv)	2
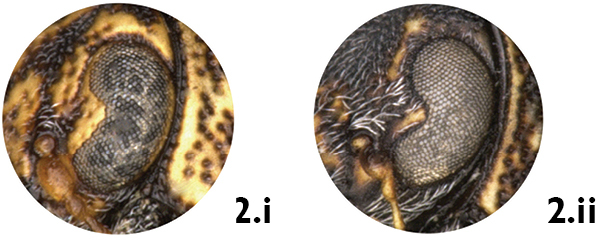		
2a	Ocular lines between eyes present (Fig. 2.i)	3
2b	Ocular lines absent (Fig. 2.ii)	6
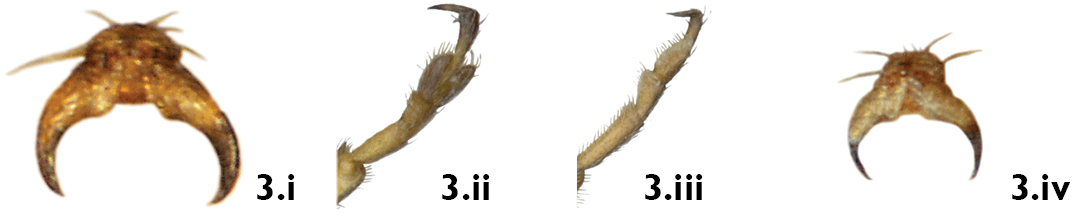		
3a	Front claws larger (Figs 3.i, 3.ii) than middle or hind claws	4
3b	Front claws not enlarged; size similar to those in middle and hind legs (Figs 3.iii, 3.iv)	5
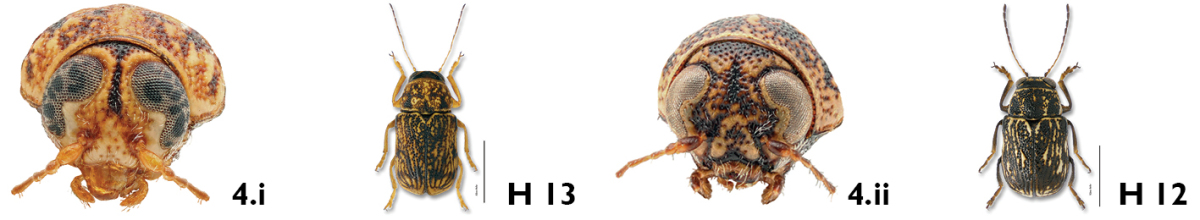		
4a	Eyes very close, separated by less than their width; ocular lines fine to indistinct (Fig. 4.i; Habitus 13)	*Pachybrachis pectoralis* (F. E. Melsheimer)
4b	Eyes normal, more distant, ocular lines very distinct, with darker punctures (Fig. 4.ii; Habitus 12)	*Pachybrachis peccans* Suffrian
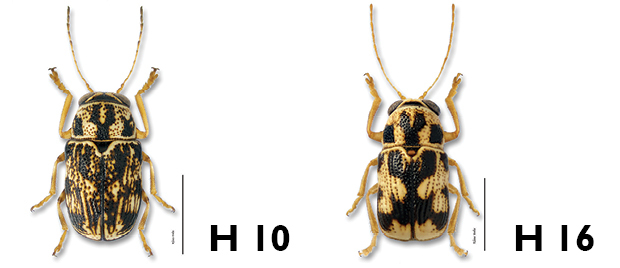		
5a	Last antennomere brownish; pronotum with subrectangular black markings pierced with yellow spots; elytral dark markings diffuse and irregular (Habitus 10)	*Pachybrachis obsoletus* Suffrian
5b	Antennae entirely yellow; pronotum with solid black rectangular markings; elytral dark markings well-defined (Habitus 16)	*Pachybrachis tridens* (F. E. Melsheimer)
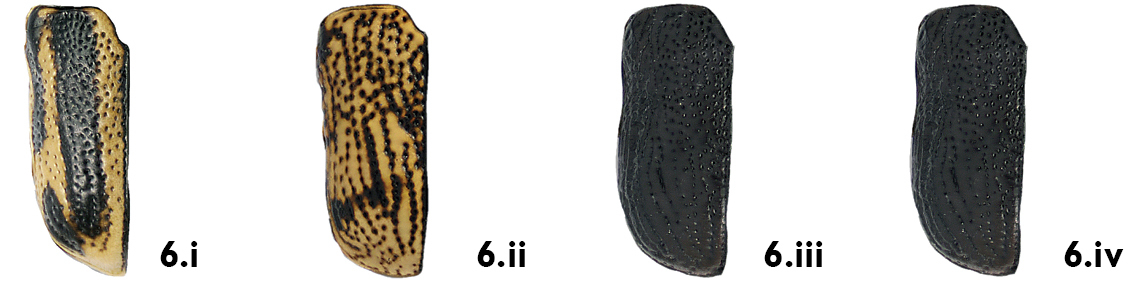		
6a	Elytra vittate (with longitudinal color stripes) (Fig. 6.i)	7
6b	Elytra mottled (Fig. 6.ii), spotted (Fig. 16.iii), or mostly to entirely black (Fig. 6.iv)	9
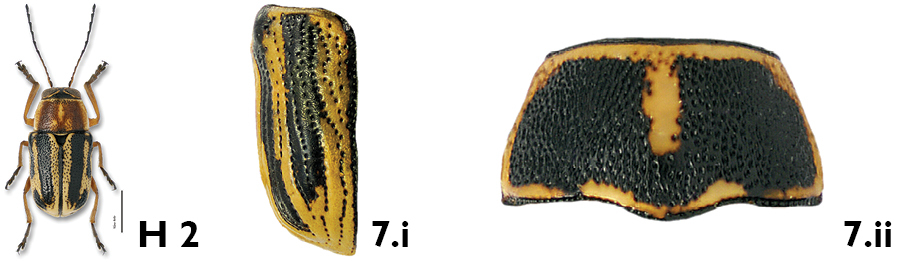		
7a	Elytral punctures confused, outer (marginal) vitta often interrupted, pronotum yellow with darker reddish M-shaped mark (Habitus 2)	*Pachybrachis bivittatus* (Say)
7b	Many elytral punctures arranged in rows (Fig. 7.i); pronotum not reddish, rather yellow with black markings of various sizes and shapes (Fig. 7.ii)	8
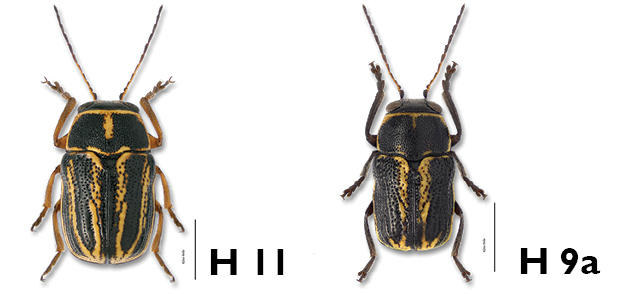		
8a	Pronotum black, margined with yellow; sutural, discal and marginal vittae of elytra distinct (Habitus 11)	*Pachybrachis othonus othonus* (Say)
8b	Pronotum yellow with large, black, M-shaped marking; marginal and discal vittae of elytra very variable, usually not distinct (Habitus 9a)	*Pachybrachis nigricornis difficilis* Fall
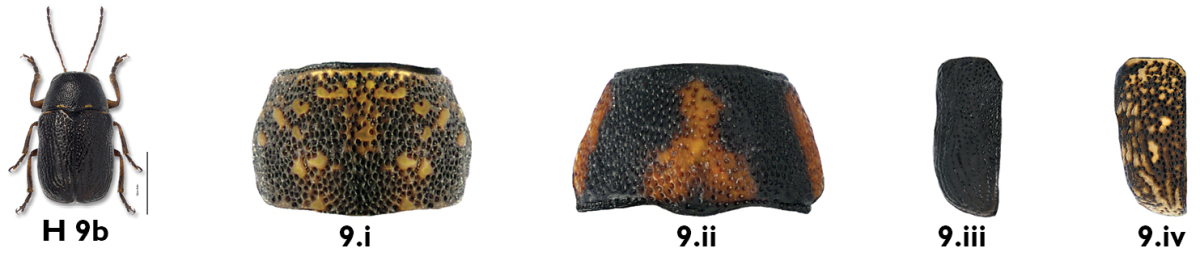		
9a	Body entirely black, or sides and apex narrowly margined with yellow in some females (Habitus 9b)	*Pachybrachis nigricornis carbonarius* Haldeman
9b	Pronotum mottled (Fig. 9.i) or with reddish spots (Fig. 9.ii); elytra variable, black (Fig. 9.iii) to mottled (Fig. 9.iv)	10
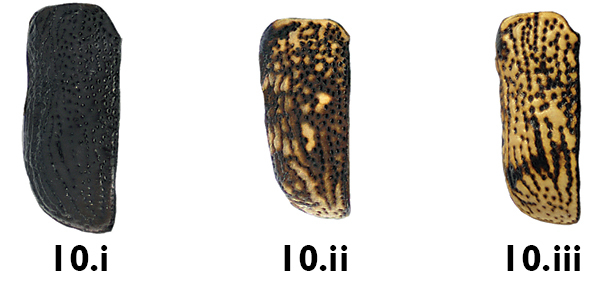		
10a	Elytra almost or entirely black (Fig. 10.i)	11
10b	Elytra spotted (Fig. 10.ii) or mottled (Fig. 10.iii)	13
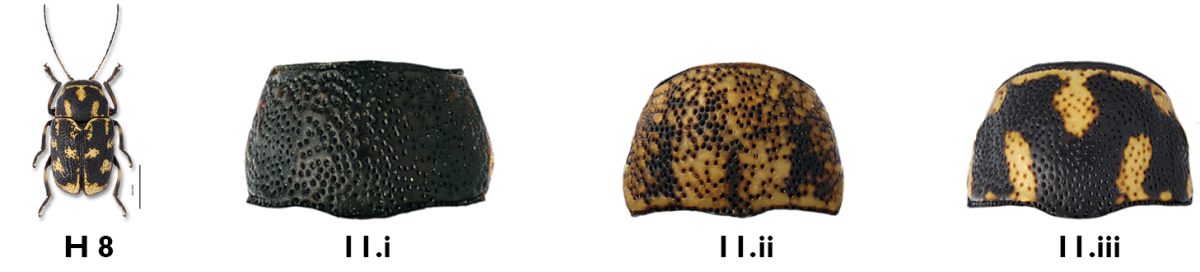		
11a	Antennae longer than body; pronotum reddish with black M-mark; elytra entirely black (Habitus 17)	*Pachybrachis trinotatus* (F. E. Melsheimer)
11b	Antennae shorter than body; pronotum black (Fig. 11.i), mottled (Fig.11.ii), or with M-shaped marking (Fig. 11.iii, H 8 below); if pronotum with M-shaped marking, then elytra mottled, not entirely black	12
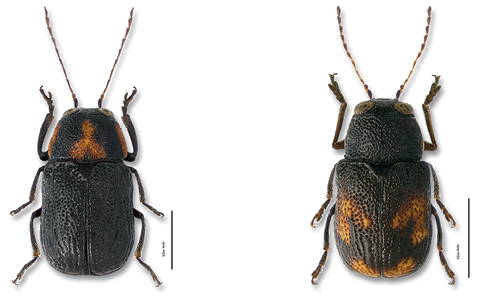		
12a	Elytra entirely black to streaked with whitish-yellow in outer areas; pronotum with reddish sides and upside-down reddish Y-mark (Habitus 7)	*Pachybrachis luridus* (Fabricius)
12b	Elytra with large median reddish spots almost joining at suture, with additional smaller apical spots; pronotum entirely black (Habitus 15)	*Pachybrachis subfasciatus* (J. E. LeConte)
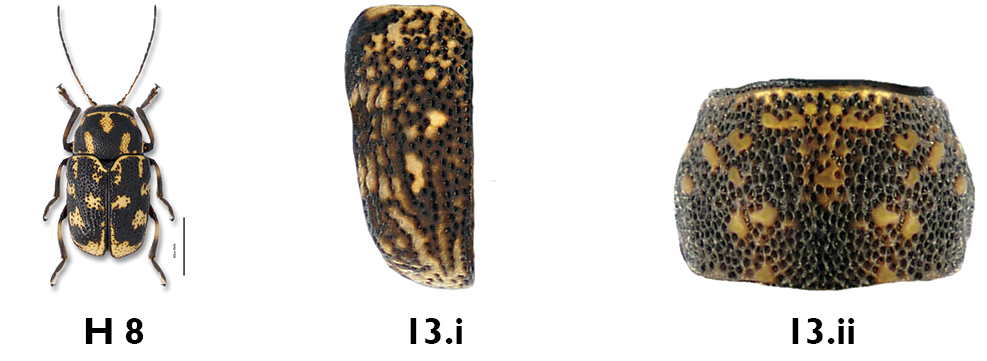		
13a	Elytra yellow with black markings; pronotum with well-defined black M-mark; size larger, 3+ mm (Habitus 8)	*Pachybrachis m-nigrum* (F. E. Melsheimer)
13b	Elytra (Fig. 13.i) and pronotum mottled (Fig. 13.ii), no decernable pattern; size smaller, less than 3 mm	14
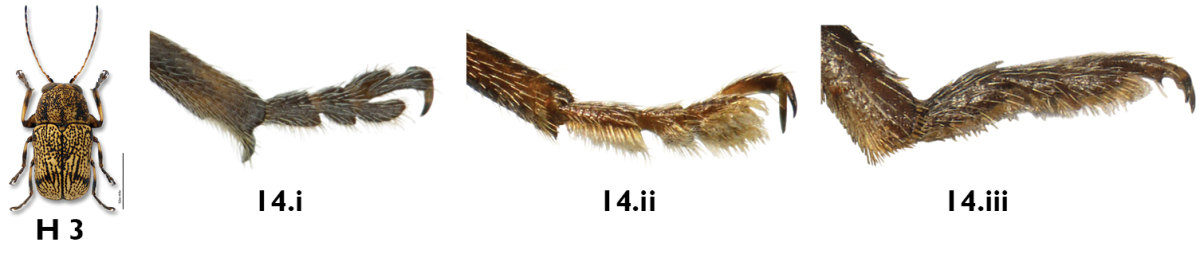		
14a	Front tibia with large, curved spur (Fig. 14.i, Habitus 3)	*Pachybrachis calcaratus* Fall
14b	Front tibia with tiny spur (Fig. 14.ii), or without spur (Fig. 14.iii)	15
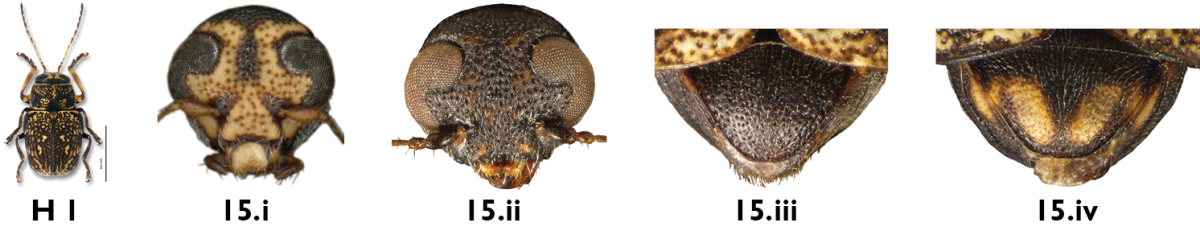		
15a	Face largely yellow in males (Fig. 15.i); pygidium entirely black (Fig. 15.iii); pronotum and elytra mottled (Habitus 1)	*Pachybrachis atomarius* (F. E. Melsheimer)
15b	Face largely dark (Fig. 15.ii); pygidium spotted, with spots of some specimens being smaller and fainter than illustrated (Fig. 15.iv)	16
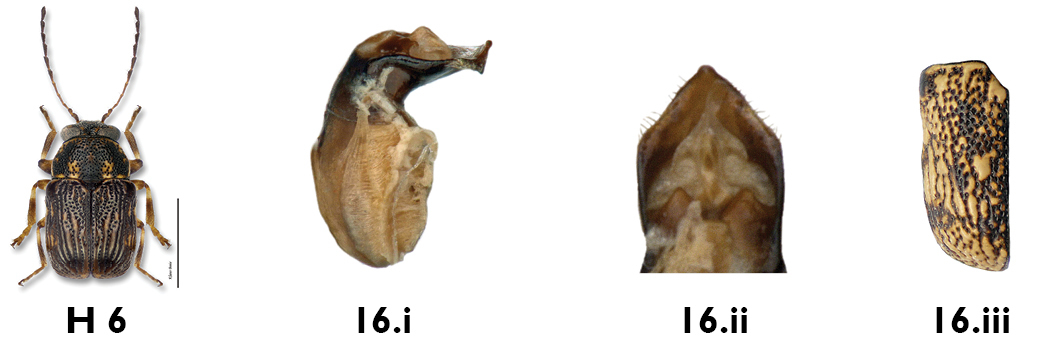		
16a	Elytral punctures regular in deeply impressed striae; size small (2 mm) (Habitus 6); aedeagus with apical diamond-shaped denticle (Figs 16.i, 16.ii)	*Pachybrachis luctuosus* Suffrian
16b	Elytral punctures confused, not inserted into deep striae (Fig. 16.iii); size larger (2+ mm); aedeagus different (Figs. 17.i, 17.iii)	17
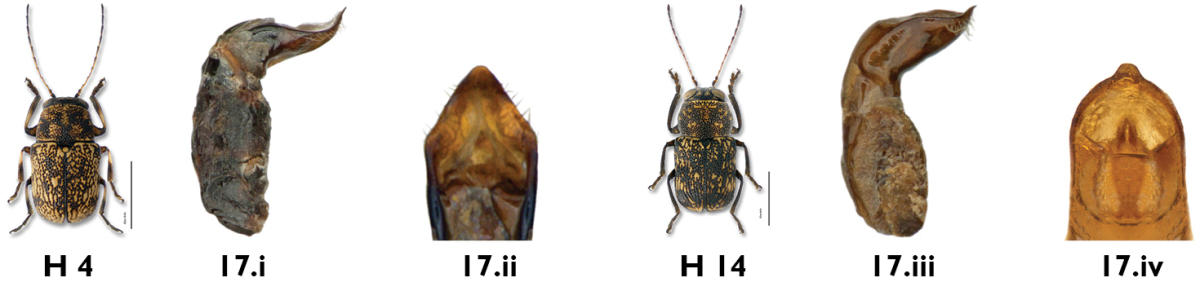		
17a	Pronotum mostly fuscous, with relatively few pale markings, densely punctate, darker than elytra (Habitus 4); aedeagus slender, sinuous, and sharper at apex in lateral view (Fig. 17.i); aedeagal tip triangular in dorsal view (Fig. 17.ii)	*Pachybrachis cephalicus* Fall
17b	Markings of pronotum and elytra numerous; darker areas dark brown to rufous (Habitus 14); aedeagus sinuous and thicker at apex in lateral view (Fig. 17.iii); aedeagal tip nipple-shaped in dorsal view (Figs. 17.iv)	*Pachybrachis spumarius* Suffrian

### Pachybrachis atomarius

(F. E. Melsheimer, 1847)

http://species-id.net/wiki/Pachybrachis_atomarius

Habitus 1 ; Map 1 ; Figures 1c , 5a , 8d

Cryptocephalus atomarius F. E. Melsheimer, 1847: 170.
Pachybrachis infaustus Haldeman, 1849: 262.
Pachybrachys atomus Bowditch, 1909: 319.

#### Recognition.

Body largely fuscous, dull, mottled with many yellow spots (Habitus 1); elytral puncturation dense, confused discally, more or less arranged in rows towards rear and sides; face of males predominately yellow; pygidium entirely black, convex (Figure 8d); male size small: length 1.85 ± 0.07 mm, width 1.01 ± 0.03 mm.

#### Distribution.

The distribution in eastern Canada is restricted in southern Ontario to remnants of the Carolinian forest (Johnson 2012; Shelford 1963). In Québec, the distribution is isolated from the main distribution area (Map 1). The unique specimen available was probably collected on the Eardley Escarpment, which is a warmer refugium created by cliffs of the Laurentian Highlands oriented southwards (Brunton and Lafontaine 1971).

#### Material examined.

ONTARIO: Essex Co., Ojibway, 7.VIII.1943, S. D. Hicks [6♀, CNC]; same data, except 8.VII.1943, S. D. Hicks [1♀, CNC]; Point Pelee, 9.VII.1931, W. J. Brown [5♂ 1♀, CNC]; Roseland, 24.VI.1944, S. D. Hicks [1♂, CNC]; Lambton Co., Walpole Island [1♂ 1♀, CNC]; Norfolk Co., Normandale, 4.VI.1931, W. J. Brown [1♂ 1♀, CNC]; Turkey Point, 8.VII.1931, W. J. Brown [1♂ 4♀, CNC]; Walsh, 10.VI.1931, W. J. Brown [1♀, CNC]; Ontario Co., Fisher Glen, 12.VI.1931, W. J. Brown [2♂ 6♀, CNC].

QUÉBEC: Pontiac Co., Old Chelsea, 12.VII.1961, J. R. Vockeroth [1♂, CNC].

#### Host plants.

No plant association records were available from specimens examined, and Barney et al. (2011) did not report any either. Clark et al. (2004) presents the known literature, but since adults were usually swept from vegetation, these records cannot automatically be interpreted as real host associations.

#### Comments.

*Pachybrachis atomarius* is one of Fall’s (1915) Group C species that have “great variation in the degree of (elytral) maculation.” In spite of the extremely variable elytral mottling, ranging from heavily speckled with yellow to almost entirely black, *Pachybrachis atomarius* males are relatively easy to identify by the combination of the predominately yellow face (Figure 1c) and entirely black, convex pygidium (Figure 8d). The entirely black, convex pygidium character also permits identification of singleton females.

Although *Pachybrachis atomarius* is a typical eastern North American species distributed from Manitoba to Oklahoma to Atlantic states (Riley et al. 2003; LeSage 1991; Barney, unpublished data), in Ontario it is restricted to the Carolinian Zone in the southernmost part of the province. Its presence in Québec is considerably disjunct from its main distribution area, and this isolation is due to the warmer microhabitat of the Laurentian Highlands cliffs of Eardley Escarpment, which are fully exposed southwards and harbor similarly disjunct insects and plants (Hall 1991; Layberry et al. 1998; NCC 2011a).

Since *Pachybrachis atomarius* has not been collected in eastern Canada over the last 50 years, it is likely extirpated from the eastern Canadian fauna.

### Pachybrachis bivittatus

(Say, 1824)

http://species-id.net/wiki/Pachybrachis_bivittatus

Habitus 2 ; Map 2 ; Figures 6a , 7e , 8a , 9a, 9b

Cryptocephalus bivittatus Say, 1824: 440.
Pachybrachys albescens Suffrian, 1858: 404.

#### Recognition.

Body very large, primarily yellow (Habitus 2); prothorax suffused with rufous; elytral punctation confused, with somewhat apparent rows on disc; elytral color pattern bivittate, with outer vitta rarely entire (Figure 7e); pygidium yellow (Figure 8a); male size large: length 3.12 ± 0.16 mm, width 1.64 ± 0.05 mm. These characters allow identification of even singleton females.

#### Distribution.

*Pachybrachis bivittatus* is a transcontinental species found across Canada and the United States (LeSage 1991; Riley et al. 2003). In Ontario, it is most common in the southernmost counties. In Québec, it occurs in the Ottawa Valley and in the St. Lawrence Lowlands. The Saguenay Region is probably its northernmost limit (Map 2).

#### Material examined.

ONTARIO: Carleton Co., Constance Bay, 26.VI.1995, B. F. & J. L. Carr [1♀, CNC]; Elgin Co., New Sarum, 16.VI.1956, W. J. Brown [5♂ 5♀, CNC]; Essex Co., Amherstburg, 6.VI.1936, G. M. Stirrett [2♀, CNC]; Belle River, 26.V.1946, S. D. Hicks [1♂, CNC]; Kingsville, 23.V.1962, Kelyone & Thorpe [1♀, CNC]; same data, except 19.VI.1954, G. B. Wiggins [1♀, ROM]; Pelee Island, 24–27.VI.1935, R. C. Osburn [1♂ 1♀, OSUC]; same data, except 4.VII.1940, W. J. Brown [2♀, CNC]; Point Pelee, 29.VI.1931, W. J. Brown [4♂ 7♀, CNC]; same data, except 1.VI.1933, G. M. Stirrett [3♂, CNC]; same data, except 29.V.1955, S. D. Hicks [3♂ 4♀, CNC]; same data, except 28.VI.1961, Kelton & Brumpton [2♂ 1♀, CNC]; Haldimand Co., Dunnville, 7.VII.1961, W. & W. Plath [1♂, USNM]; same data, except 7.VIII.1961, W. Plath [1♂, USNM]; Kent Co., Thamesville, 15.VI.1930, G. M. Stirrett [1♀, CNC]; Rondeau Park, 5.VI.1985, J. M. Campbell & A. Davies [1♀, CNC]; Lambton Co., Grand Bend, 17.VI.1956, W. J. Brown [1♂ 4♀, CNC]; Lincoln Co., Beamsville, 19.VII.1939, S. D. Hicks [3♂ 3♀, CNC].

QUÉBEC: Argenteuil Co., Carillon, 24.V.1974, E. J. Kiteley [2♂ 1♀, CNC]; Deux-Montagnes Co., La Trappe, 5.VIII.1932, J. Ouellet [1♂, CEUM]; Île-de-Montréal Co., Montréal, F. Knab [1♂, USNM]; same data, except 31.V.1941 [1♀, CEUM]; same data, except 15.VI.1961, M. Larochelle [1♂, CEUM]; same data, except 3–14.VII.1969, E. J. Kiteley [4♂ 6♀, CNC]; same data, except 17.VI, J. Ouellet [1♀, USNM]; Nicolet Co., Bécancour, 24.VI.1967, J. L. Laliberté [1♂, IDM]; Portneuf Co., Neuville, 9.VII.1939, J. Filteau [1♂ 1♀, CEUM]; Sainte-Catherine, 17.VI.1953, J. C. Aubé [1♂ 7♀, LEM]; same data, except 8.VII.1956 [2♀, LEM]; Québec Co., Sainte-Foy, 17.VI.1933, V. Boulet [1♀, CEUM]; Cap-Rouge, 27.VI.1956, J. L. Laliberté [2♂ 2♀, IDM]; Saguenay Co., Rivière Deschênes, 22.VI.1966, A. Franeslier [1♀, CEUM]; Saint-Jean Co., Cantic, 25.VI.1983, on *Salix amygdaloides* Andersson, A. Larochelle [1♂ 3♀, CNC]; Saint- Maurice Co., Pointe-du-Lac, 3.VII.1937 [1♀, CEUM]; Vaudreuil Co., Rigaud, 12.VII.1908, J. Ouellet [1♂, CEUM].

#### Host plants.

*Pachybrachis bivittatus* is typically associated with willows (*Salix* spp., Salicaceae) (Fall 1915; Barney 1984; Downie and Arnett 1996; Clark et al. 2004 for literature review). In Canada, MacNay (1958) reported a light infestation of *Pachybrachis bivittatus* on the foliage of willow along the river banks north of Coaldale, in Alberta. In eastern Canada, LeSage (personal observations) observed it on Bebb’s willow (*Salix bebbiana* Sarg.), sand-bar willow (*Salix interior* Rowlee), and stalked willow (*Salix petiolaris* J.E. Smith). Larochelle (see above) collected it on the peach-leaved willow (*Salix amygdaloides* Andersson). The larvae feed in the litter, on willow leaves, but only when they are decayed (LeSage 1985).

#### Comments.

With its large size, bivittate elytra, and close association with willows, *Pachybrachis bivittatus* is one of the easiest species to identify.

### Pachybrachis calcaratus

Fall, 1915

http://species-id.net/wiki/Pachybrachis_calcaratus

**Map 3.**
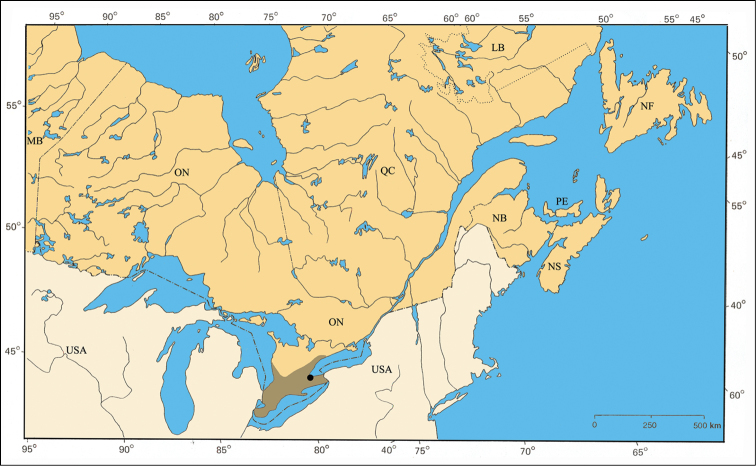
The known distribution of *Pachybrachis calcaratus* in eastern Canada. Carolinian Zone in dark beige.

Habitus 3 ; Map 3 ; Figures 5c , 7b

Pachybrachys calcaratus Fall, 1915: 389

#### Recognition.

Color dull yellow, with diffuse brown markings on pronotum, with more contrasting markings on elytra (Habitus 3); ocular lines absent; front tibiae of male with subapical rectangular tooth on inner margin, due to abrupt narrowing of tibia, and with long stout curved terminal spur (Figure 5c); terminal spur of front tibia of female reduced to narrow spine; male size small: length 2.09 ± 0.07 mm, width 1.106 ± 0.04 mm.

#### Distribution.

*Pachybrachis calcaratus* has been found across the central portion of the United States but only in small numbers (Barney, unpublished data). Within the area of this study, it is restricted to the Carolinian Life Zone in southern Ontario (Map 3).

#### Material examined.

ONTARIO: Essex Co., Roseland, 24.VI.1942, ‘compared with type’, J. A. Wilcox [1♂, CDFA]; same data, except 24.VI.1944, S. D. Hicks [1♂, CNC].

#### Host plants.

No information was recorded on the specimens collected in southern Ontario. Clark et al. (2004) reported *Pachybrachis calcaratus* nibbling purple loosestrife, *Lythrum salicaria* L. (Lythraceae), in Ohio.

#### Comments.

*Pachybrachis calcaratus* is another of Fall’s (1915) Group C species that have “great variation in the degree of (elytral) maculation.” The tibial spur (Figure 5c) is a defining character. *Pachybrachis calcaratus* was very likely associated with the Carolinian Life Zone in southern Ontario in the past. However, it has not been seen from there for the last 68 years.

### Pachybrachis cephalicus

Fall, 1915

http://species-id.net/wiki/Pachybrachis_cephalicus

**Map 4.**
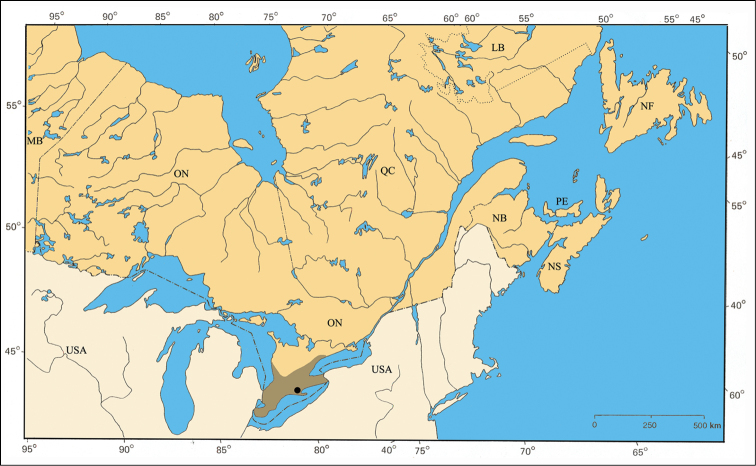
The known distribution of *Pachybrachis cephalicus* in eastern Canada. Carolinian Zone in dark beige.

Habitus 4 ; Map 4 ; Figure 8b

Pachybrachys cephalicus Fall, 1915: 419.
Pachybrachys cephalicus var. *Pachybrachis dixianus* Fall, 1915: 419.
Pachybrachys cephalicus var. *Pachybrachis parvus* Fall, 1915: 419.

#### Recognition.

Pronotum and head generally fuscous, densely punctate and darker than elytra; elytra with puncturation dense and confused (Habitus 4); ocular lines absent; male size small: length 1.94 ± 0.12 mm, width 1.05 ± 0.08 mm.

#### Distribution.

A typical eastern species distributed from Louisiana to New York to Atlantic Coast (Riley et al. 2003), restricted to southern Ontario in eastern Canada (Map 4).

#### Material examined.

ONTARIO: Norfolk Co., Walsingham Forest Station, 28.VII.1982, ex. *Potentilla* or strawberry, L. LeSage [11♂ 14♀, CNC].

#### Host plants.

Cinquefoil (*Potentilla* sp.) and strawberry (*Fragaria* sp.) (both Rosaceae) are the first host associations reported for *Pachybrachis cephalicus*. The specimens were swept from these two plants growing in a sandy clearing within a dry oak-pine forest (LeSage, personal field notes). Since 1984, the previous Walsingham Forestry Station is part of the St. Williams Dwarf Oak Forest, the largest block of publicly owned forest in the Carolinian Life Zone (NHIC 1998).

#### Comments.

*Pachybrachis cephalicus* is another of Fall’s (1915) Group C species that have “great variation in the degree of (elytral) maculation.” The fairly large number of examined specimens may be misleading since they all come from only one event. In fact, *Pachybrachis cephalicus* is very rarely collected in eastern Canada and known from only one locality within the Carolinian Life Zone. This is also a first record of this species for Canada.

### Pachybrachis hepaticus hepaticus

(F. E. Melsheimer, 1847)

http://species-id.net/wiki/Pachybrachis_hepaticus_hepaticus

**Map 5.**
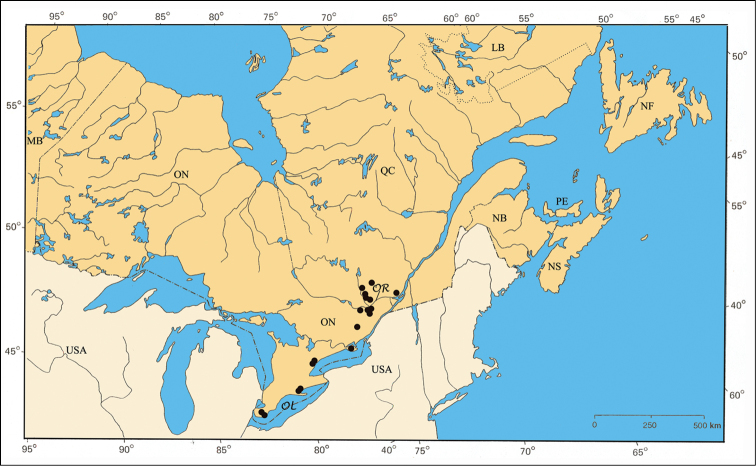
The known distribution of *Pachybrachis hepaticus hepaticus* in eastern Canada. OL – Ontario Lake; OR – Ottawa River.

Habitus 5 ; Map 5 ; Figures 1c , 3a , 7c

Cryptocephalus hepaticus F. E. Melsheimer, 1847: 171.
Cryptocephalus punctatus Haldeman, 1849: 257.

#### Recognition.

Front femora not enlarged in comparison to those of middle and hind legs (Figure 3a); eyes small and remote (Figure 1d); antennae short, less than half body length (Habitus 5); integument densely, diffusely punctate (Figure 7c); elytra tapered to apex; male size very small: length 1.68 ± 0.07 mm, width 0.96 ± 0.07 mm.

#### Distribution.

Transcontinental, extending from California to Maine (Riley et al. 2003). In eastern Canada, *Pachybrachis hepaticus hepaticus* occurs in southern Ontario from Lake Ontario to the Ottawa River Valley in Québec (Map 5).

#### Material examined.

ONTARIO: Carleton Co., Jockevale, 4.VII.1934, W. J. Brown [1♂, CNC]; Ottawa, 24.VI.1995, [ex. field notes: “pinery forest preserve, on sand dunes…”], B. F. & J. L. Carr [1♀, CNC]; Essex Co., Leamington, 3.VII.1931, W. J. Brown [1♀, CNC]; same data, except 17.VI.1940 [1♂, CNC]; Point Pelee, 24.VI.1931, W. J. Brown [1♂, CNC]; Haldimand-Norfolk Cos., Turkey Point, 24.VII.1984, sweeping in marshy area, L. LeSage [1♀, CNC]; Hasting Co., 10.VII.1938, Brimley [1♀, CNC]; Norfolk Co., Forestville, 15.VI.1931, W. J. Brown [1♂, CNC]; Parry Sound Dist., Scotia Junction, 28.VII.1934, H. W. Wenzel [1♂ 1♀, OSUC]; Prince Edward Co., 22.VI.1919, Brimley [1♀, CNC]; same data, except 28.VI.1921 [1♂, CNC]; same data, except 2.VIII.1925 [1♀, CNC]; Renfrew Co., Arnprior, 20.VII.1941, W. J. Brown [1♀, CNC]; Russell Co., Mer Bleue, 18.VI.1986, W. J. Brown [1♂, CNC]; same data, except 10.VII.1936 [1♀, CNC]; Toronto Co., Toronto, F. Knab [1♀, USNM]; same data, except 26.V.1896, R. J. Crew [1♀, ROM]. Unknown Co., East Ontario, 1885 [1♀, CNC].

QUÉBEC: Deux-Montagnes Co., La Trappe, 30.VI.1931, J. Ouellet [1♂, CEUM]; same data, except 27–29.VI.1933 [21♂ 15♀, CEUM]; same data, except 23.VII.1933 [1♀, CEUM]; same data, except 28.VII.1934 [1♀, CEUM]; same data, except 20.VIII.1936 [1♂, CEUM]; same data, except 26.VIII.1946 [1♀, CEUM]; same data, except 7.VII.1949 [1♂, CEUM]; Gatineau Co., Alcove, 24.VIII.1936, W. J. Brown [1♀, CNC]; Gatineau Park, Meach Lake, 30.VII.1972, A. Davies [1♀, CNC]; Wakefield, 20.VII.1932, W. J. Brown [2♀, CNC]; Wright, 27.VI.1933, G. S. Walley [1♂, CNC]; Labelle Co., Nominingue, 29.VII.1931, J. Ouellet [3♀, CEUM]; same data, except 6–21.VII.1932 [20♂ 13♀, CEUM]; same data, except 4.VII.1933 [2♂ 1♀, CEUM]; same data, except 2.VII.1934 [1♂, CEUM]; same data, except 8.VII.1935 [1♂, CEUM]; same data, except 22.VII.1936 [2♀, MSUC]; same data, except 2.VIII.1936 [1♂, CEUM].

#### Host plants.

No plant association records were available from specimens examined. *Pachybrachis hepaticus hepaticus* may be a polyphagous species, considering the number of plant families listed in Clark et al. (2004): Asteraceae, Euphorbiaceae, Fabaceae, Juncaceae, Salicaceae, and Tamaricaceae.

#### Comments.

Of all the species here studied, the shortest antennae (Habitus 5) and smallest eyes are found in *Pachybrachis hepaticus hepaticus*. Further investigation may require that a new genus is established for the eastern and western subspecies of *Pachybrachis hepaticus*. Fall (1915) cited specimens from Montréal, May 24 (Liebeck Coll.); Toronto (Crew); and Scotia Junction, July 27 (Wenzel), but these specimens could not be located and examined.

### Pachybrachis luctuosus

Suffrian, 1858

http://species-id.net/wiki/Pachybrachis_luctuosus

**Map 6.**
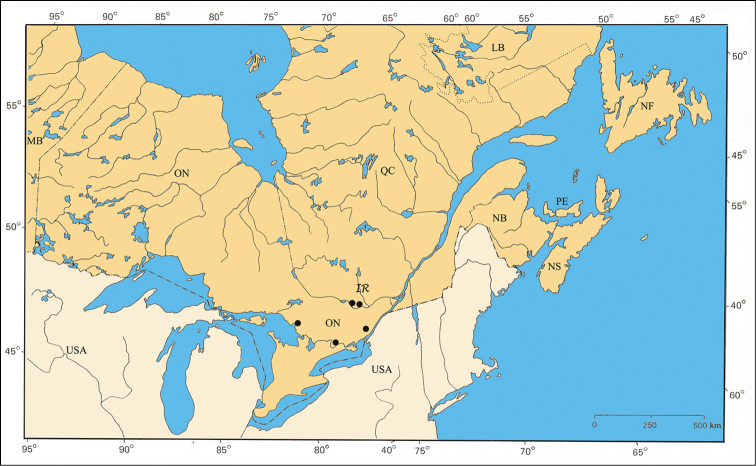
The known distribution of *Pachybrachis luctuosus* in eastern Canada. IR – Île-du-Grand-Calumet in Ottawa River.

Habitus 6 ; Map 6 ; Figures 7a , 10a

Pachybrachys luctuosus Suffrian, 1858: 401.

#### Recognition.

Color black or piceous; ocular lines absent; pronotum and sides of elytra with few yellow marks; elytral punctures confused in scutellar area, in fairly regular rows in apical half; elytral striae deep and quite regular (Habitus 6); aedeagus with terminal nodule and denticle forming small, 90o diamond shape (Figure 10a); male size small: length 1.87 ± 0.10 mm, width 0.95 ± 0.11 mm.

#### Distribution.

A relatively rare Atlantic species distributed from Alabama to New York in the United States (Riley et al. 2003; Barney, unpublished data). The Parry Sound specimens in Ontario and those of the Île-du-Grand-Calumet in the Ottawa River are two small populations disjunct from the main Atlantic one (Map 6).

#### Material examined.

ONTARIO: Hastings Co., 20.VI.1952, J. F. Brimley [1♂, CNC]; Parry Sound Dist., Hwy. 69, 12 km S Shawanaga, 13.VII.1995, B. F. & J. L. Carr [1♂, CNC]. Leeds Co., 7.VIII.1950, ex. pine, J. F. Brimley [3♂ 12♀, CNC].

QUÉBEC: Pontiac Co., L’Île-du-Grand-Calumet, 3.VIII.1985, on *Pinus resinosa* Ait., Larochelle & Larivière [1♂ 1♀, CNC]; Luskville, 4.VII.1985, on *Quercus rubra* L., Larochelle & Larivière [1♂, CNC].

#### Host plants.

A large series was taken on pine in Leeds Co., ON. *Pachybrachis luctuosus* was first reported from *Pinus virgiana* P. Mill. in Alabama (Balsbaugh and Hays 1972). This record was extended to the northeastern states by Wilcox (1979), and assumed to be valid as well in West Virginia (Clark 2000). Specimens from Larochelle & Larivière had label notations reporting collections from *Pinus resinosa* Ait. and *Quercus rubra* L.

#### Comments.

*Pachybrachis luctuosus* is another of Fall’s (1915) Group C species that have “great variation in the degree of (elytral) maculation.” Fall commented that he would not be surprised if *Pachybrachis carolinensis* Fallwas a paler form of *Pachybrachis luctuosus*. Our comparison of aedeagi of specimens identified by Fall as either *Pachybrachis luctuosus* or *Pachybrachis carolinensis* revealed the same, distinctive form – the subplanar surface with one median subapical denticle. *Pachybrachis carolinensis* appears to be a larger, more yellow variation, but more work needs to be done. *Pachybrachis luctuosus* is reported for the first time in Canada, and therefore, is also a first record for ON and QC.

### Pachybrachis luridus

(Fabricius, 1798)

http://species-id.net/wiki/Pachybrachis_luridus

**Map 7.**
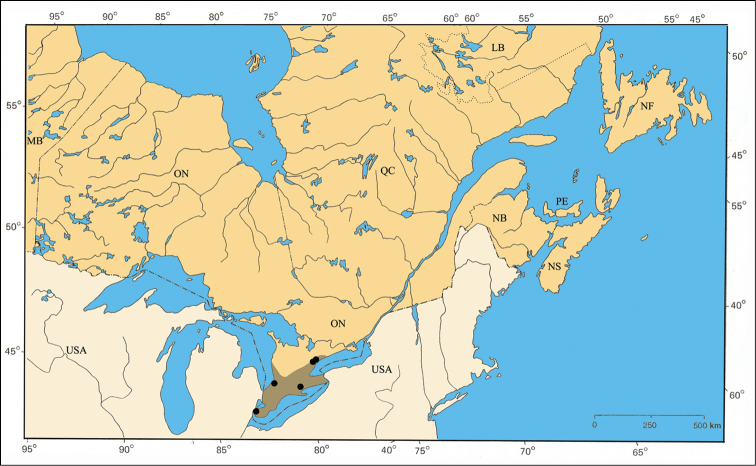
The known distribution of *Pachybrachis luridus* in eastern Canada. Carolinian Zone in dark beige.

Habitus 7 ; Map 7 ; Figure 7g

Cryptocephalus luridus Fabricius, 1798: 109.
Cryptocephalus femoratus Say, 1824: 439.
Cryptocephalus aesculi F. E. Melsheimer, 1847: 171.
Pachybrachys moerens Stål, 1857: 63.
Pachybrachys luridus var. *Pachybrachis nigrinus* Blatchley, 1910: 1130.
Pachybrachys luridus var. *Pachybrachis festivus* Fall, 1915: 470.

#### Recognition.

Body dull black, densely, coarsely punctured; pronotum black with anterior median line and sides red or reddish yellow, varying to almost entirely red; elytra mottled with yellow, especially toward sides, varying to entirely yellow to entirely black (Habitus 7); front claws of male much enlarged (as in Figure 4a); male size medium: length 2.65 ± 0.23 mm, width 1.45 ± 0.12 mm.

#### Distribution.

Occuring in the eastern half of the United States (Riley et al. 2003) to the Rocky Mountains, but in Canada restricted to the Carolinian Life Zone of southern Ontario (Map 7).

#### Material examined.

ONTARIO: Essex Co., Ojibway, 9.VI.1943, S. D. Hicks [1♀, CNC]; Lambton Co., Grand Bend, 20.VII.1930, G. E. Shewell [1♀, CNC]; Simcoe Co., 19.VI.1939, G. S. Walley [1♀, CNC]; Toronto Co., Toronto, 26.VI.1896, C. T. Hills [2♂ 2♀, LEM]; same data, except 15–30.VI.1927, L. J. Milne [1♀, UNHC]; same data, except F. Knab [29♂ 20♀, USNM]; Toronto, High Park, 4.VI.1897 [3♂ 2♀, ROM]; Unknown Co., Black Creek, 14.VI.1897 [1♀, ROM]; Springfield [2♀, ROM]; Can., G. M. Greene [1♂, USNM].

#### Host plants.

No plant association records were available from Canadian specimens. In the United States, the false indigos (*Baptisia leucantha* T. & G., *Baptisia tinctoria* (L.) R. Br.) (Fabaceae) were the associations most often cited by authors (Frost 1945, details in Clark et al. 2004). However, these plants are not present in Québec (Marie-Victorin 1995), and extremely rare in southern Ontario (Scoggan 1978). Barney et al. (2011) stated that recently collected specimens in Kentucky were probably from oak (*Quercus* spp., Fagaceae).

#### Comments.

Fall (1915) observed specimens from Ontario: Toronto (Wickham). However, no specimens of *Pachybrachis luridus* have been collected from the province in the last 68 years, and one of its potential hosts (*Baptisia* spp.) were always extremely rare in southern Ontario (Scoggan 1978). Consequently, *Pachybrachis luridus* is likely extirpated from the eastern Canadian fauna.

### Pachybrachis m-nigrum

(F. E. Melsheimer, 1847)

http://species-id.net/wiki/Pachybrachis_m-nigrum

**Map 8.**
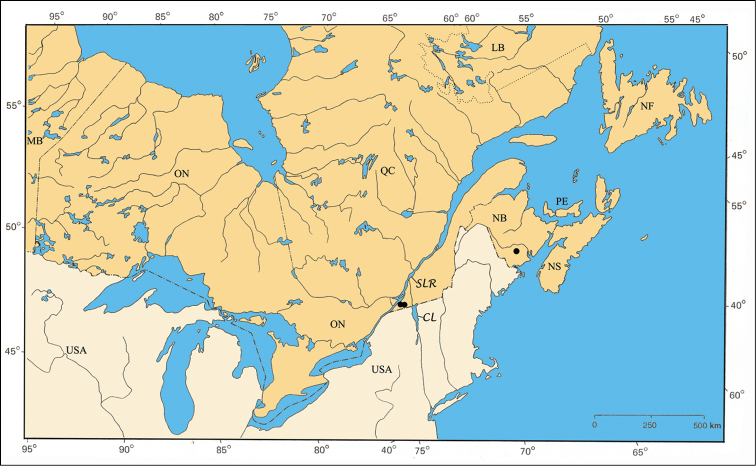
The known distribution of *Pachybrachis m-nigrum* in eastern Canada. CL – Champlain Lake; SLR – St. Lawrence River.

Habitus 8 ; Map 8 ; Figure 6c

Cryptocephalus m-nigrum F. E. Melsheimer, 1847: 170.
Pachybrachys intricatus Suffrian, 1852: 180.

#### Recognition.

Pronotum usually with thick, black, M-shaped marking; elytra yellow with variable black markings, but these usually leaving basal, lateral and sutural margins yellow, in addition to a basal and median yellow spots on each elytron (Habitus 8); male size medium: length 2.59 ± 0.11 mm, width 1.42 ± 0.07 mm.

#### Distribution.

A typical eastern species distributed in the eastern half of the United States (Riley et al. 2003). Its presence in the south of the eastern Townships, in Québec, corresponds to the northernmost limit of this species (Map 8).

#### Material examined.

NEW BRUNSWICK: York Co., 15 km W of Tracy, off Rt 645, 45.6837°N, 65.8809°W, 22.vii.2007, red pine forest, sweeping foliage of *Comptonia peregrina*, R. P. Webster [1?, RWIC].

QUÉBEC: Châteauguay Co., Ormstown, 12.VII.1977, sweeping, E. J. Kiteley [1♀, CNC]; same data, except 30.VII.1978 [1♂, CNC]; Huntingdon Co., Covey Hill, 30.VI.1927, G. S. Walley [1♀, CNC]; same data, except 1.VII.1927, W. J. Brown [1♀, CNC].

#### Host plants.

An old record by Schwarz (1890) concerned *Toxicodendron radicans* (L.) Kuntze) (Anachardiaceae), but this was probably based on misidentified specimens of *Pachybrachis tridens*. Clark et al. (2004) reviewed the literature. More recently, Webster swept it from *Comptonia peregrina* (Myricaceae) (Webster et al. 2012). Recent surveying in Kentucky barrens/prairies by Barney et al. (2011) cited *Pachybrachis m-nigrum* collected from and found to feed on St. John’s-wort, *Hypericum dolabriforme* Vent. (Clusiaceae) (Barney and Hall (2011)).

#### Comments.

Based upon external morphlogy, these specimens appear to be *Pachybrachis m-nigrum*. However, extensive dissections of material from across the eastern half of the US reveal an externally similar species, but with a distinctly different aedeagus, from the midwestern states (Barney, unpublished data).

### Pachybrachis nigricornis

(Say, 1824)

http://species-id.net/wiki/Pachybrachis_nigricornis

**Map 9.**
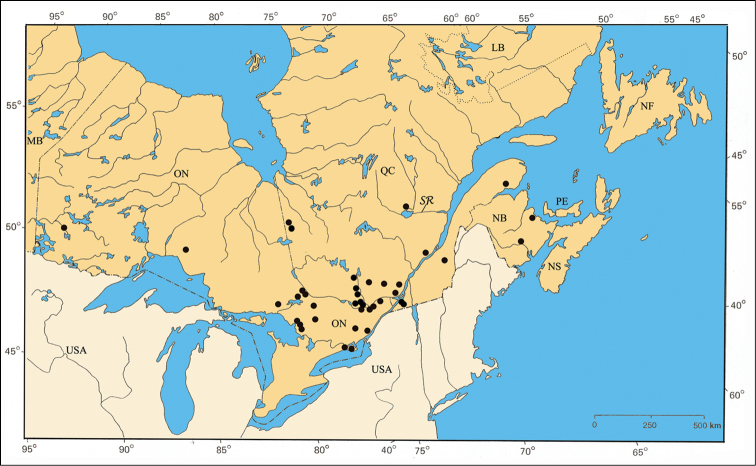
The known distribution of *Pachybrachis nigricornis* in eastern Canada. SR – Saguenay River.

Habitus 9a , 9b , 9c ; Map 9 ; Figures 6d , 7f

Cryptocephalus nigricornis Say, 1824: 436.
Pachybrachys carbonarius Haldeman, 1849: 260.
Pachybrachys autolycus Fall, 1915: 458.
Pachybrachys autolycus var. *Pachybrachis difficillis* Fall, 1915: 459.
Pachybrachys autolycus var. *Pachybrachis wahsatchensis* Fall, 1915: 459.
Pachybrachys carbonarius var. *Pachybrachis janus* Fall, 1915: 462.

#### Recognition.

Pronotum strongly alutaceous and opaque, more finely punctate than elytra. In subspecies *Pachybrachis difficilis*, black stripes usually complete and distinct (Habitus 9a), but in some specimens fused together. In subspecies *Pachybrachis carbonarius*, elytra largely black (Habitus 9b), margined with yellow, or with yellow at edge of elytra in some females (Habitus 9c); male size small: length 2.20 ± 0.14 mm, width 1.21 mm ± 0.06 mm (*Pachybrachis difficilis*); length 2.10 ± 0.07 mm, width 1.17 ± 0.04 mm (*Pachybrachis carbonarius*).

#### Distribution.

The species *Pachybrachis nigricornis* is distributed across the eastern two-thirds of North America (Riley et al. 2003). The distribution of the subspecies remains a subject of debate (Balsbaugh and Tucker 1976; Barney and Hall 2009) (Map 9).

#### Material examined.

NEW BRUNSWICK: Kent Co., Kouchibouguac National Park, 7.VII.1978, Code 7785K, H. Goulet [3♂, CNC]; same data except Code 7754F [1♂ 1♀, CNC]; same data, except 13.VII.1977, code 5599I, G.A. Calderwood [1♂ 1♀, CNC]; same data, except 4.VIII.1977, code 5779G [1♂, CNC]; same data, except 25.VIII.1977, code 5849Y, S.J. Miller [1♂ 1♀, CNC]; Queens Co., Jemseg, 18.VI.1981, ex. *Potentilla simplex*, L. LeSage & D. Ward [1♀, CNC].

ONTARIO: Carleton Co., Britannia, 14.VI.1949, R. de Ruette [2♀, CNC]; Constance Bay, 12.VII.1996, lot 3, B. F. & J. L. Carr [1♀, CNC]; Innis Point, 9–16.VII.1985, Interception trap, J. Denis & L. Dumouchel [1♂, CNC]; Ottawa, 17.VII.1912, G. Ouellet [1♀, IDM]; Hasting Co., Trenton, 28.V.2000, Evans [1♂, CNC]; Kenora Co., Berens River, 4–9.VII.1938, W. J. Brown [1♂ 1♀, CNC]; Hwy 17, 15 km east of Borups Corners, 23.VI.1996, lot 2, B. F. & J. L. Carr [1♀, CNC]; Leeds Co., Saint-Lawrence Island National Park, Grenadier Island Center, 27.V.1975, sweeping, E. Sigler [1♀, CNC]; Lennox - Addington Co. Kaladar, 21.VII.1996, lot 3, B. F. & J. L. Carr [1♂, CNC]; Muskoka Co., Bala, 24.VI.1956, W. J. Brown [3♂ 11♀, CNC]; Nipissing Co., Algonquin Provincial Park near Brent, 19.VIII.1980, R. Baranowski [1♀, CNC]; Hwy11, 30 km north of North Bay, 14.VII.1995, lot 3, B. F. & J. L. Carr [1♂ 1♀, CNC]; Hwy 17, 13 km west of Mattawa, 14.VI.1995, lot 1, B. F. & J. L. Carr [2♀, CNC]; Parry Sound Co., Parry Sound, 12.VII.1961, G. Brumpton [2♂, CNC]; same data, except 12.VII.1995, lot 1, B. F. & J. L. Carr [1♂, CNC]; Hwy 69, 12 km south Shawanaga, 13.VII.1995, Lot 3, B. F. & J. L. Carr [1♀, CNC]; Prescott Co., Alfred Bog, 8.VI.1982, breeding, ex. *Cassandra calyculata*, L. LeSage [4♂ 4♀, CNC]; same data, except 4.VI.1982, ex. Ericaceae [2♂ 4♀, CNC]; Prince Edward Co., 23.VI.1933, J. F. Brimley [1♀, CNC]; same data, except 1.VII.1936 [1♂, CNC]; same data, except 28.VI.1939 [1♂, CNC]; same data, except 19.VII.1942 [1♂, CNC]; same data, except 11–18.VI.1947 [12♂ 7♀, CNC]; same data, except 9.VI.1948 [8♂ 7♀, CNC]; same data, except 21.VI.1950 [2♀, CNC]; same data, except 17.VI.1953 [8♂ 14♀, CNC]; same data, except 19.VII.1961 [1♂, CNC]; same data, except 2.VII.2000, Evans [1♀, CNC]; Russell Co., Mer Bleue, 13.VI.1932, W. J. Brown [2♂ 1♀, CNC]; same data, except 30.VI.1932 [5♂, CNC]; same data, except 26.VII.1932 [1♂, CNC]; same data, except 30.VI.1934 [1♂, CNC]; same data, except 10.VIII.1936 [1♂, CNC]; same data, except 25.VII.1932, L. J. Milne [6♂ 3♀, CNC]; same data, except 29.VI.1954, E. C. Becker [2♂ 2♀, CNC]; same data, except 3.VII.1981, ex. *Cassandra calyculata*, L. LeSage [1♂ 1♀, CNC]; same data, except 30.VII.1979, sweeping, H. Goulet [1♂, CNC]; Sudbury Co., Sudbury, 1898 [1♀, CNC]; Thunder Bay Co., Manitouwadge, paper yard, 3.VII.1985, T. Baker [1♂ 1♀, CNC]; 59 km north junction of highways 516 & 599, 24.VI.1992, lot 1 B. F. & J. L. Carr [1♂, CNC].

QUÉBEC: Beauce Co., Beauceville, 21.VI.1937, Frère Étienne-Maurice [1♀, CEUM]; Bonaventure Co., Cascapédia, 22.VI.1933, W.J. Brown [1♂ CNC]; same data, except 9.VII.1933 [1♂, CNC]; same data, except 16.VIII.1933 [1♀, CNC]; Châteauguay Co., Ormstown, 12.VII.1977, E. J. Kiteley, sweeping [1♂, CNC]; Deux-Montagnes Co., La Trappe, VII.1933 [1♂ 1♀, *Pachybrachis autolycus*, var. *Pachybrachis difficillis* Fall, Fall-MCZ]; same data, except 25.V.1929, P. Leopold [4♀, CEUM]; same data, except 2–19.VI.1936 [1♀, CEUM]; same data, except 21.VI.1933, J. Ouellet [5♂ 3♀, CEUM]; same data, except 23.VII.1933 [1♂, CEUM]; same data, except 3–28.VII.1934 [2♂ 1♀, CEUM]; same data, except 3–17.VII.1935 [1♂ 1♀, CEUM]; same data, except 15.VI.1946 [1♂, CEUM]; Gatineau Co., Aylmer, 11.VI.1932, W. J. Brown [6♂ 4♀, CNC]; Gracefield, 22.VI.1937, O. Peck [1♂, CNC]; Kazabazua, 28.VIII.1928, W. J. Brown [1♀, CNC]; Lytton, 1.VII.1981, weeds, side of road, A. Larochelle [1♂, CNC]; Mont King, Parc de la Gatineau 7–14.VII.1997, L. LeSage & C. Lacroix [1♂ CNC]; Huntingdon Co., Saint-Antoine- Abbé, 16.VI.1983, fern, A. Larochelle [2♀, CNC]; Joliette Co., Joliette, 12.VII.1922, J. Ouellet [1♂ 2♀, CEUM]; Labelle Co., Nominingue, 21.VII.1932 [1♂, *Pachybrachis autolycus*, var. *Pachybrachis difficillis* Fall, Fall-MCZ]; same data, except 12–19.VII.1932, J. Ouellet [4♂ 3♀, CEUM]; same data, except 4.VII.1934, L. Daviault [1♀, CEUM]; Lac-Saint-Jean-Ouest Co., Mistassini, 28.VII.1944, A. Robert [1♂ 1♀, CEUM]; Montcalm Co., Parc du Mont-Tremblant, 22.VI.1956, A. Robert [1♀, CEUM]; same data, except 11.VII.1961 [1♀, CEUM]; Pontiac Co., Beech Grove, 10.VIII.1966, H. Goulet [1♂, CNC]; Portneuf Co., Sainte-Catherine, 3.VII.1956, J. C. Aubé [1♀, LEMC]; Territoires-du-Nouveau-Québec Co., Casa-Berardi, 22–29.VI.1997, interception trap, P. Paquin [2♂, LEM]; same data, except 6–27.VII.1997 [4♂ 2♀, LEM]; same data, except 3–24.VIII.1997 [3♂ 1♀, LEM]; Chemin Selbale, 6–27.VII.1997, interception trap, P. Paquin [1♂ 4♀, LEM].

#### Host plants.

Although over 200 specimens were examined, the only potential host plants recorded by collectors were *Cassandra calyculata* (L.) D. Don (Ericaceae) and *Potentilla simplex* Michx. (Rosaceae). Balsbaugh and Tucker (1976) reported that they collected series of *Pachybrachis carbonarius* on wild strawberry (*Fragaria* sp.) (Rosaceae), and tick-trefoil (*Desmodium* sp.) (Fabaceae) in Alabama. Barney and Hall (2009) reared the same subspecies on *Desmodium paniculatum* L. DC and *Lespedeza virginica* (L.) Britton (Fabaceae) in Kentucky.

#### Comments.

Balsbaugh and Tucker (1976) and Riley et al. (2003) recognized *Pachybrachis nigricornis* as having four subspecies in North America. Fall (1915) described *Pachybrachis autolycus* as a separate species with two “varieties”, and he added the variety *Pachybrachis janus* to *Pachybrachis carbonarius*, which he also recognized as a separate species. Specimens from each of the provinces of NB, ON and QC included an assortment of var. *Pachybrachis carbonarius*, var. *Pachybrachis difficilis*, and the yellow variation of *Pachybrachis carbonarius* as per Barney and Hall (2009). In the “Material examined” section above, the QC specimens from Fall’s personal collection (Fall-MCZ) are listed, with an indication of his identification label of *Pachybrachis autolycus*, var. *Pachybrachis difficillis*.

### Pachybrachis obsoletus

Suffrian, 1852

http://species-id.net/wiki/Pachybrachis_obsoletus

Habitus 10 ; Map 10

Pachybrachys obsoletus Suffrian, 1852: 200.

#### Recognition.

Background color yellow, with numerous, usually not sharply outlined black spots on both pronotum and elytra (Habitus 10); eyes distant; ocular lines faint; male size small: length 1.87 ± 0.16 mm, width 0.98 ± 0.08 mm.

#### Distribution.

**Map 10.**
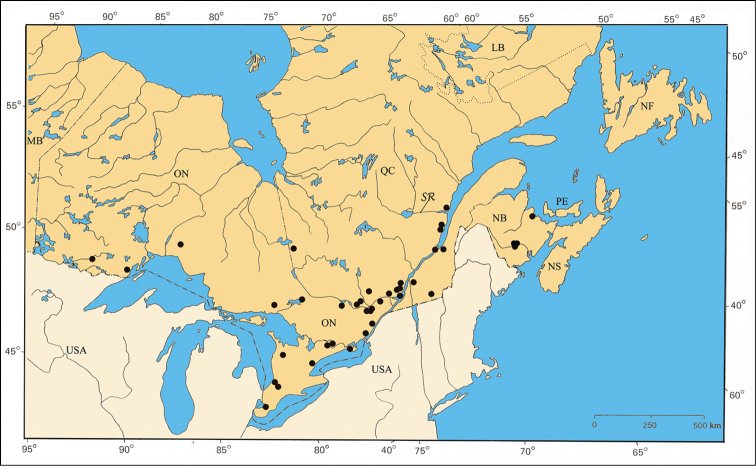
The known distribution of *Pachybrachis obsoletus* in eastern Canada. SR – Saguenay River.

Species broadly distributed from North Dakota to New Mexico to Atlantic Coast in the United States (Riley et al. 2003), and in Canada from British Columbia to New Brunswick. In eastern Canada, it is found in Ontario, Québec and New Brunswick (Map 10).

#### Material examined.

NEW BRUNSWICK: Kent Co., Kouchibouguac National Park, 21.VII.1977, Code 5680L, D. J. Brown [1♀, CNC]; same data, except 17.VII.1978, Code 7291K, D. B. Lyons [1♀, CNC]; Queens Co., Canning Grand Lake near Flowers Cove, 1.VII.2004, D. Sabine and R. Webster [2♂ 2♀, RWIC]; White’s Cove, Grand Lake, 24.VII.1957, ex. *Salix lucida* [2♂ 2♀, CNC]; Sunbury Co., 9.5 km NE Jct. 101 & 645, 22.VII.2007, R. P. Webster [1♀, RWIC]; York Co., Fredericton, French Lake, 20.VII.1931, C. W. Maxwell [1♀, LEM]; Fredericton, 22.VII.1936, R. E. Balch [1♀, CNC]; French Lake, 2.VI.1928, W. J. Brown [1♀, CNC].

ONTARIO: Carleton Co., Carp, 5.VII.1932, W. J. Brown [1♂, CNC]; Merivale, 16.VII.1936, W. J. Brown [2♀, CNC]; Stittsville, 18.VII.1963, J. F. McAlpine [1♂, CNC]; Stittsville, 18.VIII.1963, Malaise trap [1♀, CNC]; Durham Co., Durham, VI.1969, [1♀, CNC]; Hastings Co., 10.VII.1938, J. F. Brimley [2♀, CNC]; same data, except 16.VII.1950 [2♀, CNC]; same data, except 31.VI.1936 [1♀, CNC]; Marmora, 19.VI.1952, J. R. Vocheroth [1♂, CNC]; same data, except 4.VII.1952, J. R. McGillis [1♀, CNC]; same data, except 18.VIII.1952, E. H. N. Smith [1♀, CNC]; Kent Co., Tilbury, 20.VIII.1947, on willow, S. D. Hicks [1♀, CNC]; Lambton Co., Grand Bend, 11.VII.1939, G.E. Shewell [1♀, CNC]; same data except 20.VII.1939 [1♀, CNC]; Lanark Co., Bell’s Corners, 6.VI.1942, F.I. Survey 1942, Rec 5436C, White Pine [1♀, CNC]; Leeds Co., Mulcaster Island, Saint-Lawrence Island National Park, 17.VIII.1976, Sweeping *Pinus strobus*, Code 4438, W. Reid [1♀, CNC]; Thwartway Island, Saint-Lawrence Island National Park, 18.VII.1976, Malaise trap, Code 4147-M, W. Reid [3♀, CNC]; Middlesex Co., Coldstream, 22.VI.1922, A. A. Wood [1♂ 5♀, CNC]; Nipissing Co., North Bay, 11.VII.1972, E. J. Kiteley [1♂, CNC]; Peterborough Co., 3.VII.1958, J. F. Brimley [1♀, CNC]; Prescott, Co., Alfred Bog, 7.VI.1982, sweeping vegetation in a bog, L. LeSage [2♂, CNC]; Prince Edward Co., 12.VII.1914, J. F. Brimley [1♀, CNC]; same data, except 29.XI.1914 [1♀, CNC]; same data, except 11.VII.1920 [1♂, CNC]; same data, except 15–30.VII.1922, J. F. Brimley [3♀, CNC]; same data, except 19.VI.1926 [1♀, CNC]; same data, except 6.VII.1935 [1♂, CNC]; same data, except 28.VI.1936 [1♀, CNC]; same data, except 26.VII.1936 [1♀, CNC]; same data, except 17.VIII.1938 [1♀, CNC]; same data, except 9.VII.1941 [1♀, CNC]; same data, except 5.VII.1942 [3♀, CNC]; same data, except 18.VI.1947 [1♀, CNC]; same data, except 29.VIII.1948 [1♀, CNC]; same data, except 29.VI.1949 [1♀, CNC]; same data, except 31.VIII.1949 [1♂, CNC]; same data, except 19.VII.1950 [1♀, CNC]; same data, except VII.1953 [2♂ 4♀, USNM]; same data, except 24.VII.1955 [1♀, CNC]; same data, except 29.VII.1956 [4♂ 10♀, CNC]; same data, except 26.VI.1966 [1♀, CNC]; Renfrew Co., Chalk River, 3.VIII.1937, J. M. Cameron [1♀, LEM]; Russell Co., Mer Bleue, 30.VI.1932, W. J. Brown [1♀, CNC]; same data, except 17.VII.1936 [1♀, CNC]; same data, except 2.VII.1938, G. E. Shewell [1♂, CNC]; Sudbury Co., Sudbury, 4.VIII.1979, R. S. Anderson [1♀, CNC]; Thunder Bay Dist., Black Sturgeon Lake, 1–15.VIII.1956, Lindberg [1♂1♀, CNC]; Jarvis Island, 20.VIII.1952, on white pine, J. F. McAlpine [1♂, CNC]; Manitouwadge, 22.VI.1988, T. Baker [1♂, CNC]; same data, except 30.VII.1992 [1♂, CNC]; Toronto Co., Toronto, 27.V.1896, H. R. [1♀, LEM]; Quetico Provincial Park, 8.VIII.1982, C. B. Barr [2♀, LSAM]; Unknown Co., East Ontario [1♀, CNC].

QUÉBEC: Abitibi Co., Duparquet, 7.VIII.1983, ex. *Pinus banksiana* Lamb., A. Larochelle [1♀, CNC]; Berthier Co., Berthierville, 8.VII.1950, A. Robert [1♀, CEUM]; Lanoraie, VII.1935, G. Chagnon [1♀, H. C. Fall, CEUM]; Charlevoix-Est Co., Clermont, 17.VIII.1982, ex. *Pinus strobus* L., A, Larochelle [2♀, CNC]; Port-au-Saumon, 19.VIII.1982, ex. spruce sp., A. Larochelle [1♂, CNC]; Drummond Co., Saint-Cyrille, 10.VII.1982, ex. *Cassandra calyculata*, L. LeSage [1♂ 1♀, CNC]; Gatineau Co., Wakefield, 5.VIII.1974, ex. *Pinus strobus*, R. Sexton [1♀, CNC]; Île-Jésus Co., Île-Jésus, 5.VII.1935, G. Chagnon [1♀, CEUM]; Montcalm Co., Parc du Mont-Tremblant, 13.VIII.1932, A. Robert [1♂, CEUM]; Montgomery Co., Saint-Jean-d’Orléans, 22.VII.1957, J. L. Laliberté [1♀, IDM]; Montmagny Co. Montmagny, 8.VIII.1981, ex. *Pinus strobus* L., A. Larochelle [1♀, CNC]; Pontiac Co., Beech Grove, 15.VIII.1948, S. D. Hicks [3♀, CNC]; Saguenay Co., Grandes-Bergeronnes, 15.VIII.1982, ex. *Pinus resinosa* Ait., A, Larochelle [1♀, CNC]; Stanstead Co., Barnston, 26.VIII.1984, ex. *Thuya* sp., Larochelle & Larivière [1♀, CNC]; Terrebonne Co., Terrebonne, 1.VII.1933, J. Ouellet [1♀, CEUM]; Vaudreuil Co., Rigaud, 19.VII.1985, ex. *Pinus strobus* L., Larochelle & Larivière [2♀, CNC]; same data except 20.V.1977, sweeping sumac sp., E. J. Kiteley [1♀, CNC]; Saint-Lazare, 6.VIII.1985, ex. *Betula papyrifera* Marsh., A. Larochelle [1♂, CNC].

#### Host plants.

*Pachybrachis obsoletus* may be associated with peat bogs in eastern Canada. Specimens were collected in Alfred Bog (Pope 2011), and Mer Bleue (NCC 2011b; Wikipedia 2011), two well-known bogs of Ontario. The Lanoraie specimens, in Québec, were collected in an ecological preserve, which includes several fens and bogs (MDDEP 2011). Larochelle specimens, also from Québec, had a wide range of potential plant associations. Leather leaf (*Cassandra calyculata* (L.) D. Don.) (Ericaceae) is definitively a host (LeSage, collecting and personal observations), whereas the beetles’ presence on white pine (*Pinus strobus*) (Pinaceae) is very likely incidental. Sweeping from willows (*Salix* spp.) (Salicaceae) was reported by Barney et al. (2011). Additionally, *Pachybrachis obsoletus* was reported as causing light injury to cultivated roses in Saskatoon, Saskatchewan, but the species identification cannot be confirmed (Arnason 1942, 1943; Arnason et al. 1946; King et al. 1944, 1945; Campbell et al. 1989).

#### Comments.

*Pachybrachis obsoletus* has a broad distribution from Manitoba to Oklahoma and eastwards to the Atlantic Ocean, with Alberta and British Columbia disjunct from this main area (Riley et al. 2003). It is reported here for the first time from NB.

### Pachybrachis othonus othonus

(Say, 1825)

http://species-id.net/wiki/Pachybrachis_othonus_othonus

Habitus 11 ; Map 11

Cryptocephalus othonus Say, 1825: pl. 28.
Cryptocephalus marginaticollis Randall, 1838: 46.

#### Recognition.

Body robust. Pronotum black, with all margins and narrow median anterior stripe yellow; each elytron black, with rather narrow sub sutural, discal and marginal vittae yellow; legs yellow. Punctures of pronotum larger and denser than those on elytra; elytral punctures in somewhat regular rows on disc and sides (Habitus 11); male size medium: length 2.63 ± 0.12 mm, width 1.56 ± 0.09 mm.

#### Distribution.

**Map 11.**
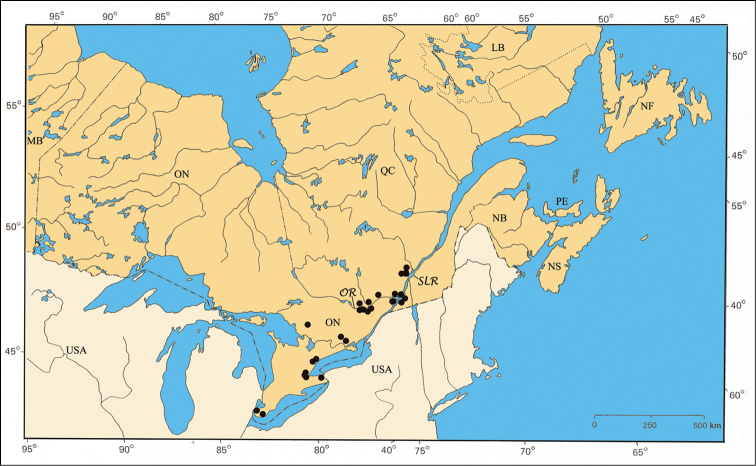
The known distribution of *Pachybrachis othonus othonus* in eastern Canada. OR – Ottawa River; SLR – St. Lawrence River.

A typical eastern species distributed from North Dakota to Texas to the Atlantic Coast in the United States (Riley et al. 2003), and in the south of Ontario and Québec in eastern Canada (Map 11).

#### Material examined.

ONTARIO: Carleton Co., Britannia, 17.VI.1948, S. D. Hicks [1♂, CNC]; same data, except 23.VI.1950, R. de Ruette [1♀, CNC]; Constance Bay, 14.VII.1950, S. D. Hick [1♀, CNC]; Dirleton, 4.VII.1956, S. D. Hicks [1♀, CNC]; Ottawa, 7.VIII.1914, F. G. Ouellet [1♂, IDM]; same data, except 15.VII.1957, J. E. H. Martin [1♂, CNC]; Essex Co., Leamington, 24.VI.1940, W. J. Brown [1♀, CNC]; Ojibway, 27.VI.1943, S. D. Hicks [1♀, CNC]; same data, except 10.VI.1944 [1♂, CNC]; Roseland, 17–24.VI.1944, S. D. Hicks [1♂ 3♀, CNC]; same data, except 17.VI.1946 [2♀, CNC]; same data, except 30.VI.–13.VII.1946 [2♂ 9♀, CNC]; Halton Co., Burlington, 1920, G. M. Stirrett [1♂ 1♀, CNC]; Hastings Co., Chatterton, 16.VII.1950, J. F. Brimley [2♂, CNC]; same data, except 2.VII.1951, J. C. Martin [1♀, CNC]; Marmora, 6.VII.1951, J. F. McAlpine [1♂, CNC]; same data, except 29.VIII.1952, C. Boyle [1♂, CNC]; Lanark Co., Bell’s Corners, 15–26.VI.1950, S. D. Hicks [1♂ 2♀, CNC]; same data, except 4.VII.1950 [1♀, CNC]; Niagara Co., St. Catherines, Decew Falls, 27.VII.1940, S. D. Hick [1♀, CNC]; Toronto Co., Kingsport, 3.VII.1965, D. D. Munroe [1♂ 1♀, CNC]; Toronto, 26.VI.1896, C. T. Hills [1♂ 2♀, LEM]; same data, except 27.V.1896 [1♂ 2♀, LEM]; same data, except 9.VI.1905, E. C. Oakley [3♀, ROM]; same data, except F. Knab [3♂ 1♀, USNM]; Wentworth Co., Ancaster, 10.VII.1965, J. E. Martin [2♂, CNC].

QUÉBEC: Berthier Co., Berthierville, 27.VII.1938, J. Ouellet [1♀, CEUM]; same data, except 8.VII.1950 [1♀, CEUM]; Lanoraie, 1.VII.1932 [1♂, CEUM]; Chambly Co., Boucherville, 1.VII, J. Ouellet [1♀, CEUM]; Deux-Montagnes Co., Saint-Eustache, 12.VIII.1917, J. Ouellet [2♀, CEUM]; Saint-Placide, 13.VII.1931, J. Ouellet [1♀, CEUM]; Gatineau Co., Aylmer, 21.VII.2009, ex. *Hypericum*, *Lythrum*, *Daucus*, graminées, etc, L. LeSage [1♀, CNC]; Île-de- Montréal Co., Montréal, 14.VII.1904, Beaulieu [1♀, USNM]; Joliette Co., Joliette, 12.VII.1909, J. Ouellet [1♀, CEUM]; same data, except 7–13.VII.1922 [29♂ 46♀, CEUM]; Napierville Co., Saint-Rémi, 1.VII.1920, J. Ouellet [1♂ 1♀, CEUM]; Papineau Co., Montebello, 16.VII.1937, J. Ouellet [1♀, CEUM]; Pontiac Co., Beech Grove, 18.VII.1951, J. F. McAlpine [1♀, CNC]; Yarm, 23.VII.1955, C. H. Mann [1♂, CNC]; Vaudreuil Co., Hudson Heights, 24–30.VII.1956, Lindberg [1♀, CNC]; Rigaud, 5.VII.1920, J. Ouellet [2♂ 1♀, CEUM].

#### Host plants.

No specific plant associations were recorded on labels of specimens examined. Chagnon (1937, 1940) and Chagnon and Robert (1962) gave willow (*Salix* sp.) (Salicaceae) as a host in Québec, but *Pachybrachis othonus* was reported on a large number of questionable “hosts” by authors (details in Clark et al. 2004). Barney and Hall (2011) reported feeding, mating and oviposition on *Desmodium marilandicum* (L.) (Fabaceae).

#### Comments.

Balsbaugh (1973) and Riley et al. (2003) recognized *Pachybrachis othonus* as having three subspecies in North America.More information on habitats and hosts are needed on *Pachybrachis othonus othonus*, which is one of the easiest species to recognize.

### Pachybrachis peccans

Suffrian, 1852

http://species-id.net/wiki/Pachybrachis_peccans

**Map 12.**
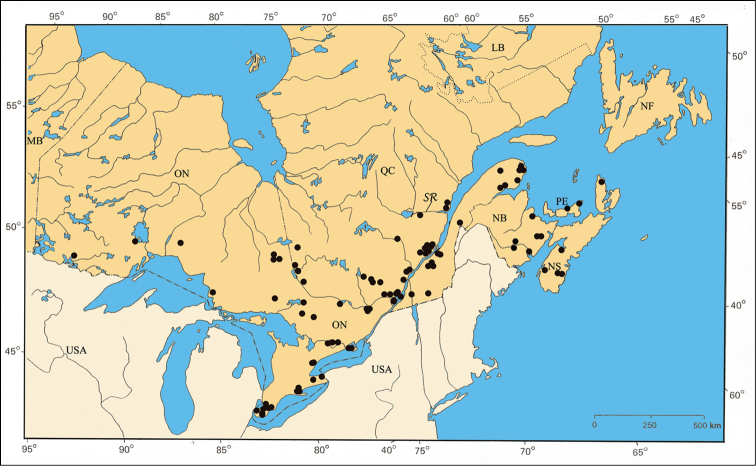
The known distribution of *Pachybrachis peccans* in eastern Canada. SR – Saguenay River.

Habitus 12 ; Map 12 ; Figures 1b , 2a

Pachybrachis peccans Suffrian, 1852: 192.

#### Recognition.

Ocular lines prominent (Figure 2a); males with enlarged foreleg claws (Figures 4a, b); color extremely variable, ranging from yellowish with faint black spots to almost black speckled with small yellow marks (Habitus 12); male size small: length 2.15 ± 0.16 mm, width 1.13 ± 0.09 mm.

#### Distribution.

Transcontinental species, widely distributed from Texas to Yukon to Atlantic Coast (LeSage 1991; Riley et al. 2003). Found in eastern Canada from Ontario to Prince Edward Island (Map 12).

#### Material examined.

NEW BRUNSWICK: Kent Co., Kouchibouguac National Park, 5–6.VII.1977, M. Ivanochko [40♂ 28♀, CNC]; same data, except 5–19.VII.1977, S. J. Miller [2♂ 7♀, CNC]; same data, except 8–13.VIII.1977 [2♂ 1♀, CNC]; same data, except 27.VII.1977, G. A. Calderwood [1♀, CNC]; same data, except 4.VIII.1977 [1♂ 1♀, CNC]; same data, except 16.VI.1978, D. B. Lyons [1♂, CNC]; Kings Co., Mechanic’s Lake, 30.VII.1926 [1♂, CNC]; Penobsquis, 21–31.VII.1926, C. A. Frost [1♂ 2♀, CNC]; Queens Co., Canning Grand Lake near Flowers Cove, 1.VII.2004, D. Sabine and R. Webster [1♂, RWIC]; Saint John Co., St. John, 8.VII.1902, W. McIntosch [1♀, USNM]; same data, except 19.VI.1981, sweeping vegetation, D. R. Ward [1♂ 1♀, CNC]; York Co., New Maryland Charters Settlement, 27.VI.2004, R. P. Webster [1♂, RWIC].

NOVA SCOTIA: Annapolis Co., Annapolis Royal, 21.VII.1928, W. J. Brown [1♂ 1♀, CNC]; Inverness Co., Cape Breton Highlands National Park, Grande Falaise, 30 m, 0.5 km north, 9.VI.1983, forest, flood plain, H. Goulet [1♂, CNC]; Kings Co., Kentville, 20.VI.1981, sweeping, D. Ward [1♀, CNC]; Queens Co., Greenfield Queens, 13.VI.1910, P. G. Bolster [1♀, MCZ]; Port Medway & vic. Queen, 7–20.VII.1910 [2♂ 2♀, MCZ].

ONTARIO: Algoma Dist., Lake Superior Provincial Park, Agawa Bay Campground, 8.VII.1970, ROM Field Party [1♂ 2♀, ROM]; Carleton Co., Britannia, 14.VI.1949, R. de Ruette [1♂, CNC]; Britannia Bay, 3.VI.1959, S. D. Hicks [2♂ 1♀, CNC]; Britannia Heights, 16.VII.1958, S. D. Hicks [1♀, CNC]; same data, except 7.VII.1961, ex. *Populus balsamifera* [17♂ 12♀, CNC]; Constance Bay, 30.VIII.1982, L. J. Milne [1♀, CNC]; Ottawa, 14.VI.1972, F. G. Ouellet [1♀, IDM]; Cochrane Dist., Smoky Falls, Mattagami River, 6.VII.1934, G. S. Walley [1♂, CNC]; Timmins, 16.VI.1982, on plants in gravel, J. Pilny [1♀, CNC]; same data, except Mattagami River, 48°30'N, 81°15'W, 16.VI.1982, Pilny & Motz [2♀, CNC]; Essex Co., Belle River, 26.V.1946, S. D. Hicks [1♀, CNC]; Leamington, 4.VI.1937, G. S. Walley [1♂ 3♀, CNC]; Ojibway, 9.VI.1943, S. D. Hicks [2♂ 2♀, CNC]; same data, except 28.V.1944 [1♂ 1♀, CNC]; Pelee Island, VI.24, R. C. Osburn [1♂, OSU]; same data, except 3.VII.1931, W. J. Brown [2♀, CNC]; same data, except 11.VI.1940, ex. *Salix* [6♂ 4♀, CNC]; Point Pelee, 23–29.VI.1931, W. J. Brown [18♂ 18♀, CNC]; same data, except 3.VII.1931 [1♂ 1♀, CNC]; same data, except 29.V.1940 [3♂ 2♀, CNC]; same data, except 1.VII.1940 [1♂ 2♀, CNC]; same data, except 3.VI.1929, L. J. Milne [5♂ 5♀, CNC]; same data, except 1.VI.1933, ex. willow, G. M. Stirrett [2♂ 4♀, 6, CNC]; same data, except 19–20.V.1955, ex. *Salix interior*, S. D. Hicks [2♂ 8♀, CNC]; same data, except 30.V.1929, G. S. Walley [3♂ 3♀, CNC]; same data, except 25.VI.1920, N. K. Bigelow [1♂ 1♀, ROM]; Point Pelee National Park, 6.VI.1981, ex. *Salix* spp., L. LeSage & D. Ward [1♂, CNC]; same data, except 29.VII.1982, L. LeSage [1♂ 1♀, CNC]; Haldimand Co., Dunville, 30.V.1954, R. Plath [1♂ 1♀, USNM]; Hastings Co., Marmora, 1.VII.1952, sweep from *Rubus* spp., G. P. Holland [1♀, CNC]; Kenora Dist., Malachi, 13–14.VII.1947, W. Y. Watson [1♂ 1♀, ROM]; Willard Lake, 22.VI.1992, lot 1, B.F. & J.L. Carr [1♂, CNC]; Kent Co., Erieau, 26.VI.1932, ex. *Cornus* sp., G. M. Stirrett [5♂ 4♀, CNC]; Rondeau Park, 3.VI.1981, L. LeSage [3♂ 11♀, CNC]; same data, except 4.VI.1981, ex. *Salix* spp. [2♀, CNC]; same data, except 29.V.1985, marsh trail [2♂ 3♀, CNC]; same data, except 28.V.1985, sandy beach [1♀, CNC]; same data, except 7.VI.1981, sifted litter under willows, L. LeSage & D. Ward [4♂ 7♀, CNC]; same data, except 3.VI.1981, sweep willow and grasses, D. Ward [1♂, CNC]; same data, except 5.VI.1985, under willow, J. M. Campbell & A. Davies [1♂, CNC]; Lanark Co., Bell’s Corner, 30.VI.1950, S. D. Hicks [1♀, CNC]; Niagara Co., Ridgeway, A. H. Kilman [1♀, ROM]; Nipissing Dist., Algonquin Park, 18.VI.1922, J. McDunnough [2♀, CNC]; same data, except 4.VII.1965, W. F. O. [1♀, CNC]; North Bay, 11.VII.1972, E. J. Kiteley [1♀, CNC]; North Bay, Trout Creek, 25.VI.1984, willow [1♀, CNC]; Norfolk Co., Hemlock, 9.VIII.1945, ex. *Salix*, G. M. Stirrett [1♂ 1♀, CNC]; Turkey Point, 8.VI.1931, W. J. Brown [2♂ 1♀, CNC]; same data, except 24.VII.1984, sweeping in marshy area, L. LeSage [5♂ 1♀, CNC]; Walsingham Forestry Station, 25.VII.1984, sweeping in ditch, L. LeSage [1♂ 3♀, CNC]; Parry Sound Dist., Burk’s Falls, 14.VII.1926, F. P. Ide [1♀, CNC]; Scotia Junction, 28.VIII.1934, H. W. Wenzel [1♂, OSU]; Peel Co., Port Credit, 4.VII.1908 [1♂, ROM]; Peterborough Co., Hastings, 2.VI.1934, J. F. Brimley [2♀, CNC]; same data, except 31.VI.1934 [1♀, CNC]; same data, except 9.VI.1935 [1♀, CNC]; same data, except 4.VI.1950 [1♀, CNC]; same data, except 5.IX.1956 [2♀, CNC]; Prince Edward Co., same data, except 9–13.VII.1941 [1♀, CNC]; same data, except 24.VII.1945 [1♀, CNC]; Prince Edward Co., Picton, 22.VI.1985, M. Davis [1♀, CNC]; Rainy River Dist., 13–15.VII.1924, J. F. Brimley [2♀, CNC]; same data, except 24.VIII.1924 [1♀, CNC]; Renfrew Co., Petawawa, 17.VI.1980, ex. *Comptonia peregrina*, L. LeSage [1♀, CNC]; Sudbury Dist., Sudbury 1988 [1♂ 1♀, CNC]; same data, except Wickham [1♀, USNM]; Thunder Bay Dist., Black Sturgeon Lake, 1–15.VIII.1956, Lindberg [7♂ 24♀, CNC]; Manitouwadge, 22.VII.1983, T. Baker [2♀, CNC]; same data, except 7.VII.1991, on weeds near woodpile pine logs, [1♂, CNC]; same data, except 21.VI.1992 [1♀, CNC]; 59 km north of junction of highway 516 & 599, 24.VI.1992, lot 2, B.F. & J.L. Carr [1♀, CNC]; Timiskaming Dist., Elk Lake, 30.VI.1958, G. H. Dieke [1♂, USNM]; Toronto Co., Toronto, F. Knab [10♂ 6♀, USNM]; same data, except 2.VII.1894, [1♀, ROM]; same data, except VII.1933 [2♂ 2♀, ROM]; same data, except 30.V.1896, H. R. [1♂ 1♀, LEM]; same data, except 26.VI.1896, C. T. Hills [1♀, LEM]; same data, except 20.VI.1908, R. J. Crew [1♀, ROM]; same data, except 8.VI.1926, E. C. Oakley [1♂ 2♀, LEM]; same data, except VII.1933, L. J. Milne [2♀, UNHC]; Victoria Co., Fenelon Falls, Lindsay, 17.VI.1959, ex. *Salix* sp., F.I.S. [1♂, CNC]; Unknown Co., Sultan road, 68 km west of junction of highway 144, 26.VI.1996, Lot 2, B.F. & J.L. Carr [1♂ 2♀, CNC].

PRINCE EDWARD ISLAND: Kings Co., Souris, 12.VII.1993, ex. lowbush blueberry, M. E. M. Smith [2♂ 1♀, CNC]; Queens Co., PEI National Park Stanhope Campground, 13.VIII.1991, D. S. Chandler [2♂, UNHC].

QUÉBEC: Abitibi Co., Saint-Vital-de-Clermont, 9.VIII.1983, sweeping weeds in *Pinus banksiana* Lamb. forest, A. Larochelle [1♂, CNC]; Argenteuil Co., Saint-Philippe-d’Argenteuil, 21.VI.1983, ex. *Betula populifolia* Marsh., Larochelle [1♂, CNC]; Arthabaska Co., Blandford, 21.VI.1980, ex. *Salix* sp., A. Larochelle [1♀, CNC]; Bellechasse Co., Saint-Étienne, 1.VI.1980, J. C. Aubé [3♂ 2♀, LEM]; Berthier Co., Berthierville, 4.VI.1944, A. Robert [1♀, CEUM]; Bonaventure Co., Carleton, 29.VII.1981, waste land on weeds, A. Larochelle [1♂, CNC]; Cascapédia, 11–14.VI.1933, W. J. Brown [7♂ 12♀, CNC]; Port-Daniel, 30.VII.1981, field on weeds, A. Larochelle [1♂, CNC]; Brome Co., Knowlton, 10–12.VII.1928, G. H. Fish [2♂ 1♀, CNC]; Champlain Co., La Tuque. 8.VIII.1981, ex. *Myrica asplenifolia* L., A. Larochelle [1♀, CNC]; Chicoutimi Co., Jonquière, 27.VI.1970, ex. *Populus tremuloides*, C. Chantz [1♂, AJGC]; Deux-Montagnes Co., La Trappe, 25.V.1929, P. Leopold [1♀, CEUM]; same data, except 27.VI.1933, J. Ouellet [3♂ 1♀, Det. by H. C. Fall, CEUM]; same data, except 2.VII.1933 [2♂ 1♀, CEUM]; same data, except 28.VI.1935 [14♂ 19♀, CEUM]; same data, except 23.VII.1950 [2♂ 2♀, CEUM]; Saint-Placide, 4.VI.1933 [1♀, CEUM]; Gaspé-Est Co., Gaspé, 7.VII.1931 [1♂, Fall-MCZ]; same data, except 25.VII.1954, W. J. Brown [1♂ 2♀, CNC]; Percé, 30.VII.1981, roadside, on weeds, A. Larochelle [1♀, CNC]; Val-d’Espoir, 18.VIII.1939, J. Ouellet [1♀, CEUM]; Gatineau Co., Lytton, 1.VII.1981, ex. *Salix* sp., A. Larochelle [1♀, CNC]; Île-de- Montréal Co., Montréal, 28.VI, J. Ouellet [1♀, CEUM]; same data, except 2.VII.1917 [1♂, CEUM]; same data, except 30.V.1940, A. Robert [2♂ 3♀, CEUM]; same data, except 15.VI.1951, M. Larochelle [1♀, CEUM]; same data, except 17.VI.1980, E. J. Kiteley [1♀, CNC]; same data, except 30.V.1981 [1♂, CNC]; Joliette Co., Joliette, 15.VII.1924 [1♂, Fall- MCZ]; same data, except, 15.VII.1917, J. Ouellet [3♀, CEUM]; same data, except 7–13.VII.1922 [2♂ 2♀, CEUM]; same data, except 7–15.VII.1924 [15♂ 5♀, CEUM]; same data, except 12.VI.1930 [1♂, CEUM]; Kamouraska Co., Sully, 24–26.VI.1936, J. Ouellet [33♂ 45♀, CEUM]; same data, except 1–2.VII.1936 [17♂ 26♀, CEUM]; same data, except 9.VII.1936 [25♂ 24♀, CEUM]; same data, except 13–14.VII.1936 [18♂ 22♀, CEUM]; same data, except 21.VII.1936 [2♂ 4♀, CEUM]; Labelle Co., Lac Saguay, 19.VI.1981, ex. *Salix* sp., A. Larochelle [1♀, CNC]; Nominingue, 25.VIII.1930, J. Ouellet [1♀, CEUM]; same data, except 12–24.VII.1932 [22♂ 37♀, CEUM]; same data, except 24.VII.1933, A. Robert [1♂ 2♀, CEUM]; same data, except 19.VI.1934, L. Daviault [1♂, CEUM]; same data, except 21.VII.1932 [1♀, Fall- MCZ]; Lévis Co., Lauzon, 29.VI.1932, J. Ouellet [1♀, CEUM]; Montcalm Co., Parc du Mont-Tremblant, 15.VIII.1954, A. Robert [1♂ 1♀, CEUM]; same data, except 27.VI.1971, E. J. Kiteley [1♂, CNC]; Nicolet Co., Blandford, 21.VI.1980, A. Larochelle [1♀, CNC]; Papineau Co., Montebello, 3.VII.1937, J. Ouellet [1♀, CEUM]; Portneuf Co., Lac Sergent, 2.VII.1961, J. L. Laliberté [1♀, IDM]; Saint-Augustin, 17.VI.1967, J. L. Laliberté [1♂, IDM]; same data, except 22.VI.1977 [1♂, IDM]; Sainte-Catherine, 8–14.VII.1956, J. C. Aubé [7♂ 4♀, LEM; 2♂ 2♀, AMNH, 1♂, USNM]; same data, except 19.VI.1957 [1♂, LEM]; same data, except 11.VI.1960 [1♀, LEM]; same data, except 9–15.VII.1960 [1♂ 1♀, LEM; 1♀, USNM]; same data, except 26.VII.1961 [2♀, LEM]; same data, except 5.VIII.1961 [1♀, LEM]; same data, except 15.VII.1956, J. L. Laliberté [1♂ 2♀, IDM]; Québec Co., Cap-Rouge, 27.VI.1959, J. C. Aubé [1♂, LEM]; same data, except 24.VI.1981, D. R. Ward [1♀, CNC]; Lac Beauport, 11–23.VII.1956, J. L. Laliberté [1♂ 2♀, IDM]; Québec, 15.VI.1957, J. C. Aubé [1♂, LEM; 1♂, AMNH]; Saguenay Co., Grandes- Bergeronnes, 20.VII.1981, field on weeds, A. Larochelle [1♀, CNC]; Rivière Barthélemy, 22.VII.1981, field on weeds, A. Larochelle [1♂, CNC]; Tadoussac, 23.VII.1932, A. F. Winn [3♀, CEUM]; Saint-Jean Co., Saint-Jean-sur-Richelieu (as “St. Johns”), F. Knab [1♂, USNM]; Saint-Maurice Co., Pointe-du-Lac, 11.VII.1926, J. L. Laliberté [1♀, IDM]; same data, except 5.VIII.1927 [1♂ 1♀, IDM]; same data, except 26.VII.1928 J. L. Laliberté [1♀, IDM]; same data, except 20.VII.1936 [2♀, IDM]; Soulanges Co., Rivière-Beaudette, 10.VIII.1985, Larochelle & Larivière [1♀, CNC]; Témiscamingue Co. Laniel, 5–6.VI.1963, W. Gagné [1♂, CNC]; Notre- Dame-du-Nord, 36.VI [2♂, CEUM]; Vaudreuil Co., Rigaud, 13.VII.1973, sweeping *Salix* sp., E. J. Kiteley [3♂ 3♀, CNC]; same data, except 7.VI.1985, willow in flower (*Salix* sp.), Larochelle & Larivière [1♂, CNC]; same data, except 7.VI.1985, old field birch (*Betula populifolia* Marsh.), Larochelle & Larivière [1 ♀, CNC]; Saint-Lazare, 20.VI.1983, ex. *Salix fragilis* L., A. Larochelle [1♀, CNC]; Mont Lyall 1500 ft, 31.VII.1933, W. J. Brown [2♂ 4♀, CNC].

#### Host plants.

Large series of *Pachybrachis peccans* were reportedly taken on *Populus balsami-fera* L. and *Salix* spp. (Salicaceae). Other plant associations recorded on labels were *Populus tremuloides* Michx.; lowbush blueberry (*Vaccinium angustifolium* Ait.) (Ericaceae); *Rubus* spp. (Rosaceae); and *Comptonia peregrina* (L.) (Myricaceae). Larvae were reared on dead leaves of sand-bar willow (*Salix interior* Rowlee) by LeSage (1985). Larochelle specimens from Québec had a wide range of potential plant associations. Several additional potential hosts are listed by Clark et al. (2004) from their literature review.

#### Comments.

*Pachybrachis peccans* was the most commonly examined species (over 900 specimens; Table 1). It has also the largest distribution, being found from Nova Scotia to Yukon (Map 12). *Pachybrachis peccans* is reported here for the first time from PE.

**Table 1.** List of *Pachybrachis* species recorded from eastern Canada, with number of specimens examined per province. No specimens were found bearing a label from Newfoundland or Labrador.<br/>

	**ON**	**QC**	**NB**	**NS**	**PE**	**Total**
*Pachybrachis atomarius* (F. E. Melsheimer)	32	1	0	0	0	33
*Pachybrachis bivittatus* (Say)	59	43	0	0	0	102
*Pachybrachis calcaratus* Fall	2	0	0	0	0	2
*Pachybrachis cephalicus* Fall*	25**	0	0	0	0	25
*Pachybrachis hepaticus hepaticus* (F. E. Melsheimer)	19	91	0	0	0	110
*Pachybrachis luctuosus* Suffrian*	17**	3**	0	0	0	20
*Pachybrachis luridus* (Fabricius)	66	0	0	0	0	66
*Pachybrachis m-nigrum* (F. E. Melsheimer)	0	4	1	0	0	5
*Pachybrachis nigricornis* (Say)	143	82	11	0	0	236
*Pachybrachis obsoletus* Suffrian	87	22	14**	0	0	123
*Pachybrachis othonus othonus* (Say)	59	94	0	0	0	153
*Pachybrachis peccans* Suffrian	337	483	93	9	5**	927
*Pachybrachis pectoralis* (F. E. Melsheimer)	15	36	0	0	0	51
*Pachybrachis spumarius* Suffrian	179	142	0	0	0	321
*Pachybrachis subfasciatus* (J. E. LeConte)	18	0	0	0	0	18
*Pachybrachis tridens* (F. E. Melsheimer)	8	0	0	0	0	8
*Pachybrachis trinotatus* (F. E. Melsheimer)	53	11	0	0	0	64
Total per province	1119	1012	119	9	5	2264

*New national record for Canada,

**New provincial record

As one of several species in North America having distinct ocular lines, enlarged claws, and varying degrees of maculation, there is much potential confusion with other species. Fall (1915) stated, “While *Pachybrachis peccans* varies toward *Pachybrachis melanostictus* in its darker individuals, it approaches so closely to *Pachybrachis diversus* and *Pachybrachis abdominalis* in some of its paler forms as to make distinction purely discretional.” At this time, we believe *Pachybrachis diversus* is a more southern species not found in Canada, and *Pachybrachis abdominalis* and *Pachybrachis melanostictus* are not found in eastern Canada.

### Pachybrachis pectoralis

(F. E. Melsheimer, 1847)

http://species-id.net/wiki/Pachybrachis_pectoralis

**Map 13.**
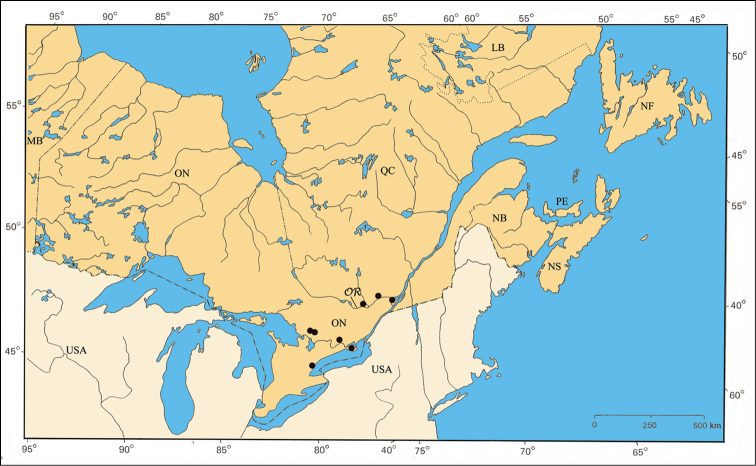
The known distribution of *Pachybrachis pectoralis* in eastern Canada. OR – Ottawa River.

Habitus 13 ; Map 13 ; Figure 1a

Cryptocephalus pectoralis F. E. Melsheimer, 1847: 171.
Pachybrachis sobrinus Haldeman, 1849: 262.
Pachybrachis oculatus Suffrian, 1852: 178.
Pachybrachis sticticus Blatchley, 1910: 1130.

#### Recognition.

Form, especially of the male, narrower than usual; color dull yellow, maculate with brown or black (Habitus 13); surface not or scarcely shining; eyes narrowly separated; ocular lines present (Figure 1a); front tibiae sinuate on inner margin beyond middle; front claws of male obviously, though not greatly, enlarged; male size small: length 1.92 ± 0.07 mm, width 0.96 ± 0.04 mm.

#### Distribution.

Eastern species distributed from Nebraska to Texas to Atlantic Coast in the United States (Riley et al. 2003). In eastern Canada, *Pachybrachis pectoralis* has been found in southern Ontario and the Ottawa Valley in Québec (Map 13).

#### Material examined.

ONTARIO: Hastings Co., 10.vii.1938, J. F. Brimley [2♀, CNC]; same data, except 5.VIII.1957 [1♀, CNC]; Muskoka Dist., Norway Point Lake of Bays, 30.XI.1922, J. McDunnough [1♀, CNC]; Prince Edward Co., 3.VIII.1938, J. F. Brimley [3♂ 3♀, CNC]; Kawartha Gull Lake, 2.VIII.1943, J. F. Brimley [2♂, CNC]; same data, except 19.VII.1950 [1♀, CNC]; Toronto Co., Toronto, 15–30.VI.1927, L. J. Milne [1♀, CNC]; Unknown Co., East Ontario [1♂, CNC].

QUÉBEC: Gatineau Co., Mont King, Parc de la Gatineau, 19.VIII.1996, L. LeSage [8♂ 13♀, CNC]; same data, except 6.IX.1996 [1♀, CNC]; same data, except 28.V.1997 [1♀, CNC]; same data, except 21.VII.1997 [1♂ 3♀, CNC]; Pontiac Co., Luskville, 4.VII.1985, ex. *Quercus rubra* L. Larochelle & Larivière [1♀, CNC]; same data except 30.VII.1985, ex. *Quercus alba* L. [1♂, CNC]; Vaudreuil Co., Rigaud, A. Robert [2♂ 2♀, CEUM]; same data, except 20.VIII.1984, ex. *Quercus rubra* L., Larochelle & Larivière [2♂, CNC]; same data except 19.VIII.1985, ex. *Quercus rubra* L., Larochelle & Larivière [1♀, CNC].

#### Host plants.

No information is available from the specimens examined. According to Blatchley (1924a, b), Fall (1915), Barney (1984) and Clark (2000), *Pachybrachis pectoralis* is associated with common locust (*Robinia pseudoacacia* L.) (Fabaceae). Larochelle’s specimens were found on *Quercus alba* L. and *Quercus rubra* L. Additional potential hosts are given in Clark et al. (2004).

#### Comments.

No specimens from New Brunswick and Nova Scotia, from where this species was reported by LeSage (1991) and Riley et al. (2003), were located, although the material of all the important collections of eastern Canada was examined. The Ottawa River Valley is the northernmost limit of *Pachybrachis pectoralis*, in Québec. This species may extend further north to the Maritime Provinces along the Atlantic Coast, but it has not yet been recorded from Maine (Riley et al. 2003; Majka et al. 2011).

### Pachybrachis spumarius

Suffrian, 1852

http://species-id.net/wiki/Pachybrachis_spumarius

**Map 14.**
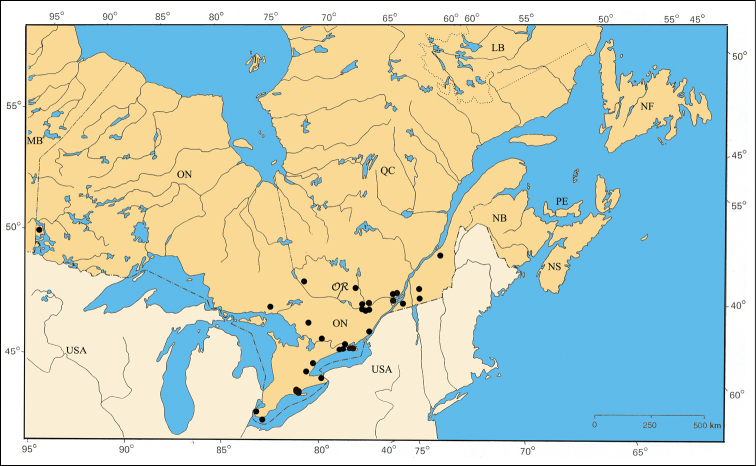
The known distribution of *Pachybrachis spumarius* in eastern Canada. OR – Ottawa River.

Habitus 14 ; Map 14 ; Figures 2b , 5b , 6b , 7d , 8c , 10b, 10c

Pachybrachys spumarius Suffrian, 1852: 179.
Pachybrachis roboris Fall, 1915: 420.

#### Recognition.

Elytra and pronotum with small yellow spots and diffuse rufous mottled marks; prothoracic puncturation dense, extending to side margins (Habitus 14); ocular lines absent; aedeagus tubular with terminal nodule (Figure 10b) or nipple-shaped apex when seen from above (Figure 10c); male size small: length 1.91 ± 0.13 mm, width 1.06 ± 0.05 mm.

#### Distribution.

Eastern species distributed from southern Saskatchewan to Texas to Atlantic Coast (Riley et al. 2003), and present in southern Ontario and Québec in eastern Canada. The Ottawa Valley and the south of eastern Townships, in Québec, are probably the northernmost distribution limit of *Pachybrachis spumarius* (Map 14).

#### Material examined.

ONTARIO: Carleton Co., Britannia, 28.VI.1931, L. J. Milne [1♂ 1♀, UNHC]; same data, except 19.VII.1949, R. de Ruette [1♀, CNC]; Britannia Heights, 16.VII.1958, S. D. Hicks [1♂, CNC]; Constance Bay, 10.VII.1941, W. J. Brown [1♀, CNC]; Carp, 5.VII.1932, W. J. Brown [1♂, CNC]; Essex Co., Ojibway, 24.VI.1945 [1♀, CNC]; Pelee Island, 3.VII.1931, W. J. Brown [1♂ 1♀, CNC]; Roseland, 26.VI.1944, S. D. Hicks [1♀, CNC]; Hasting Co., 2.IX.1934, J. F. Brimley [1♂ 1♀, CNC]; same data, except 25.VII.1954 [1♂, CNC]; Leeds Co., Lindsay Island, Saint Lawrence Island National Park, 15.VII.1976, ex. *Betula papyrifera*, W. Reid [2♂, CNC]; Mermaid Island, Saint Lawrence Island National Park, 23.VII.1976, W. Reid [1♀, CNC]; Lennox & Addington Co., 16.VII.1939, J. F. Brimley [1♀, CNC]; same data, except 6.IX.1948 [1♀, CNC]; same data, except 10.VII.1949 [3♀, CNC]; Lincoln Co., DeCew Falls, 27.VII.1940, S. D. Hicks [1♀, CNC]; Norfolk Co., Turkey Point Provincial Park, 24.VII.1984, sweeping in mixed forest, L. LeSage [3♂, CNC]; Walsingham, 11.VII.1956, W. J. Brown [6♂ 7♀, CNC]; Walsingham Forest Station, 25.VII.1984, ex. *Rhus typhina*, L. LeSage [45♂ 47♀, CNC]; Northumberland Co., 2.IX.1950, J.F. Brimley [1♀, CNC]; Prince Edward Co., 10.VII.1935, J. F. Brimley [3♂ 3♀, CNC]; same data, except 21–25.VII.1937 [5♂ 9♀, CNC]; same data, except 3.VII.1938 [1♀, CNC]; same data, except 13.VII.1941 [1♂, CNC]; same data, except 16.VII.1947 [6♂ 2♀, CNC]; same data, except 5–19.VII.1950 [3♂ 3♀, CNC]; same data, except 5.VII.1953 [1♀, CNC]; same data, except 7.VII.1954 [1♂ 1♀, CNC]; Clearwater Bay, 30.VII.1996, sweeping miscellaneous vegetation, B. F & J. L. Carr [2♀, CNC]; Toronto Co., Toronto, 15.VIII.1908, R. J. Crew [1♀, ROM]; Kelly Lake, 26.VII.1933, L. J. Milne [1♂, UNHC]; Victoria Co., Coboconk, 2.VIII.1948, J. F. B. [2♂ 1♀, CNC]; Unknown Co., East Ontario [1♂ 1♀, CNC].

QUÉBEC: Bagot Co., Saint-Pie, 1.VII.1985, ex. *Acer rubrum* L., Larochelle & Larivière [1♂, CNC]; Châteauguay Co., Ormstown, 12.VII.1977, sweeping sumac, E. J. Kiteley [3♂ 2♀, CNC]; Deux-Montagnes Co., La Trappe, 19–22.VII.1945, J. Ouellet [1♂ 1♀, CEUM]; same data, except 7.VII.1946, [1♀, CEUM]; same data, except 13–15.VII.1949 [2♂ 4♀, CEUM]; same data, except 8–23.VII.1950, ex. Virginia sumac [27♂ 35♀, CEUM]; same data, except 1–8.VIII.1950, ex. Virginia sumac [9♂ 5♀, CEUM]; same data, except 27.VII.1951 [6♂ 7♀, CEUM]; Gatineau Co., Mont-King, Parc de la Gatineau, 19.VII.1981, P. Bélanger [1♂ 2♀, LFC]; Kazabazua, 3.IX.1967, H. J. Teskey [1♀, CNC]; Missisquoi Co., Phillipsburg, 15–19.VII.1969, J. L. Laliberté [1♂ 1♀, IDM]; same data, except 29.VII.1972 [3♂ 4♀, IDM]; same data, except 22.VI.1975 [2♂, IDM]; Pontiac Co., Luskville, 30.VII.1985, ex. *Quercus alba* L., Larochelle & Larivière [1♀, CNC]; Témiscamingue Co., Laniel, 14.VIII.1932, W.J. Brown [1♀, CNC]; Vaudreuil Co., Hudson Heights, 24–30.VII.1956, Lindberg [1♂ 1♀, CNC]; Rigaud, 26.VII.1902, F. Knab [1♂ 1♀, USNM]; same data, except 25.VII.1939, A. Robert [1♂, CEUM]; same data, except 15.VIII.1972, on sumac, E. J. Kiteley [3♂, CNC]; same data, except 13.VII.1973, sweeping sumac [1♂ 3♀, CNC]; same data, except 22.VII.1974, on sumac [3♂ 2♀, CNC]; same data, except 27.VIII.1977, on sumac [1♂ 1♀, CNC]; same data, except 16.VIII.1984, ex. *Rhus typhina* L., Larochelle & Larivière [1♀, CNC]; Saint-Lazare, 9.VIII.1982, UV light in a pine plantation, A. Larochelle [1♀, CNC].

#### Host plants.

Several specimens were recorded as being collected from *Rhus typhina* L., Virginia sumac, or just sumac (Anachardiaceae), which are various names for the same plant. Barney and Hall (2011) reported collecting specimens in abundance on *Rhus copallina* L. and *Rhus glabra* L., and observed feeding, mating and oviposition on these species in the laboratory. Larochelle cited *Acer rubrum* L. (Aceraceae) and *Quercus alba* L. (Salicaceae) as potential hosts on his labels. *Betula papyrifera* Marsh. (Betulaceae) was also reported here but cannot be confirmed as a plant association.

#### Comments.

*Pachybrachis spumarius* is the second most commonly collected species in eastern Canada and can often be found in large series on *Rhus* spp. (Table 1).

### Pachybrachis subfasciatus

(J. E. LeConte, 1824)

http://species-id.net/wiki/Pachybrachis_subfasciatus

Habitus 15 ; Map 15

Cryptocephalus subfasciatus J. L. LeConte, 1824: 173.
Pachybrachys biguttatus Suffrian, 1852: 167.
Pachybrachys impurus Suffrian, 1852: 186.
Pachybrachys xanthias Suffrian, 1852: 199.
Pachybrachys impurus var. *Pachybrachis umbrosus* Fall, 1915: 379.

#### Recognition.

Color dark, pronotum with sides narrowly yellow; elytra with red or yellow, more or less broad, irregular or indented transverse median fascia often interrupted at suture, and with red or yellow apical spot (Habitus 15); Disc of pronotum often, and head more rarely, variegated with reddish yellow; male size small: length 2.21 ± 0.09 mm, width 1.18 ± 0.06 mm.

#### Distribution.

**Map 15.**
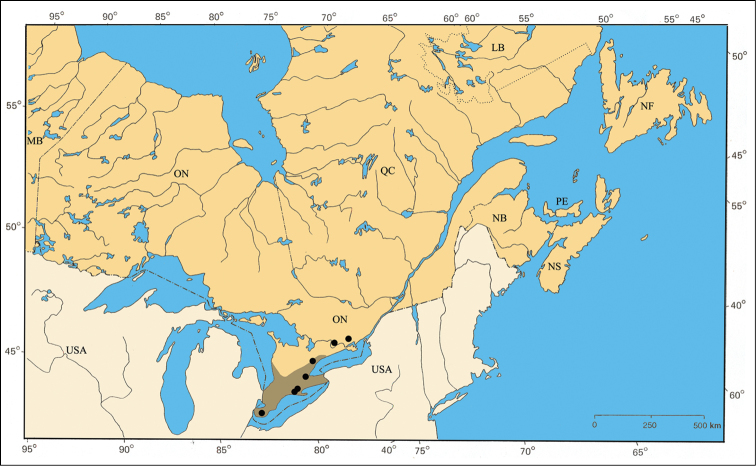
The known distribution of *Pachybrachis subfasciatus* in eastern Canada. Carolinian Zone in dark beige

Eastern species distributed from Kansas to Louisiana to Atlantic Coast in the United States (Riley et al. 2003), restricted to the Carolinian Zone in southern Ontario in eastern Canada (Map 15).

#### Material examined.

ONTARIO: Essex Co., Leamington, 6.VII.1931, G. S. Walley [1♀, CNC]; same data, except 9.VI.1937 [1♀, CNC]; Hastings Co., 19.VII.1938, J. F. Brimley [2♀, CNC]; Lennox & Addington Co., 16.VII.1938, J. F. Brimley [1♂, CNC]; Norfolk Co., Normandale, 5.VI.1931, W. J. Brown [1♀, CNC]; Walsingham, 3.VI.1944, W. J. Brown [3♀, CNC]; same data, except 11.VII.1956 [2♀, CNC]; Northumberland Co., Hamilton, 15.VII.1981, M. Sanborne [1♂, CNC]; Toronto Co., Toronto, 15.VI.1896 [3♀, LEM]; same data, except 30.V.1897, C. T. Hills [1♂ 1♀, LEM].

#### Host plants.

No records are available from the specimens examined. Downie and Arnett (1996) reported *Pachybrachis subfasciatus* from black walnut (*Juglans nigra* L.) (Juglandaceae).

#### Comments.

*Pachybrachis subfasciatus* is another possible example of a species once living in the Carolinian Zone which may have been extirpated from the Canadian fauna. No specimens were collected in the last 55 years. If its association with black walnut is correct, this may explain its rarity, or even extinction, since Fox and Soper (1953) reported this tree occurring naturally only in rich woods in southernmost Ontario and considered recent trees above these limits to have been planted.

### Pachybrachis tridens

(F. E. Melsheimer, 1847)

http://species-id.net/wiki/Pachybrachis_tridens

Habitus 16 ; Map 16

Cryptocephalus tridens F. E. Melsheimer, 1847: 172.
Cryptocephalus flavicornis F. E. Melsheimer, 1847: 172.
Pachybrachys mollis Haldeman, 1849: 263.

#### Recognition.

Color pale yellow with broad, sharply limited, black markings; antennae (usually) and legs entirely yellow (Habitus 16). Lustre dull. Eyes separated by about twice length of basal antennomere in male, and by two and one-half to three times length of this antennomere in female. Ocular lines fine. Front claws of male not enlarged (as in Figure 4c); male size small: length 1.93 ± 0.10 mm, width 1.01 ± 0.04 mm.

#### Distribution.

**Map 16.**
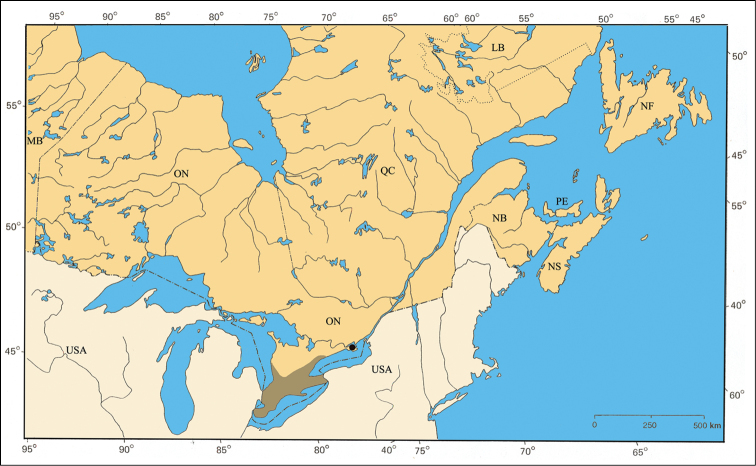
The known distribution of *Pachybrachis tridens* in eastern Canada. Carolinian Zone in dark beige.

*Pachybrachis tridens* is an eastern species distributed from Manitoba to Texas to the Atlantic Coast in the United States (Riley et al. 2003), and restricted to the Carolinian Zone of southern Ontario in eastern Canada (Map 16).

#### Material examined.

ONTARIO: Prince Edward Co., 21–28.VI.1950, J. F. Brimley [3♂ 5♀, CNC].

#### Host plants.

No records are available from the specimens examined. Poison ivy (*Toxicodendron radicans* (L.) Kuntze) (Anachardiaceae) was given as the preferred host by Fall (1915), Blatchley (1924a), Wilcox (1954, 1979), Furth (1985), and others. A complete list of citations is found in Clark et al. (2004).

#### Comments.

Since *Pachybrachis tridens* has not been collected in the last 55 years, it can be considered as extirpated from the Canadian fauna. Formerly, it was probably restricted to the Carolinian Life Zone, which is now reduced to minute remnants. For this reason, the Manitoba record reported by LeSage (1991) and Riley et al. (2003), and the Québec record cited by Riley et al. (2003) are questionable. No specimens were available to confirm them.

### Pachybrachis trinotatus

(F. E. Melsheimer, 1847)

http://species-id.net/wiki/Pachybrachis_trinotatus

Habitus 17 ; Map 17

Cryptocephalus trinotatus F. E. Melsheimer, 1847: 170.

#### Recognition.

Pronotum red with heavy, sharply defined, black M-mark, and with pale anterior and lateral margins; elytra entirely black (Habitus 17); male size large: length 3.09 ± 0.13 mm, width 1.75 ± 0.09 mm.

#### Distribution.

**Map 17.**
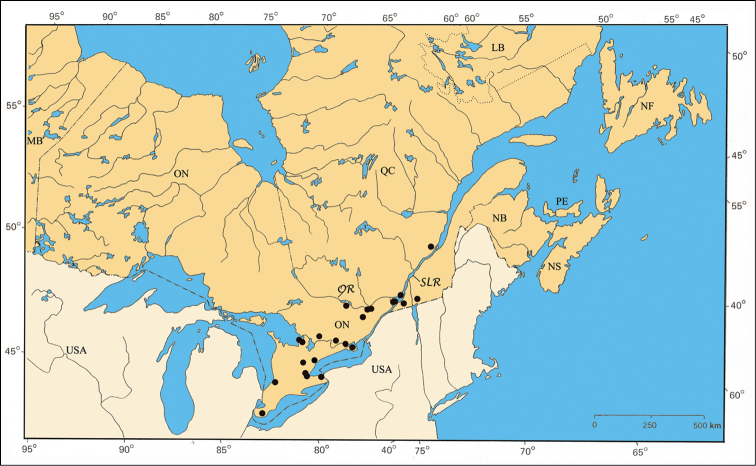
The known distribution of *Pachybrachis trinotatus* in eastern Canada.

*Pachybrachis trinotatus* is an eastern species distributed from Kansas to the Atlantic Coast in the United States (Riley et al. 2003), and in Ontario and Québec in eastern Canada (Map 17).

#### Material examined.

ONTARIO: Carleton Co., Stittsville, 26.VII.1961, G. Brumpton [1♂, CNC]; Essex Co., Leamington, 27.VI-3.VII.1931, W. J. Brown [1♂ 1♀, CNC]; Hamilton Co., Ancaster, 2.VII.1958, J. E. H. Martin [1♂, CNC]; Hastings Co., 10.VII.1938, J. F. Brimley [1♀, CNC]; same data, except 24.VII.1960, J. F. Brimley [1♂ 1♀, CNC]; Marmora, 2.VII.1952, C. Boyle [1♀, CNC]; Lambton Co., Grand Bend, 10.VII.1939, G. E. Shewell [1♂ 1♀, CNC]; Lanark Co., Bell’s Corners, 15.VII.1954, S. D. Hicks [3♂ 2♀, CNC]; same data, except Lanark, Kerr Lake 13.VII.1975 [1♂, CNC]; Lincoln Co., DeCew Falls, 29.VI.1940, S. D. Hicks [1♂, CNC]; same data, except 27.VII.1940 [1♂ 1♀, CNC]; same data, except VIII.1941 [1♂, CNC]; Northumberland Co., Hamilton, 14–19.VII.1984, M. Sanborne [1♀, CNC]; Prince Edward Co., 21.VII.1937, beaten from oak, J. F. Brimley [1♀, CNC]; same data, except 4.VII.1946 [1♀, CNC]; same data, except 11.VIII.1947 [1♀, CNC]; same data, except 14.VII.1948 [1♀, CNC]; same data, except 10.VIII.1948 [1♀, CNC]; same data, except 20.VI.1949 [1♂, CNC]; same data, except 6.VII.1949 [1♀, CNC]; same data, except 17.VII.1950 [5♂ 3♀, CNC; 1♂, AMNH; 2♂ 1♀, St. John’s wort blossom, FSCA]; same data, except 7.VII.1955 [1♂, CNC]; same data, except 29.VII.1956 [1♀, CNC]; same data, except 27.VII.1962 [1♂ 1♀, CNC]; Renfrew Co., Hwy 512 15 km W Eganville, 5.VII.1996, B. F. & J. L. Carr [1♂, CNC]; Simcoe Co., Craighurst, 30.VIII.1963, G. G. E. Scudder [1♀, CNC]; Tiny Township, Cawaja Beach, 17.VII.1968, J. C. E. Riotte [1♂ 1♀, ROM]; Toronto Co., Toronto, Kelly Lake, 13–26.VII.1933, L. J. Milne [2♂ 1♀, UNHC]; Victoria Co., Coboconk, 7.VIII.1940, S.D. Hicks [1♀, CNC]; Wellington Co., Guelph, VII.1924, D. C. B. Duff [1♂, ROM].

QUÉBEC: Huntingdon Co., Covey Hill, 1.VII.1927, W. J. Brown [1♂, CNC]; Île-de-Montréal, Montréal, 10.VII.1977, sweeping field, E. J. Kiteley [1♂, CNC]; Missisquoi Co., Phillipsburg, 31.VII.1972, J. L. Laliberté [1♂, IDM]; Québec Co., Québec, 26.VII.1902, F. Knab [1♂ 1♀, USNM]; Vaudreuil Co., Hudson Heights, 24–30.VII.1956, Lindberg [1♀, CNC]; Rigaud 29.VI.1907, J. Ouellet [1♂, CEUM]; same data, except 18.VIII.1921 [2♂, CEUM]; same data, except 23.VII.1974, E. J. Kiteley [2♀, CNC].

#### Host plants.

No information was available from specimens examined. Barney and Hall (2011) reported handpicking specimens from St. John’s wort, *Hypericum punctatum* L. (Clusiaceae), and observed feeding, mating and oviposition on *Hypericum punctatum*, *Hypericum perforatum* L. and *Hypericum dolabriforme* Vent. in the lab. Following Banks (1912), New Jersey tea, *Ceanothus americanus* L. (Rhamnaceae) was often given by authors as a host for *Pachybrachis trinotatus* (complete citation in Clark et al. 2004).

#### Comments.

With its black elytra and reddish pronotum ornamented with a large, black, M-shaped marking, *Pachybrachis trinotatus* is very easily distinguished from all other Canadian species of the genus (Habitus 17). It is widely distributed in southern Ontario but is found only in the Ottawa River Valley and south of the eastern Townships in Québec (Map 17). Both areas very likely represent its northernmost distribution limit in this province.

## Conclusion

According to the *Catalog of Leaf Beetles of America North of Mexico* (Riley et al. 2003), there are 17 species of *Pachybrachis* in the eastern provinces. This study verified 15 of those species (all except *Pachybrachis praeclarus* and *Pachybrachis relictus*), and discovered two new national and provincial records, both from southernmost Ontario: *Pachybrachis cephalicus* and *Pachybrachis luctuosus*. *Pachybrachis obsoletus* is new to NB, and *Pachybrachis peccans* is new to PE.

The *Pachybrachis relictus* records cited by Fall (1915) (ON: Toronto, Blaisdell Coll.; Scotia Junction, July 27, Wenzel) cannot be confirmed, and there is no evidence *Pachybrachis praeclarus* ever existed in eastern Canada.

A review of the distribution and abundance of the seventeen *Pachybrachis* species reveals four general groups: (1) species distributed from Ontario into at least one province in the Maritimes (*Pachybrachis nigricornis*, *Pachybrachis obsoletus* and *Pachybrachis peccans*); (2) species distributed along the shores of the Great Lakes (Erie, Michigan and Ontario) and rivers (Ottawa, Saguenay and St. Lawrence), but unknown from central and northern ON and QC (*Pachybrachis bivittatus*, *Pachybrachis hepaticus hepaticus*, *Pachybrachis othonus othonus*, *Pachybrachis pectoralis*, *Pachybrachis spumarius* and *Pachybrachis trinotatus*); (3) rare species exclusively from southern ON (*Pachybrachis calcaratus*, *Pachybrachis cephalicus*, *Pachybrachis luridus*, *Pachybrachis subfasciatus* and *Pachybrachis tridens*) and/or with an additional disjunct population in QC (*Pachybrachis atomarius* and *Pachybrachis luctuosus*); and (4) species having the northernmost extension of an eastern US distribution into the southeastern Townships of QC (*Pachybrachis m-nigrum*). There are no *Pachybrachis* that could be considered arctic, subarctic, or boreal species; no specimens were found from Labrador or Newfoundland; and all species had southern affinities.

*Pachybrachis bivittatus*, *Pachybrachis hepaticus* and *Pachybrachis peccans* are transcontinental species extending from the Atlantic to the Pacific Oceans. They are common across eastern Canada, and have been collected rather recently (1990s – present).

A large group of species found in this study share a similar eastern United States *Pachybrachis* distribution, occurring from the Atlantic coastal states into the Great Plains: *Pachybrachis atomarius*, *Pachybrachis luridus*, *Pachybrachis m-nigrum*, *Pachybrachis nigricornis*, *Pachybrachis obsoletus*, *Pachybrachis othonus othonus*, *Pachybrachis pectoralis*, *Pachybrachis spumarius*, *Pachybrachis subfasciatus*, *Pachybrachis tridens*, and *Pachybrachis trinotatus* (Riley et al. 2003; Barney, unpublished data). *Pachybrachis nigricornis*, *Pachybrachis obsoletus*, *Pachybrachis othonus othonus*, and *Pachybrachis pectoralis* have all been collected within the last 20 years and probably have viable populations, but *Pachybrachis atomarius*, *Pachybrachis calcaratus*, *Pachybrachis luridus*, *Pachybrachis subfasciatus*, *Pachybrachis tridens*, and *Pachybrachis trinotatus* have not been collected in over 30 years (*Pachybrachis luridus* in over 70 years) and may be considered extirpated from eastern Canada.

The remaining species, *Pachybrachis calcaratus*, *Pachybrachis cephalicus*, and *Pachybrachis luctuosus*, were from the relatively small, southern Carolinian Ecozone, but their North American distribution is not as well defined or widespread as the others (Riley et al. 2003; Barney, unpublished data). *Pachybrachis calcaratus* has not been collected in Canada since 1944 and is very likely extirpated from Canadian fauna. *Pachybrachis cephalicus* may survive in its refugium in the Walsingham Forest (ON). *Pachybrachis luctuosus* was collected recently in southern Ontario and is probably still surviving there, but we have no recent information on the disjunct population in the Ottawa Valley.

Of course, any faunal survey such as this is only as good as the naturalists and collectors out in the field. There have been five major collectors of eastern Canadian *Pachybrachis*: J. Ouellet, 680 specimens during 1900s to 1940s; J. F. Brimley, 257 specimens during 1910s to 1950s; W. J. Brown, 234 specimens during 1920s to 1950s; S. D. Hicks, 120 specimens during 1940s and 1950; and L. LeSage, 224 specimens during 1980s to 2000s. This demonstrates that 57% of all eastern Canada *Pachybrachis* ever collected were found by four collectors between 1900 and 1959. The loss of habitat appears to be accompanied by a loss of people monitoring the habitats. Hopefully, the species cited above as possibly being extirpated from eastern Canada are still out there waiting to be rediscovered.

One of the consequences of global warming of the climate is that many plant and animal species will move northward. Woodall et al. (2009) stated that the process of northward migration of trees in the eastern United Stated is currently underway. According to Diffenbaugh et al. (2008) and Woodall et al. (2009), the relaxed cold limitations and a greater accumulation of degree-days should favor several herbivores, but native *Pachybrachis* species and their host plants could benefit as well of expected warmer conditions.

## Legends for habitus

**Habitus 1.**
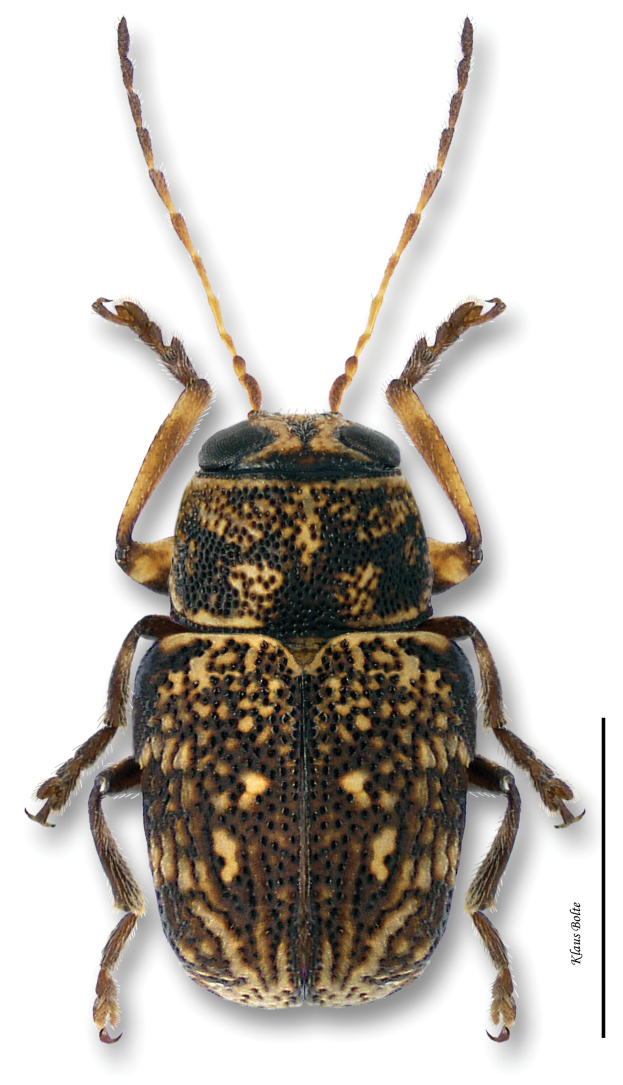
Dorsal habitus of *Pachybrachis atomarius*. Scale bar, 1mm.

**Habitus 2.**
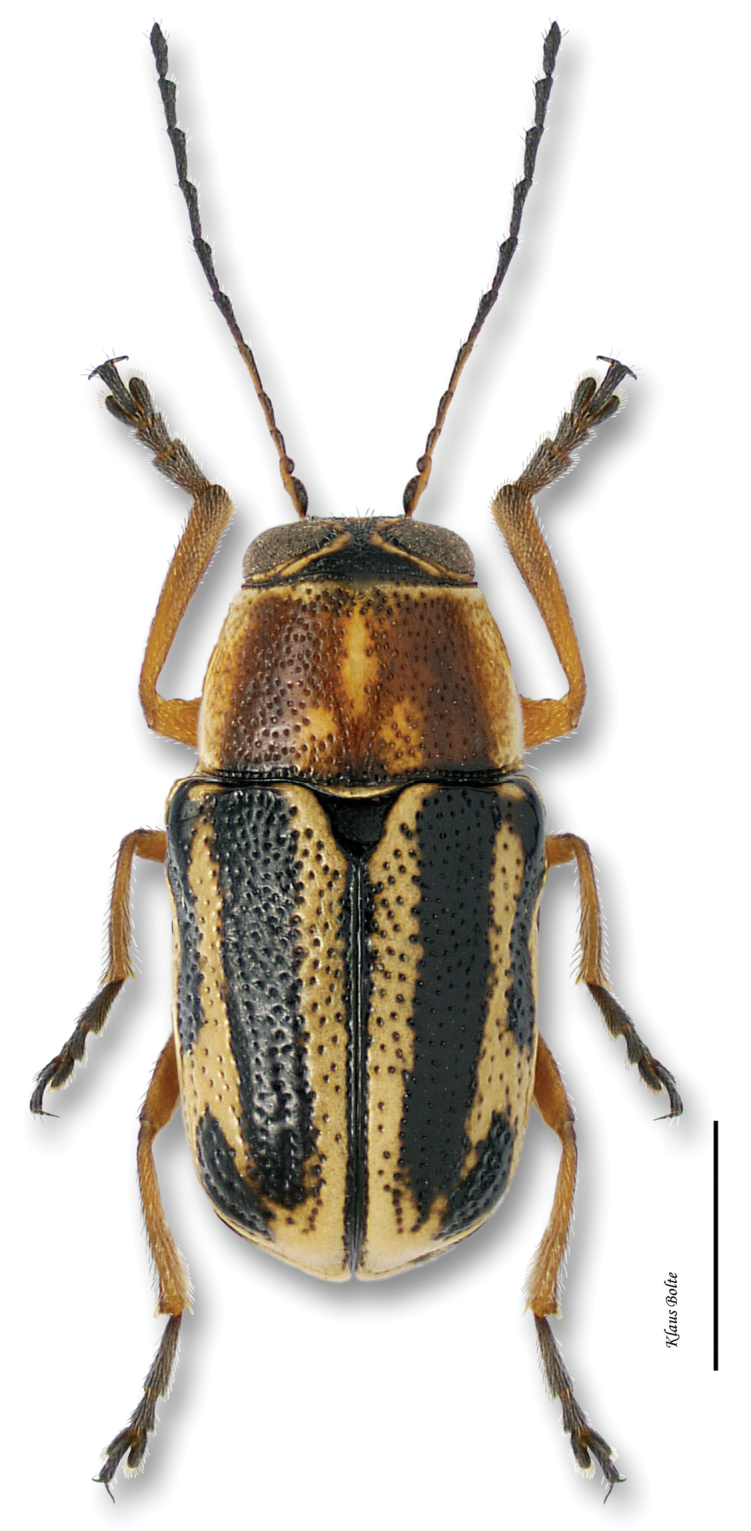
Dorsal habitus of *Pachybrachis bivittatus*. Scale bar, 1 mm.

**Habitus 3.**
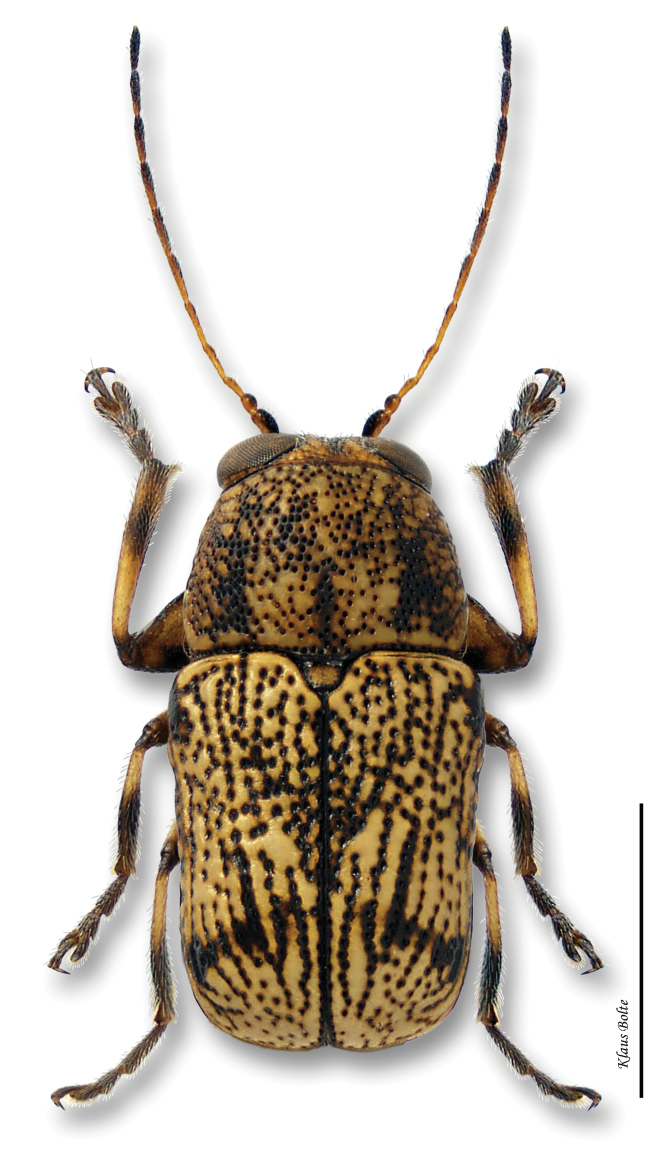
Dorsal habitus of *Pachybrachis calcaratus*. Scale bar, 1 mm.

**Habitus 4.**
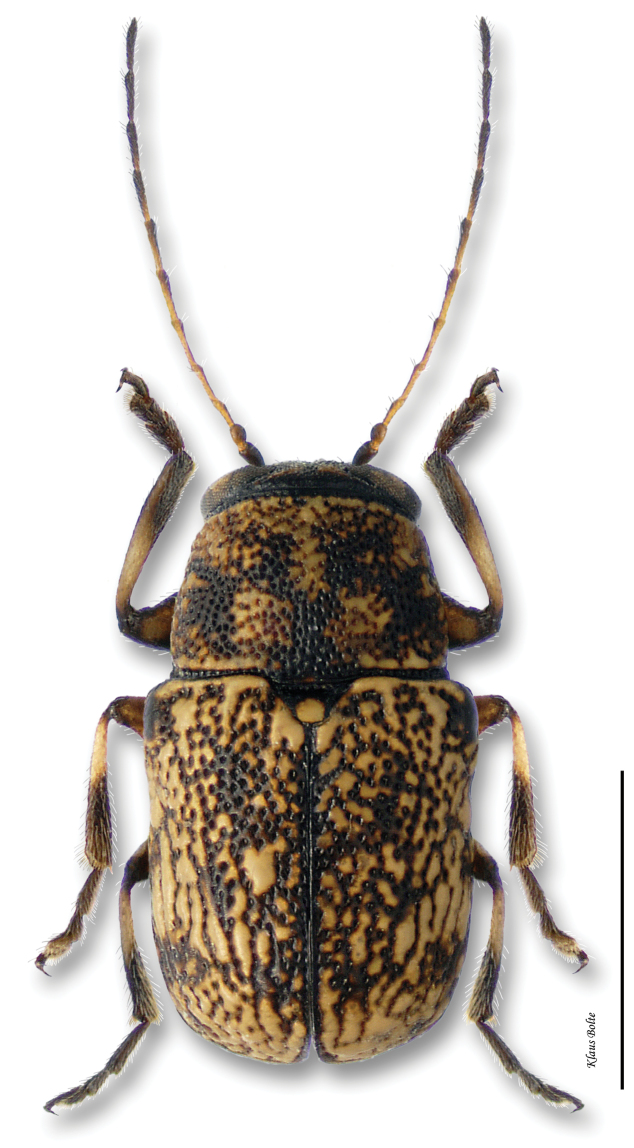
Dorsal habitus of *Pachybrachis cephalicus*. Scale bar, 1 mm.

**Habitus 5.**
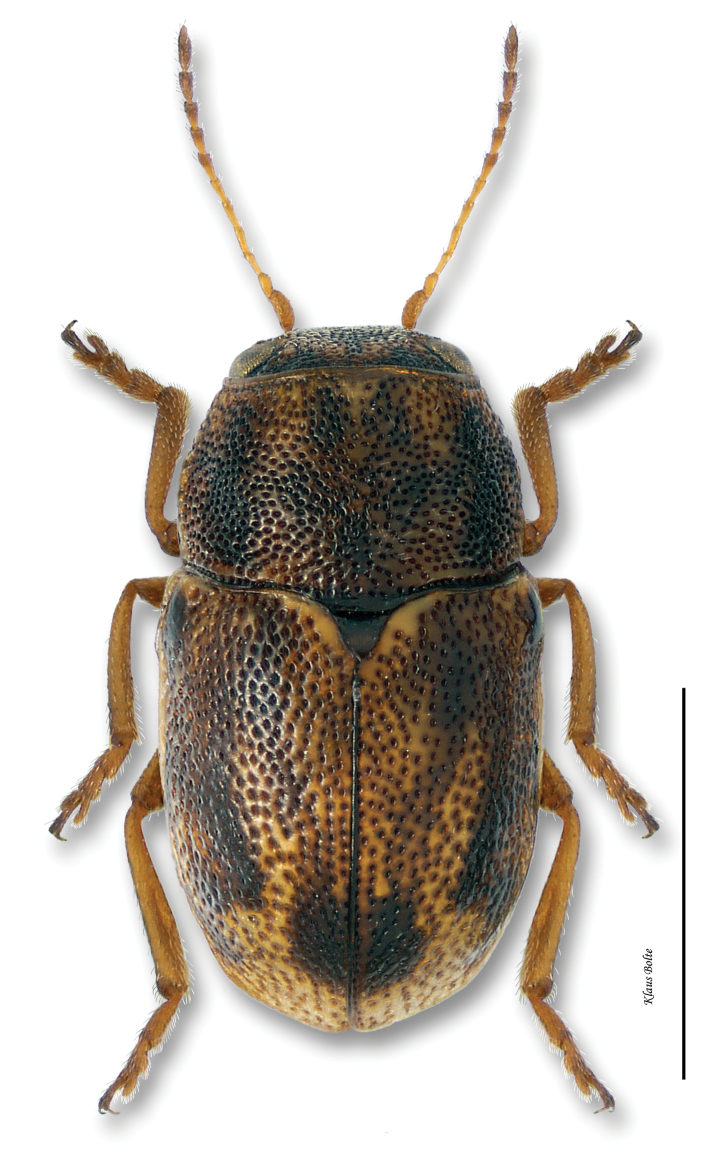
Dorsal habitus of *Pachybrachis hepaticus hepaticus*. Scale bar, 1 mm.

**Habitus 6.**
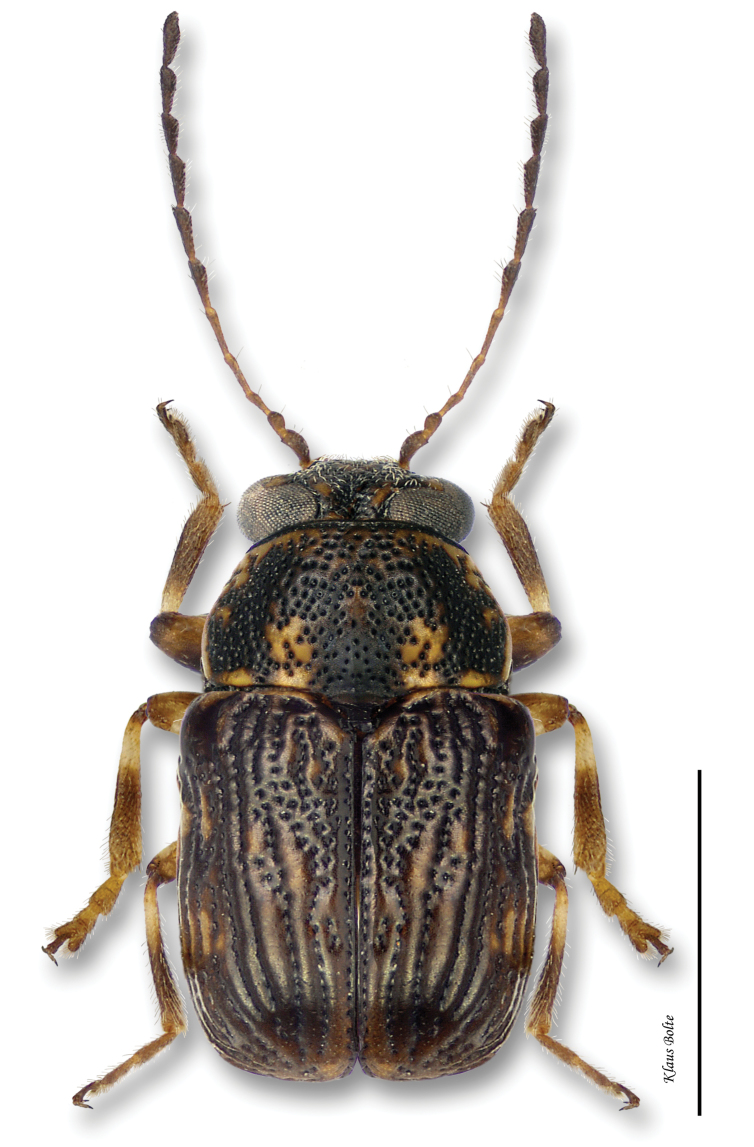
Dorsal habitus of *Pachybrachis luctuosus*. Scale bar, 1 mm.

**Habitus 7.**
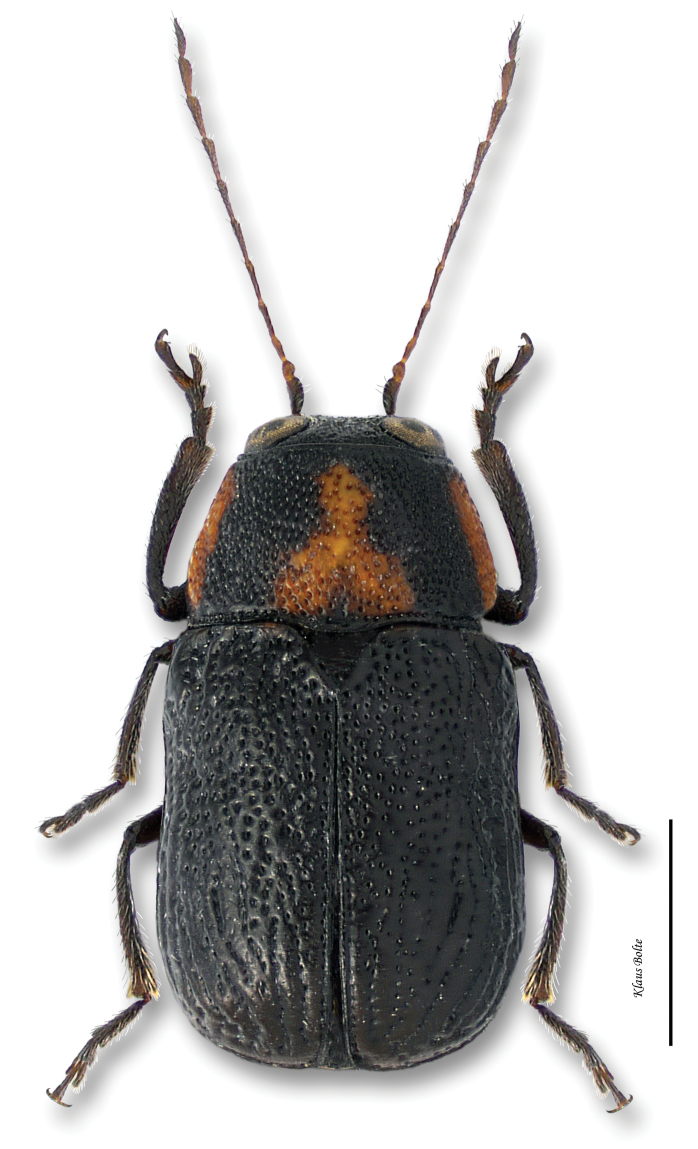
Dorsal habitus of *Pachybrachis luridus*. Scale bar, 1 mm.

**Habitus 8.**
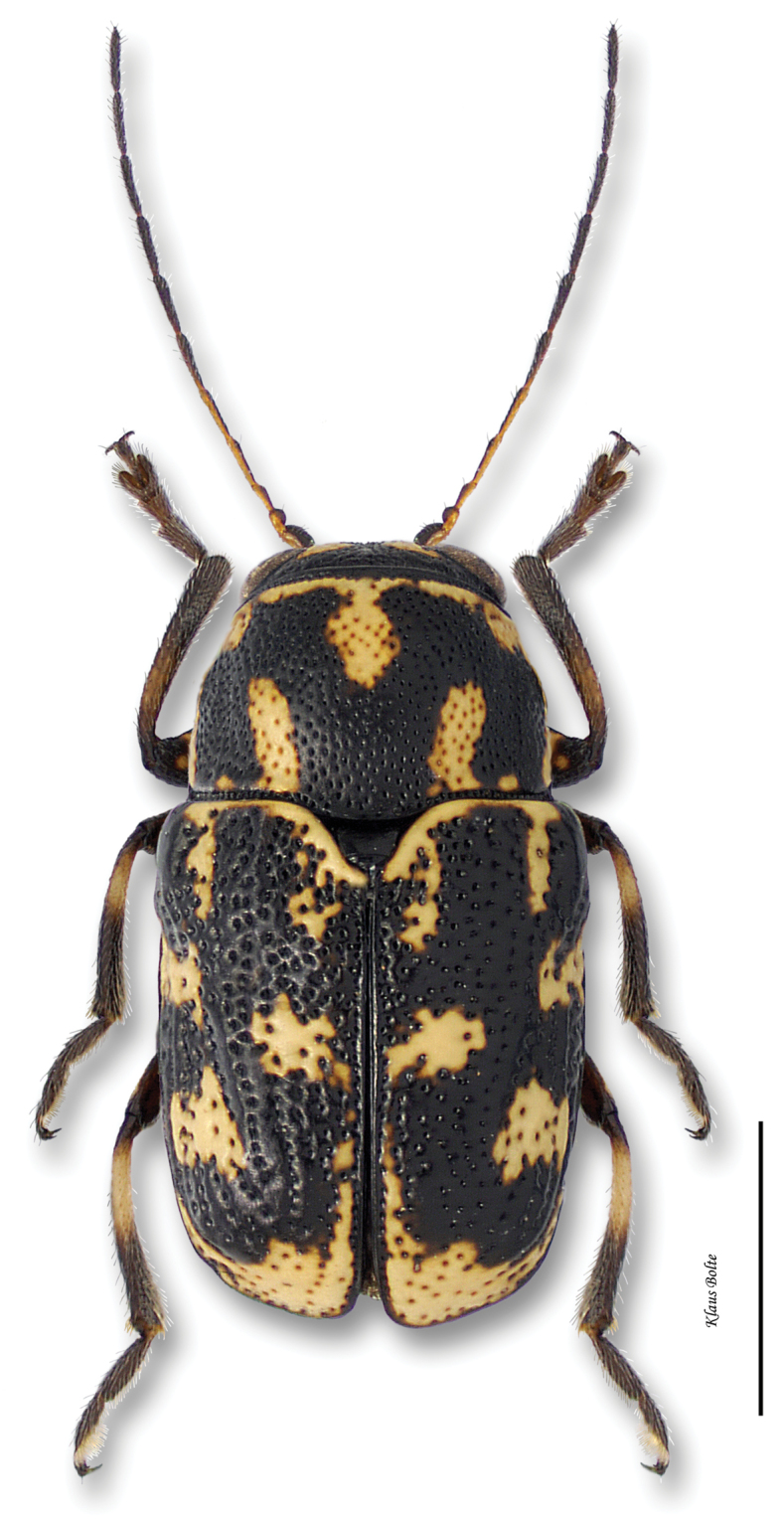
Dorsal habitus of *Pachybrachis m-nigrum*. Scale bar, 1 mm.

**Habitus 9a.**
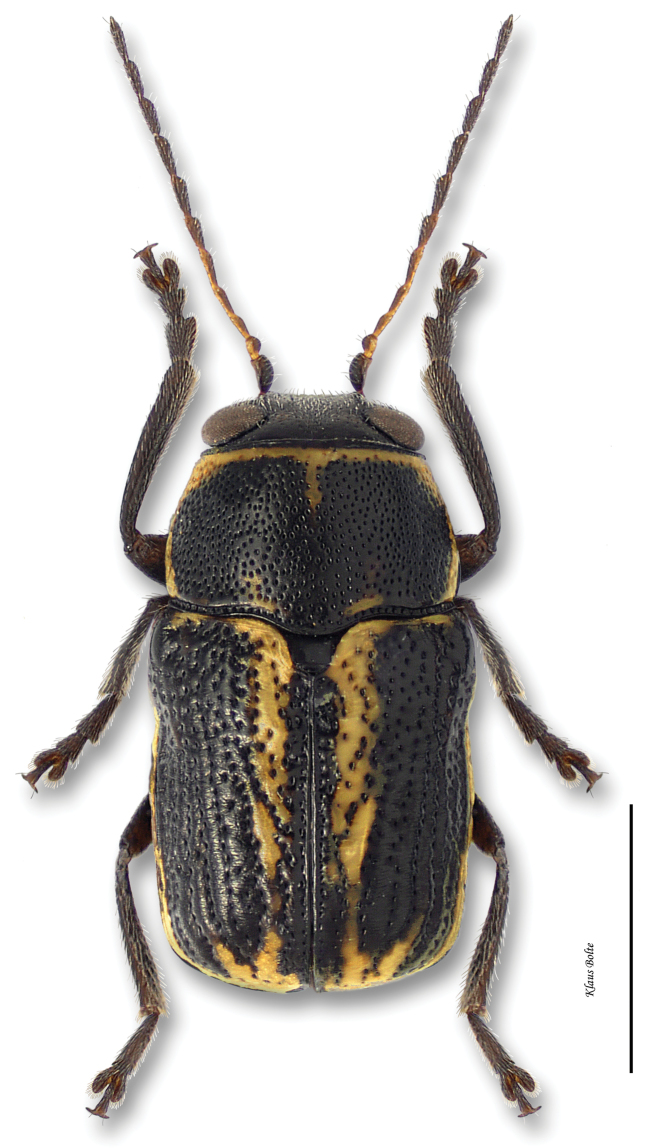
Dorsal habitus of *Pachybrachis nigricornis difficilis*. Scale bar, 1 mrn.

**Habitus 9b.**
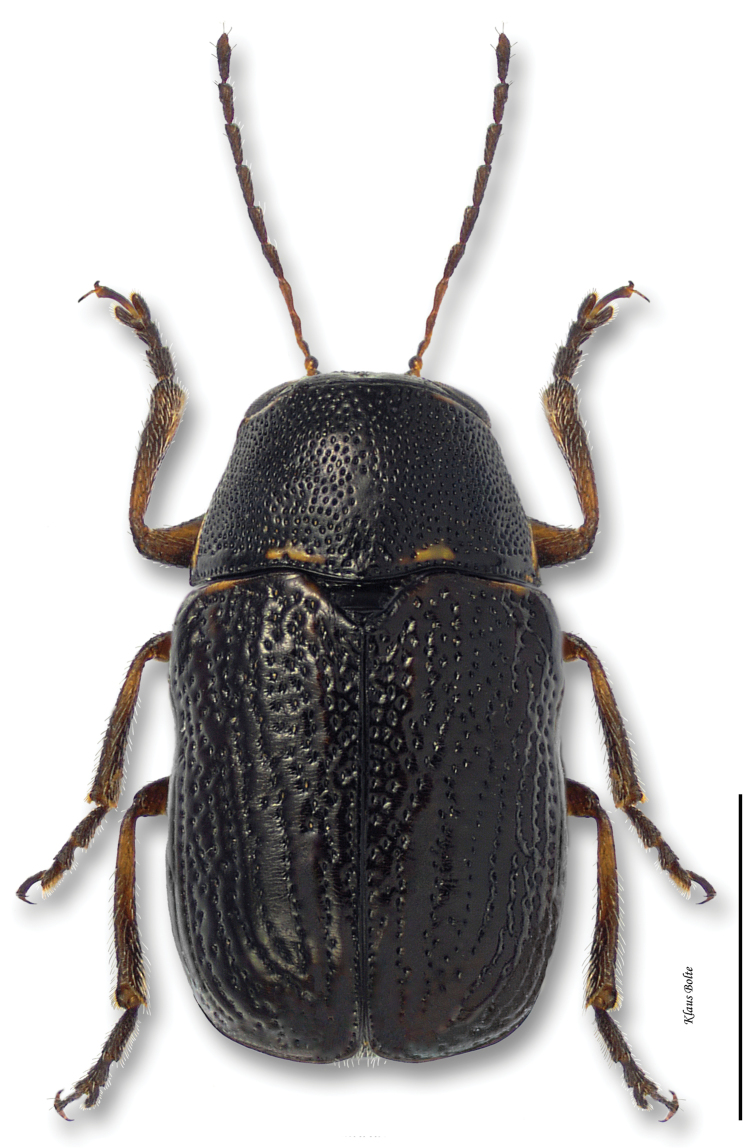
Dorsal habitus of *Pachybrachis nigricornis carbonarius*. Almost black. Scale bar, 1 mm.

**Habitus 9c.**
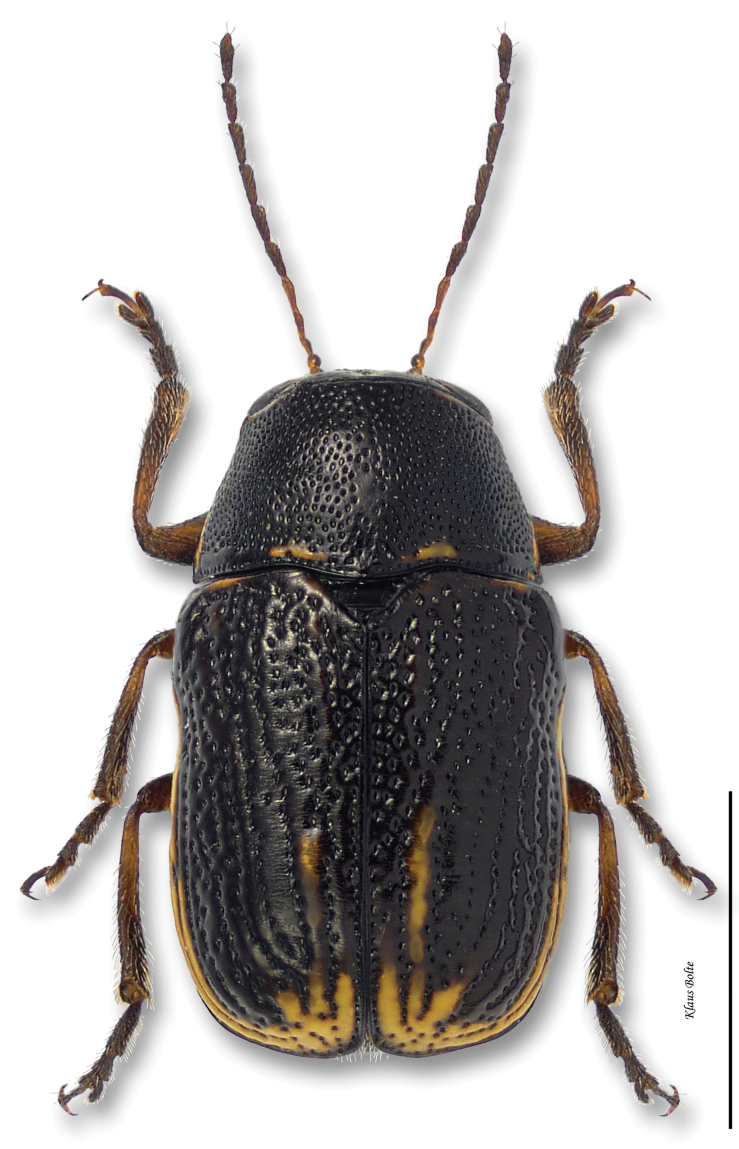
Dorsal habitus of *Pachybrachis nigricornis carbonarius*. Yellow at edge of elytra. Scale bar, 1 mm.

**Habitus 10.**
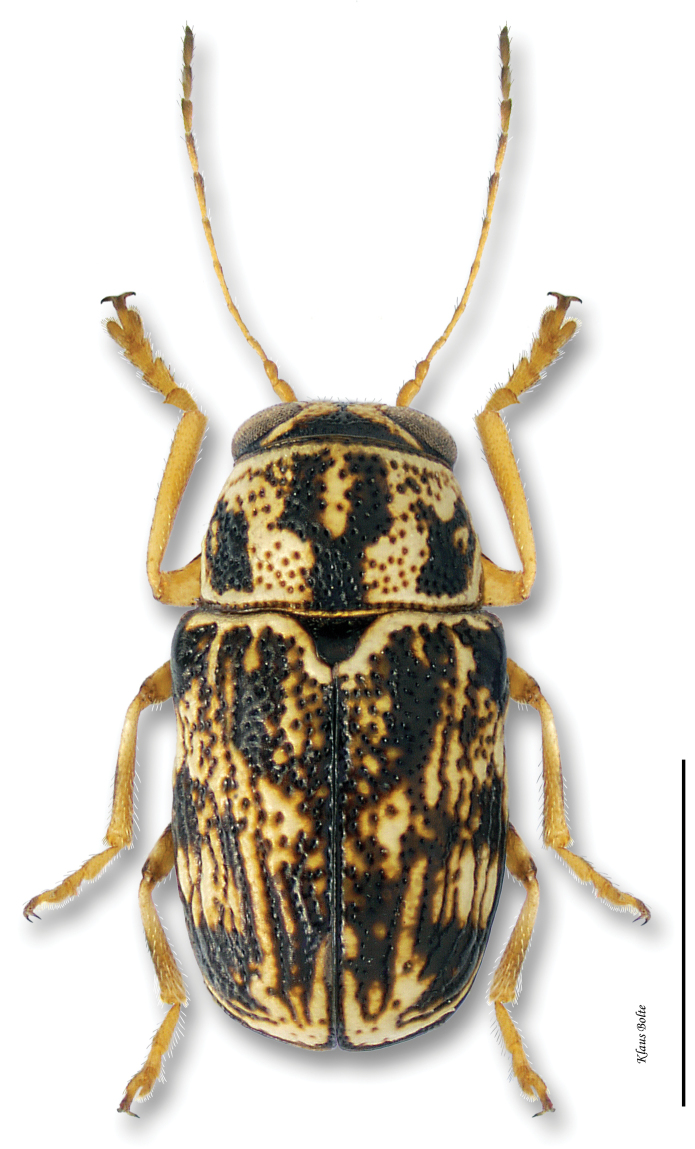
Dorsal habitus of *Pachybrachis obsoletus*. Scale bar, 1 mm.

**Habitus 11.**
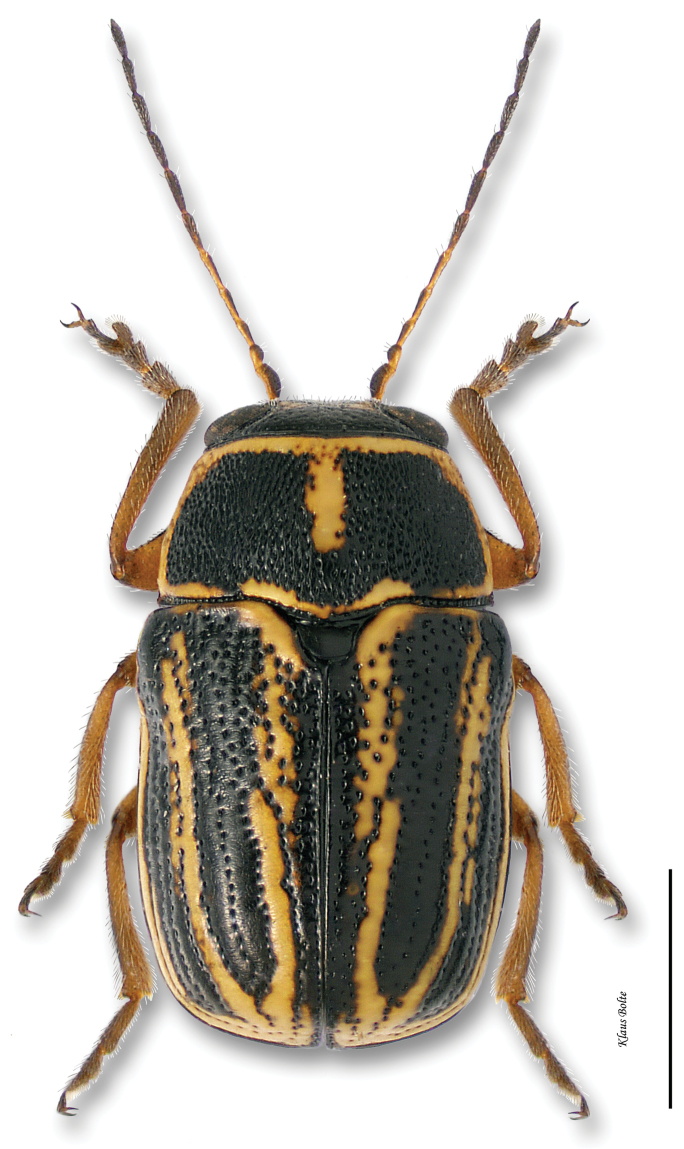
Dorsal habitus of *Pachybrachis othonus othonus*. Scale bar, 1 mm.

**Habitus 12.**
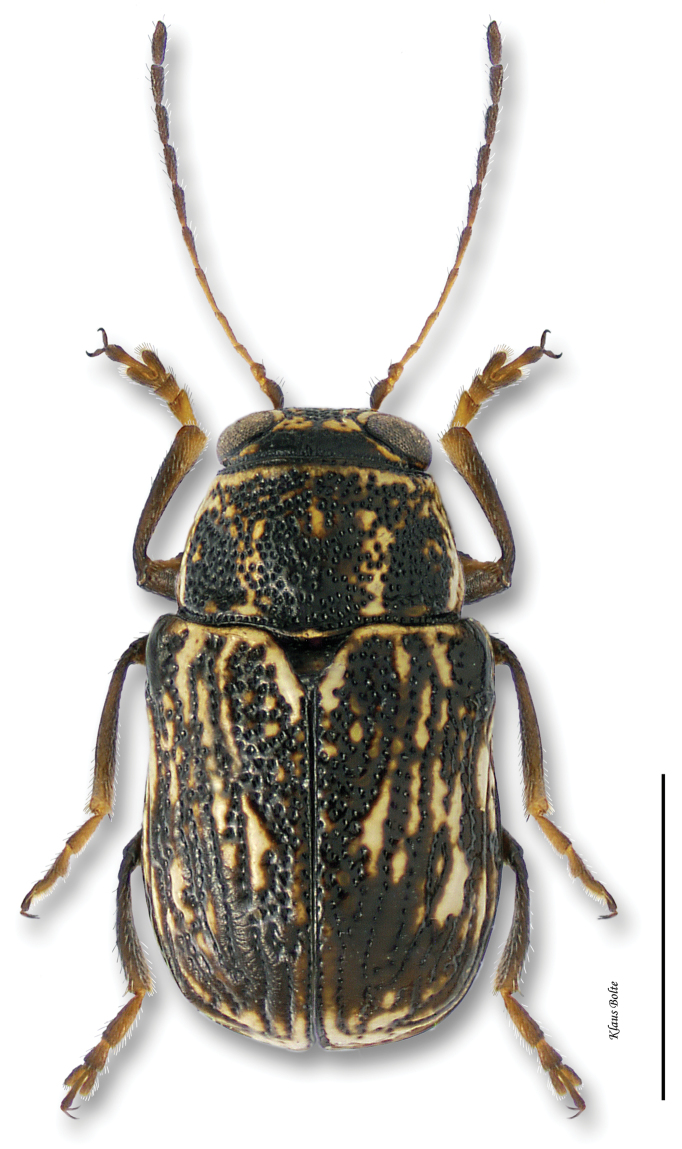
Dorsal habitus of *Pachybrachis peccans*. Scale bar, 1 mn.

**Habitus 13.**
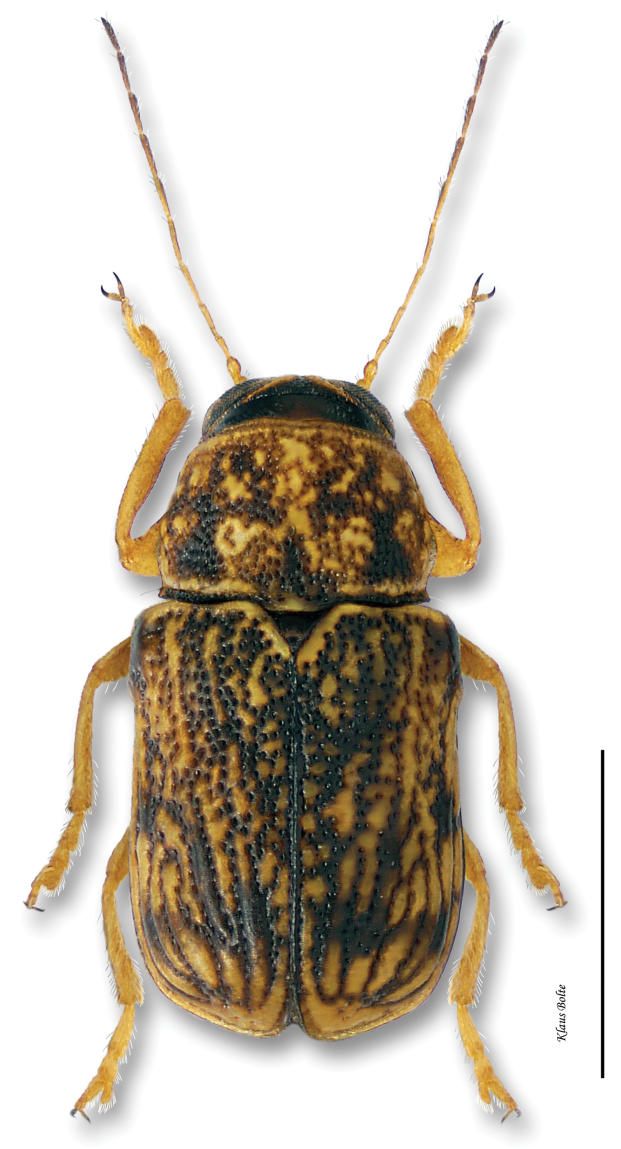
Dorsal habitus of *Pachybrachis pectoralis*. Scale bar, 1 mm.

**Habitus 14.**
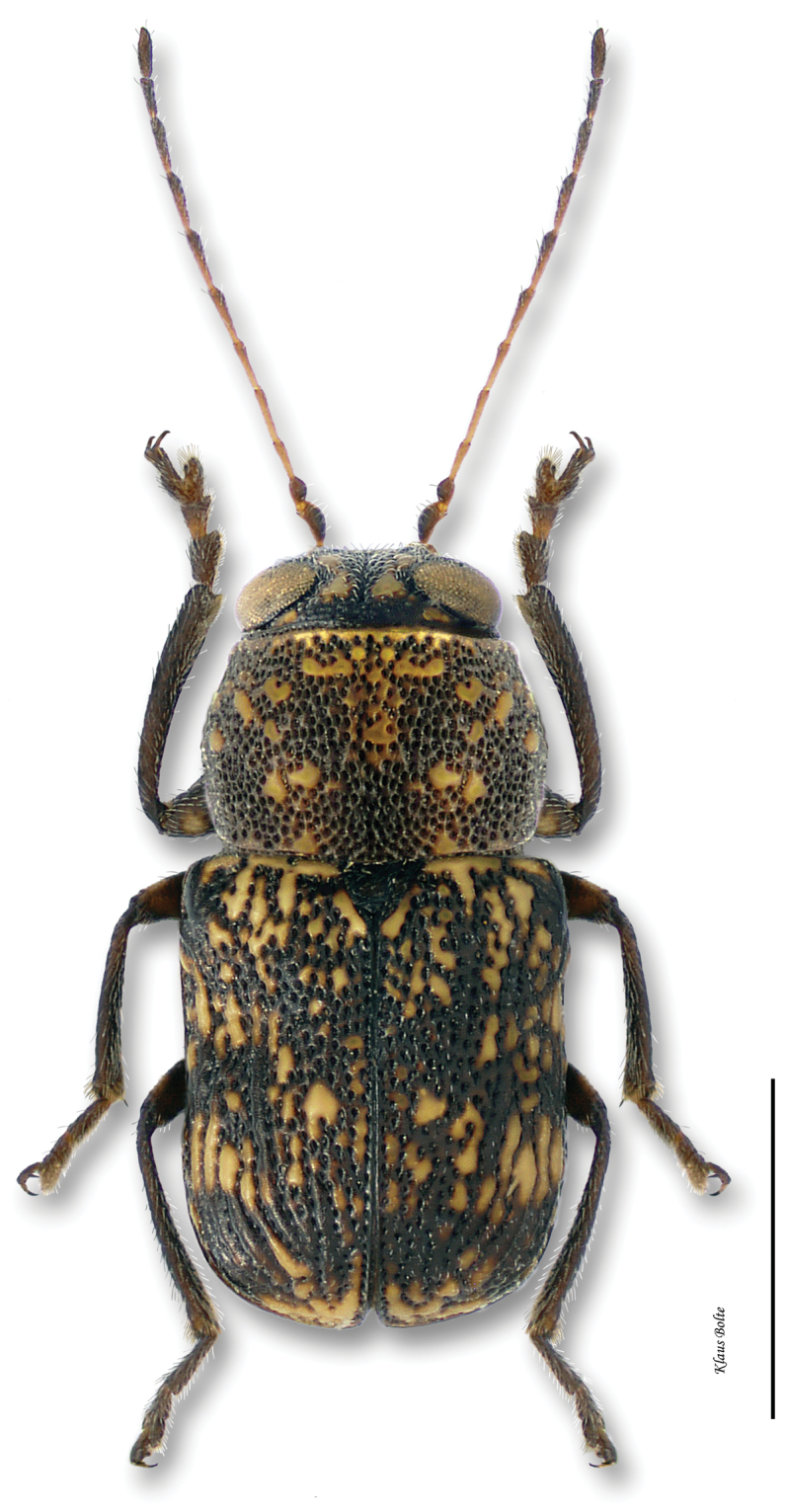
Dorsal habitus of *Pachybrachis spumarius*. Scale bar, 1 mm.

**Habitus 15.**
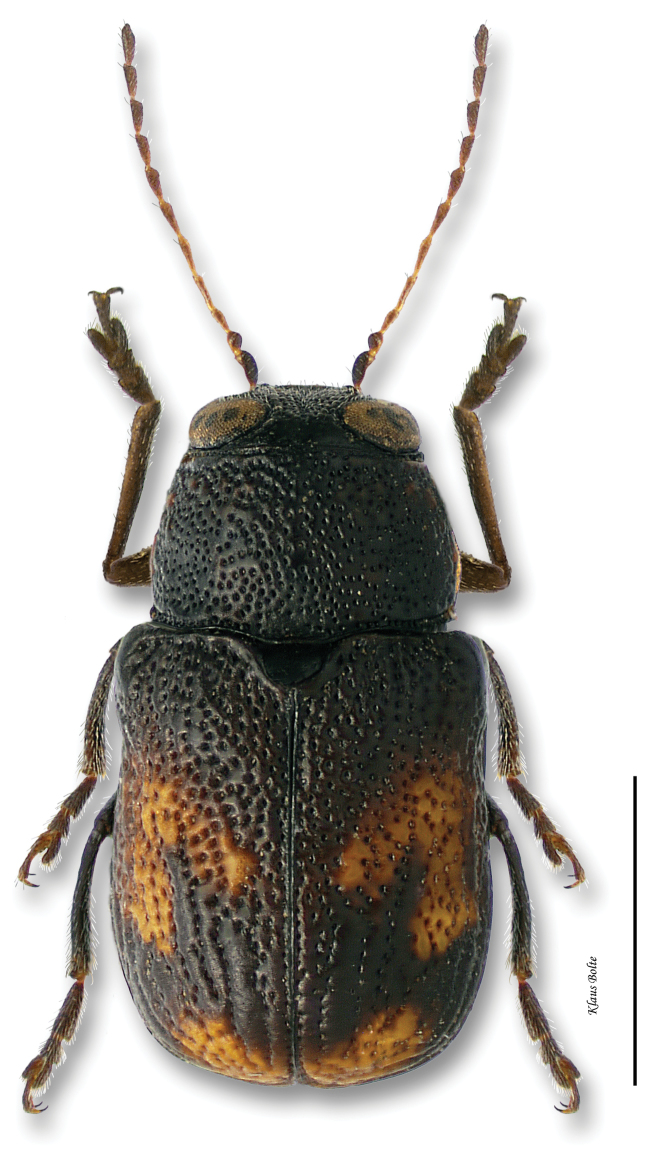
Dorsal habitus of *Pachybrachis subfasciatus*. Scale bar, 1 mm.

**Habitus 16.**
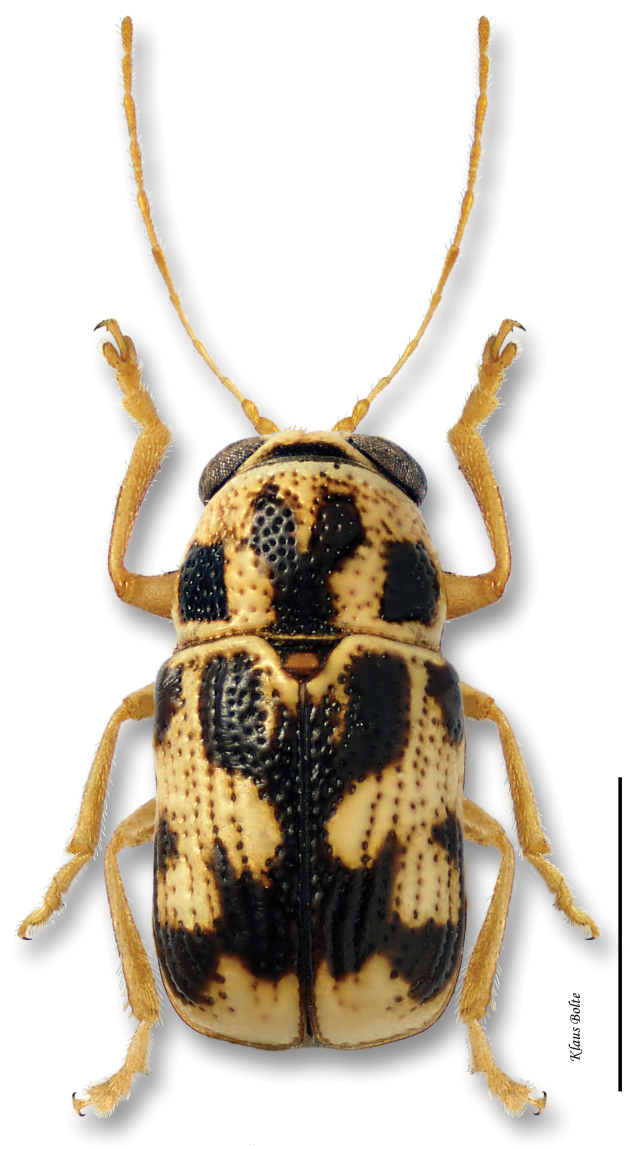
Dorsal habitus of *Pachybrachis tridens*. Scale bar, 1 mm.

**Habitus 17.**
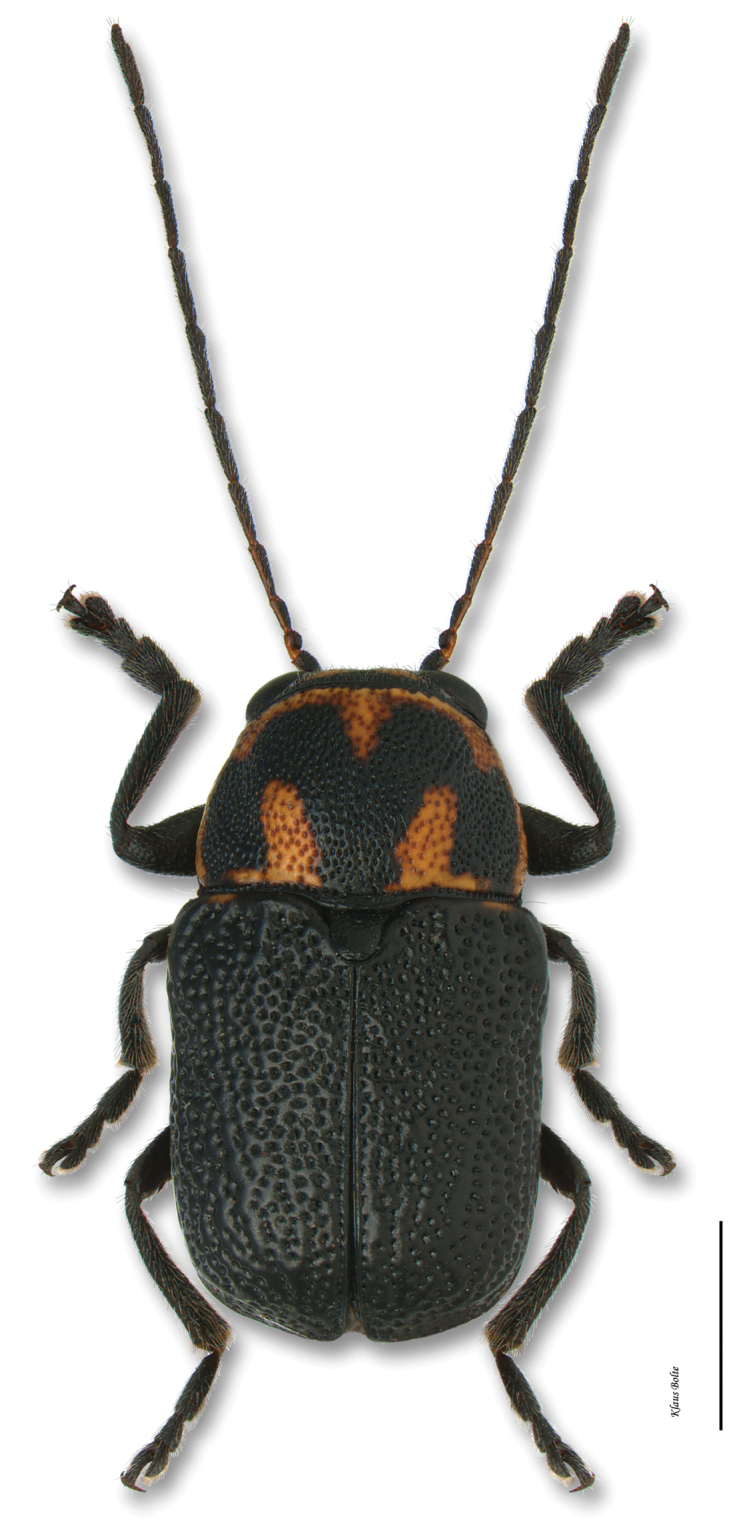
Dorsal habitus of *Pachybrachis trinotatus*. Scale bar, 1 mm.

## Map

## Supplementary Material

XML Treatment for Pachybrachis

XML Treatment for Pachybrachis atomarius

XML Treatment for Pachybrachis bivittatus

XML Treatment for Pachybrachis calcaratus

XML Treatment for Pachybrachis cephalicus

XML Treatment for Pachybrachis hepaticus hepaticus

XML Treatment for Pachybrachis luctuosus

XML Treatment for Pachybrachis luridus

XML Treatment for Pachybrachis m-nigrum

XML Treatment for Pachybrachis nigricornis

XML Treatment for Pachybrachis obsoletus

XML Treatment for Pachybrachis othonus othonus

XML Treatment for Pachybrachis peccans

XML Treatment for Pachybrachis pectoralis

XML Treatment for Pachybrachis spumarius

XML Treatment for Pachybrachis subfasciatus

XML Treatment for Pachybrachis tridens

XML Treatment for Pachybrachis trinotatus
